# Relative timing information and orthology in evolutionary scenarios

**DOI:** 10.1186/s13015-023-00240-4

**Published:** 2023-11-08

**Authors:** David Schaller, Tom Hartmann, Manuel Lafond, Peter F. Stadler, Nicolas Wieseke, Marc Hellmuth

**Affiliations:** 1 Bioinformatics Group, Department of Computer Science, and Interdisciplinary Center for Bioinformatics, Universität Leipzig, Härtelstraße 16-18, Leipzig, 04107 Germany; 2 Department of Mathematics, Faculty of Science, Stockholm University, Stockholm, 10691 Sweden; 3 Department of Computer Science, Université de Sherbrooke, 2500 boul. de l’Université, Sherbrooke, J1K 2R1 Canada; 4 Swarm Intelligence and Complex Systems Group, Faculty of Mathematics and Computer Science, Leipzig University, Augustusplatz 10, Leipzig, 04109 Germany; 5 Competence Center for Scalable Data Services and Solutions Dresden/Leipzig, Interdisciplinary Center for Bioinformatics, German Centre for Integrative Biodiversity Research (iDiv), and Leipzig Research Center for Civilization Diseases, Universität Leipzig, Augustusplatz 12, Leipzig, 04107 Germany; 6 Max Planck Institute for Mathematics in the Sciences, Inselstraße 22, Leipzig, 04109 Germany; 7 Department of Theoretical Chemistry, University of Vienna, Währinger Straße 17, Vienna, 1090 Austria; 8 Facultad de Ciencias, Universidad National de Colombia, Sede Bogotá, Ciudad Universitaria, Bogotá, 111321 DC Colombia; 9 Santa Fe Institute, 1399 Hyde Park Rd., Santa Fe, NM87501 USA

**Keywords:** Gene tree, Species tree, Cograph, Perfect graph, Orthology, Xenology, Horizontal gene transfer, Informative and forbidden triples, Relative timing, NP-hardness

## Abstract

**Background:**

Evolutionary scenarios describing the evolution of a family of genes within a collection of species comprise the mapping of the vertices of a gene tree *T* to vertices and edges of a species tree *S*. The relative timing of the last common ancestors of two extant genes (leaves of *T*) and the last common ancestors of the two species (leaves of *S*) in which they reside is indicative of horizontal gene transfers (HGT) and ancient duplications. Orthologous gene pairs, on the other hand, require that their last common ancestors coincides with a corresponding speciation event. The relative timing information of gene and species divergences is captured by three colored graphs that have the extant genes as vertices and the species in which the genes are found as vertex colors: the equal-divergence-time (EDT) graph, the later-divergence-time (LDT) graph and the prior-divergence-time (PDT) graph, which together form an edge partition of the complete graph.

**Results:**

Here we give a complete characterization in terms of informative and forbidden triples that can be read off the three graphs and provide a polynomial time algorithm for constructing an evolutionary scenario that explains the graphs, provided such a scenario exists. While both LDT and PDT graphs are cographs, this is not true for the EDT graph in general. We show that every EDT graph is perfect. While the information about LDT and PDT graphs is necessary to recognize EDT graphs in polynomial-time for general scenarios, this extra information can be dropped in the HGT-free case. However, recognition of EDT graphs without knowledge of putative LDT and PDT graphs is NP-complete for general scenarios. In contrast, PDT graphs can be recognized in polynomial-time. We finally connect the EDT graph to the alternative definitions of orthology that have been proposed for scenarios with horizontal gene transfer. With one exception, the corresponding graphs are shown to be colored cographs.

## Introduction

An *evolutionary scenario* describes the history of a gene family relative to the phylogeny of a set of species. Formally, it comprises a mapping $\mu$ of the gene tree *T* into the species tree *S*, usually called the *reconciliation* of *S* and *T*. The conceptual relevance of scenarios in evolutionary biology derives from the fact that they define key relationships between genes, in particular orthology, paralogy, and xenology [1]. On the practical side, scenarios also imply relations on the set of genes that can be inferred directly from sequence similarity data, such as the *best match* relation [2, 3] or the *later divergence time* (LDT) relation [4], which is closely related to the inference of horizontal gene transfer (HGT) events.

In the absence of horizontal transfer, orthology is characterized by the fact that the last common ancestor of two genes *x* and *y* is exactly the speciation event that separated the two species $\sigma (x)$ and $\sigma (y)$ in which *x* and *y*, resp., reside [1]. A necessary condition for orthology, therefore, is that the last common ancestor of the genes *x* and *y* and the last common ancestor of the species $\sigma (x)$ and $\sigma (y)$ have the same evolutionary age. Whether or not *x*, *y* and $\sigma (x),\sigma (y)$ have *equal divergence time* (EDT) can be decided (at least at some level of accuracy) directly from sequence data. The graph $G_{_{=}}$ whose vertices are the genes and whose edges are the pairs of genes with equal divergence time of *x*, *y* and $\sigma (x),\sigma (y)$ thus is an empirically accessible datum. By construction, furthermore, the EDT graph contains the orthology graph as a subgraph.

**Fig. 1:**
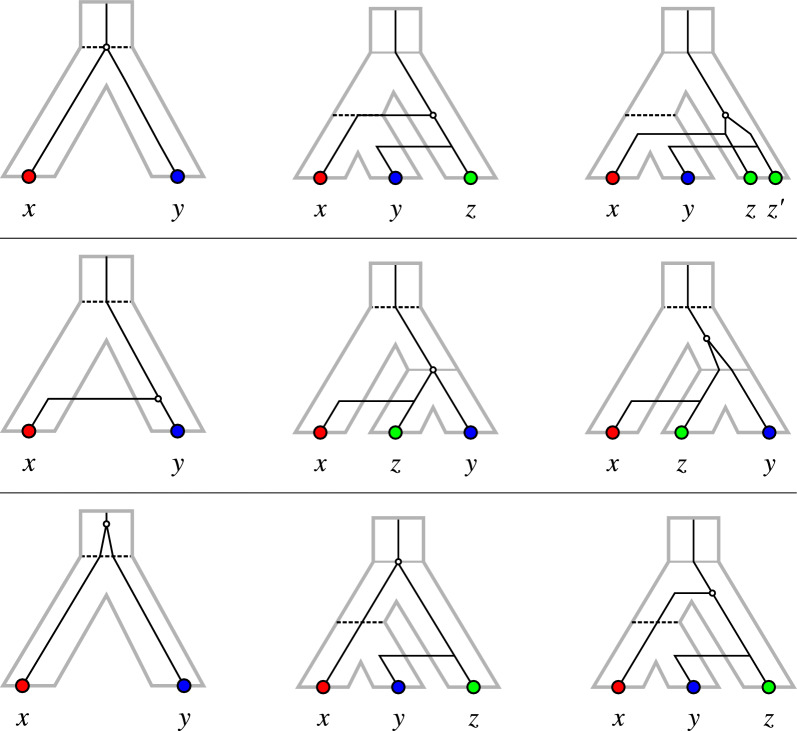
Examples of evolutionary scenarios depicted as gene trees (black inline trees) embedded into species trees (gray outline trees). In all cases, the ancestral gene ${{\,\textrm{lca}\,}}_T(x,y)$ of *x* and *y* is highlighted as white circle while the corresponding species ${{\,\textrm{lca}\,}}_S(\sigma (x),\sigma (y))$ is highlighted as dashed line. *Top row:* scenario with *x* and *y* in EDT relation, i.e., the ancestral gene ${{\,\textrm{lca}\,}}_T(x,y)$ diverged concurrently with the corresponding species ${{\,\textrm{lca}\,}}_S(\sigma (x),\sigma (y))$. The evolutionary event at ${{\,\textrm{lca}\,}}_T(x,y)$ is either a speciation (left), a horizontal gene transfer (center), or a duplication (right). *Middle row:* scenario with *x* and *y* in LDT relation, i.e., the ancestral gene ${{\,\textrm{lca}\,}}_T(x,y)$ diverged after the corresponding species ${{\,\textrm{lca}\,}}_S(\sigma (x),\sigma (y))$. The evolutionary event at ${{\,\textrm{lca}\,}}_T(x,y)$ is either a horizontal gene transfer (left), a speciation (center), or a duplication (right). *Bottom row:* scenario with *x* and *y* in PDT relation, i.e., the ancestral gene ${{\,\textrm{lca}\,}}_T(x,y)$ diverged before the corresponding species ${{\,\textrm{lca}\,}}_S(\sigma (x),\sigma (y))$. The evolutionary event at ${{\,\textrm{lca}\,}}_T(x,y)$ is either a duplication (left), a speciation (center), or a horizontal gene transfer (right)

The LDT and EDT relations can be complemented with a “prior divergence time” relation (PDT). Together, the EDT, LDT and PDT relations then define a 3-partition $\mathcal {G}$ of the edge set of a complete graph with the genes as vertices. Since the EDT relation has some connection with orthology and the LDT relation with xenology, it seems intuitive that the PDT relation might be connected with paralogy. However, for none of the three relations this connection is strict in the sense that it would enforce a particular type of evolutionary event at the corresponding last common ancestor. Figure 1 shows examples of evolutionary scenarios with genes in EDT relation (top row), LDT relation (middle row) and PDT relation (bottom row) with the corresponding last common ancestor being any of the event types speciation, HGT, and duplication. The EDT, LDT, and PDT relations are therefore distinct from the orthology, xenology, and paralogy relations considered in [5]. Nevertheless, the relative timing information from the last common ancestors of pairs of extant genes can be used to construct the topologies of the underlying gene and species tree as well as a reconciliation between them. The reconciliation then determines the orthology, xenology, and paralogy relations. The reconciliation, however, is in general not uniquely determined by the 3-partition $\mathcal {G}$.

We show here that a collection of *informative* and *forbidden triples* defined by $\mathcal {G}$ are the key criteria to determine whether or not $\mathcal {G}$ derives from a scenario $\mathcal {S}$. While both LDT and PDT graphs are cographs, this is not always the case for the EDT graph. We shall see, however, that it is a cograph if both *T* and *S* are binary (fully resolved) trees. In Section “Explanation of $\mathcal {G}$ by Relaxed Scenarios” we derive a quartic time algorithm for the recognition of edge-tripartitions that derive from a corresponding scenario. This construction is then used to give a triple-based characterization. We then show that the existence of an explaining scenario is sufficient to guarantee that $\mathcal {G}$ can also be explained by scenarios with several additional desirable properties. Importantly, these restricted scenarios have properties that are often assumed for valid reconciliations of *T* and *S* in the literature. For instance, it is possible to choose the scenarios such that each event (inner node of *T*) has at least one purely vertical descendant; this is the case for all scenarios in Fig. 1. In Section “Orthology and Quasi-Orthology”, EDT graphs are connected with several competing notions of “orthology” proposed by different authors [1, 6–8].

## Notation

*Graphs* We consider undirected simple graphs $G=(V,E)$ with vertex set $V(G){:=}V$ and edge set $E(G){:=}E$. We write $G\subseteq H$ if $G=(V,E)$ is a subgraph of $H=(V', E')$, i.e., if $V\subseteq V'$ and $E\subseteq E'$. The subgraph of *G* that is induced by the subset $X \subseteq V$ will be denoted by *G*[*X*]. A connected component *C* of *G* is an inclusion-maximal subset $C\subseteq V$ such that *G*[*C*] is connected. The complement of a graph $G=(V,E)$ is the graph $\overline{G}=(V,\overline{E})$ with vertex set *V* and an edge $xy\in \overline{E}$ for $x\ne y$ precisely if $xy\notin E$. We denote by $K_n$ the graph on *n* vertices in which every possible edge is present, hereafter called a *complete graph*. A graph property $\Pi$ is a subset of the set of all graphs. A graph property $\Pi$ is *closed under complementation* if $G\in \Pi$ implies $\overline{G}\in \Pi$.

*Rooted trees* Trees are connected and acyclic graphs. All trees in this contribution have a distinguished vertex $\rho$, called the *root* of the tree. For two vertices $x,y\in V(T)$, we write $y \preceq _{T} x$ if *x* lies on the unique path from the root to *y*, in which case *x* is called an *ancestor* of *y*, and *y* is called a *descendant* of *x*. If, in addition, *x* and *y* are adjacent in *T*, then *x* is the *parent* of *y* (denoted by ${{\,\textrm{par}\,}}_T(y)$), and *y* is a *child* of *x*. The set of children of *x* is denoted by ${{\,\textrm{child}\,}}_T(x)$. We write edges $e=xy$ indicating that $y\preceq _T x$. It will be convenient to extend the relation $\preceq _{T}$ to the union $V(T)\cup E(T)$ as follows: For a vertex $x\in V(T)$ and an edge $e=uv\in E(T)$, we set $x \preceq _T e$ if and only if $x\preceq _T v$; and $e \preceq _T x$ if and only if $u\preceq _T x$. In addition, for edges $e=uv$ and $f=ab$ in *T*, we put $e\preceq _T f$ if and only if $v \preceq _T b$ (note that under this definition, $uv \preceq _T uv$). For $x,y\in V(T)\cup E(T)$, we may also write $x \succeq _{T} y$ instead of $y \preceq _{T} x$. We use $y \prec _T x$ for $y \preceq _{T} x$ and $x \ne y$. Moreover, we say that *x* and *y* are *comparable* if $y\preceq _{T} x$ or $x\preceq _{T} y$ holds and, otherwise, *x* and *y* are *incomparable*. Note that $\preceq _{T}$ is a partial order with a unique maximal element $\rho$. The *leaves* $L=L(T)\subseteq V(T)$ of *T* are precisely the $\preceq _{T}$-minimal elements.

From here on, we assume that the root $\rho$ as well as every non-leaf vertex of a tree have always at least two children. Moreover, we write *T*(*u*) for the *subtree of* *T* *rooted at* *u*, i.e, the tree that is induced by *u* and all its descendants.

For a set of leaves $A\subseteq L$, we write ${{\,\textrm{lca}\,}}_T(A)$ for the *last common ancestor* of *A*, i.e., the unique $\preceq _T$-minimal vertex in *V*(*T*) such that $x\preceq {{\,\textrm{lca}\,}}_T(A)$ for all $x\in A$. For simplicity, we write ${{\,\textrm{lca}\,}}_T(x,y)$ instead of ${{\,\textrm{lca}\,}}_T(\{x,y\})$. The *restriction of* *T* to a subset $L'\subseteq L$, in symbols $T_{\vert L'}$, is obtained from the minimal subtree of *T* that connects all leaves in $L'$ by suppressing all vertices with degree two except possibly the root $\rho _{T_{\vert L'}}$. We often write $T_{\vert x_1\dots x_k}$ instead of $T_{\vert \{x_1,\dots ,x_k\}}$. A tree *T* *displays* a tree $T'$ with $L(T')\subseteq L(T)$ if $T'$ is isomorphic to $T_{\vert L(T')}$.

*Planted trees* In order to accommodate evolutionary events pre-dating $\rho {:=}{{\,\textrm{lca}\,}}(L)$, we consider *planted trees*, i.e., we assume an additional planted root $0_T$ with degree 1 that is the parent of the “root” $\rho$. The *inner vertices* of *T* are $V^0(T) {:=}V(T) {\setminus } (L(T) \cup \{0_T\})$. In particular, a planted tree *T* always displays the rooted tree $T_{\vert L(T)}$ obtained by removing $0_T$ and its incident edge $0_T\rho$.

### Remark

Unless explicitly stated otherwise, the trees that appear in this contribution are planted phylogenetic trees, i.e., $0_T$ is the only vertex with exactly one child. All other vertices are either leaves or have at least two children.

*Triples and fan triples* A (rooted) triple is a binary rooted tree on three vertices. We denote by $xy\vert z$ the rooted triple *t* with leaf set $\{x,y,z\}$ and ${{\,\textrm{lca}\,}}_t(x,y) \prec _T {{\,\textrm{lca}\,}}_t (x,z) = {{\,\textrm{lca}\,}}_t(y,z)$. A tree *T* displays $xy\vert z$ if ${{\,\textrm{lca}\,}}_T(x,y) \prec _T {{\,\textrm{lca}\,}}_T (x,z) = {{\,\textrm{lca}\,}}_T(y,z)$. A *fan triple* $x\vert y\vert z$ on leaves *x*, *y*, *z* is the tree (*x*, *y*, *z*). A tree *T* displays the fan triple $x\vert y\vert z$ if ${{\,\textrm{lca}\,}}_T(x,y) = {{\,\textrm{lca}\,}}_T(x,z) = {{\,\textrm{lca}\,}}_T(y,z)$.

As usual, we say that a set $\mathcal {R}$ of triples is *consistent* if there is a tree *T* that displays all of the triples in $\mathcal {R}$. If $(\mathcal {R},\mathcal {F})$ is a pair of two triple sets, we say that $(\mathcal {R},\mathcal {F})$ is consistent if there is a tree *T* that displays all of the triples in $\mathcal {R}$ but none of the triples in $\mathcal {F}$. In this case, we say that *T* *agrees with* $(\mathcal {R},\mathcal {F})$. We will frequently make use of the following simple observation that collects the structures of the subtree $T_{\vert L'\cup L''}$ on $\vert L'\cup L''\vert =4$ leaves implied by two subtrees $T_{\vert L'}$ and $T_{\vert L''}$ on three leaves (triples) sharing $\vert L'\cap L''\vert =2$ common leaves. The statements are closely related to the so-called “inference rules” for rooted triples, see in particular [9, 10]. We leave the elementary proofs to the interested reader. We use Newick notation for rooted trees, i.e., inner vertices correspond to matching parentheses, leaves are given by their labels, and commas are used to separate sibling to increase readability. For example, the triple $ab\vert c$ is equivalently represented as ((*a*, *b*), *c*).

### Observation 1

Let *T* be a tree and $a,b,c,d\in L(T)$ be pairwise distinct leaves. Suppose *T* displays $ab\vert c$.

(i) If *T* displays $cd\vert a$, then $T_{\vert abcd}=((a,b),(c,d))$.
(ii) If *T* displays $ac\vert d$, then $T_{\vert abcd} = (((a,b),c),d)$.
(iii) If *T* displays $ad\vert c$, then *T* displays $bd\vert c$ and $T_{\vert abcd}$ is one of the trees (((*a*, *d*), *b*), *c*), (((*b*, *d*), *a*), *c*), (((*a*, *b*), *d*), *c*), or ((*a*, *b*, *d*), *c*).
(iv) If *T* displays $ab\vert d$, then $T_{\vert abcd}$ is one of the trees (((*a*, *b*), *c*), *d*), (((*a*, *b*), *d*), *c*), ((*a*, *b*), *c*, *d*), or ((*a*, *b*), (*c*, *d*)).
(v) If $$T_{\vert bcd}=(b,c,d)$$, then $$T_{\vert abcd}=((a,b),c,d)$$.

Suppose that *T* does not display any of the triples on $\{a,b,c\}$, i.e., $T_{\vert abc}=(a,b,c)$.

(vi) If $T_{\vert bcd}=(b,c,d)$, then $T_{\vert abcd}=(a,b,c,d)$ or $T_{\vert abcd}=((a,d),b,c)$.

We will make use of Obs. 1 throughout the subsequent proofs without explicit reference.

*Cographs* The *join* of two graphs $G=(V,E)$ and $H=(W,F)$ with disjoint vertex sets $V\cap W=\emptyset$ is the graph $G{{\,\mathrm{\triangledown }\,}}H$ with vertex set  and edge set . Similarly, their *disjoint union* has vertex set  and edge set . *Cographs* are recursively defined as the graphs that either are $K_1$s or can be obtained from the join or disjoint union of two cographs. Cographs have been studied extensively. We summarize some basic results in the next proposition.

### Proposition 1

[11] Given an undirected graph *G*, the following statements are equivalent:

1. *G* is a cograph.
2. *G* is explained by a *cotree* (*T*, *t*), i.e., a rooted tree *T* with $L(T)=V(G)$ and $t:V^0(T)\rightarrow \{0,1\}$ such that $xy\in E(G)$ precisely if $t({{\,\textrm{lca}\,}}_T(x,y))=1$.
3. The complement graph ${\overline{G}}$ of *G* is a cograph.
4. *G* does not contain a $P_4$, i.e., a path on four vertices, as an induced subgraph.
5. Every induced subgraph *H* of *G* is a cograph.

## Equal divergence time graphs

### Evolutionary scenarios

The vertices in phylogenetic trees designate evolutionary events such as speciations, gene duplications, or horizontal gene transfers. Conceptually, any such event *x* is associated with a specific point in time $\tau _{T}(x)$.

#### Definition 1

Let *T* be a rooted or planted tree. Then $\tau _{T}:V(T)\rightarrow \mathbb {R}$ is a time map for *T* if $x\prec _T y$ implies $\tau _{T}(x)<\tau _{T}(y)$. The tuple $(T,\tau _{T})$ is called *dated tree*.

Definition 1 ensures that the ancestor relation $x\prec _T y$ and the timing of the vertices are not in conflict. It also pertains to arbitrary rooted trees since these can be seen as restrictions of planted trees to $V\setminus \{0_T\}$. Note that for an edge *uv* of *T*, the convention that *uv* implies $v\prec _T u$, also implies $\tau _{T}(v)<\tau _{T}(u)$. Below we will make use of the fact that time maps are easily constructed for rooted trees:

#### Lemma 1

[4, Lemma 1] Given a tree *T* (planted or not), a time map $\tau _{T}$ for *T* satisfying $\tau _{T}(x)=\tau _0(x)$ with arbitrary choices of $\tau _0(x)$ for all $x\in L(T)$ can be constructed in linear time.

It is usually difficult and often impossible to obtain reliable, accurate “time stamps” $\tau _{T}(x)$ for evolutionary relevant events [12, 13]. Although the time map $\tau _{T}$ turns out to be a convenient formal tool, we will never need to make use of the absolute values of $\tau _{T}(x)$. Instead, we will only need *relative* timing information, i.e., it will be sufficient to know whether an event pre-dates, post-dates, or is concurrent with another one. This information is often much easier to extract [14, 15]. For the sake of concreteness, one may imagine that $\tau _0(x) = 0$ for all $x \in L(T)$, although this is not a requirement.

#### Definition 2

A *relaxed scenario* $\mathcal {S}=(T,S,\sigma ,\mu ,\tau _{T},\tau _{S})$ consists of a dated gene tree $(T,\tau _{T})$, a dated species tree $(S,\tau _{S})$, a leaf coloring $\sigma :L(T)\rightarrow M$ with $M\subseteq L(S)$, and a *reconciliation map* $\mu :V(T)\rightarrow V(S)\cup E(S)$ such that

(S0) $\mu (x)=0_S$ if and only if $x=0_T$.
(S1) $\mu (x)\in L(S)$ if and only if $x\in L(T)$ and, in particular, $\mu (x)=\sigma (x)$ in this case.
(S2) If $$\mu (x)\in V(S)$$, then $$\tau _{S}(\mu (x))=\tau _{T}(x)$$.
(S3) If $$\mu (x)=uv\in E(S)$$, then $$\tau _{S}(v)<\tau _{T}(x)<\tau _{S}(u)$$.

The axioms (S2) and (S3) specify *time consistency*. Note that we impose no (direct) restrictions on ancestrality relationships, hence the *relaxed* nature of our scenarios. In particular, for vertices $x, y \in V(T)$, it is possible that *x* is a descendant of *y*, but that $\mu (x)$ is *not* a descendant of $\mu (y)$. This may occur if $\mu (x)$ and $\mu (y)$ are incomparable because of the presence of horizontal gene transfers on the path from *y* to *x*. This contrasts with traditional reconciliation models that only support gene duplications and forbid this type of map. By minimizing the amount of constraints imposed on the model, we aim to characterize the broadest class of divergence time patterns that could be explained in some way. Conversely, this means that divergence times that cannot be explained by our scenarios can be deemed erroneous with more confidence, as they cannot even meet a relaxed set of requirements. In the later sections, however, we focus on more restrictive scenarios. As we shall see, relaxed scenarios allow “unobservable” transfers, for which the ancestral gene in the origin species has no direct extant descendants (in the sense that they were not transmitted by any transfer). We will study *restricted scenarios* in which such unobservable transfers are forbidden, and then later on we look at scenarios in which transfers are entirely forbidden. The scenarios considered in [16] as well as the H-trees [17] admit the assignment of unique event type (duplication, speciation, etc.) to a vertex *x* in the gene tree *T* depending on its reconciliation and the reconciliation of its children. This is not the case in relaxed scenarios. Here a vertex in *T* may simultaneously represent multiple event types. For example a “speciation” vertex with $\mu (x)\in V(S)$ may still have multiple direct descendants in the same lineage, hence sharing properties of of a duplication. We first consider a few simple properties of reconciliation maps. In fact, these are well-known properties for more restrictive definitions of reconciliation.

#### Lemma 2

Let $\mathcal {S}=(T,S,\sigma ,\mu ,\tau _{T},\tau _{S})$ be a relaxed scenario. If $v,w\in V(T)$ such that $v\preceq _T w$ and $\mu (v)=\mu (w)\in V^0(S)$, then $v=w$.

#### *Proof*

Set $U{:=}\mu (v)=\mu (w)\in V^0(S)$. Then $\tau _{T}(w)=\tau _{T}(v)=\tau _{S}(U)$. However, if $v\preceq _T w$ and $v\ne w$, i.e., $v\prec _T w$, then $\tau _{T}(v)<\tau _{T}(w)$ by Def. 1; a contradiction. $\square$

#### Lemma 3

If $\mathcal {S}=(T,S,\sigma ,\mu ,\tau _{T},\tau _{S})$ is a relaxed scenario then $x\preceq _T y$ implies $\mu (x)\not \succ _S \mu (y)$ for all $x,y\in V(T)$.

#### *Proof*

If $x=y$, then there is nothing to show. Otherwise, $x\prec _T y$ and Def. 1 implies that $\tau _{T}(x)<\tau _{T}(y)$. If $\mu (x)\in V(S)$ set $u{:=}\mu (x)$, otherwise let *u* be the lower delimiting vertex of the edge $\mu (x)\in E(S)$. Similarly, set $v{:=}\mu (y)$ if $\mu (y)\in V(S)$, otherwise choose *v* as the upper delimiting vertex of the edge $\mu (y)\in E(S)$. By time consistency, we have $\tau _{S}(u)\le \tau _{T}(x)$ and $\tau _{T}(y)\le \tau _{S}(v)$. Together with $\tau _{T}(x)<\tau _{T}(y)$, this yields $\tau _{S}(u)<\tau _{S}(v)$. Now assume, for contradiction, that $\mu (x)\succ _S\mu (y)$. One easily verifies that this implies $v\preceq _S u$ and thus $\tau _{S}(v)\le \tau _{S}(u)$; a contradiction. $\square$

#### Definition 3

The *HGT-labeling* of a relaxed scenario $\mathcal {S}$ is the map $\lambda :E(T)\rightarrow \{0,1\}$ such that $\lambda (uv)=1$ if and only if $\mu (u)$ and $\mu (v)$ are incomparable in *S*.

We call an edge $e\in E(T)$ with $\lambda (e)=1$ an *HGT edge*.

#### Definition 4

For a relaxed scenario $\mathcal {S}=(T,S,\sigma ,\mu ,\tau _{T},\tau _{S})$, we define the *equal-divergence-time* (*EDT*) graph $(G_{_{=}}(\mathcal {S}),\sigma )$, the *later-divergence-time* (*LDT*) graph $(G_{_{<}}(\mathcal {S}),\sigma )$ and the *prior-divergence-time* (*PDT*) graph $(G_{_{>}}(\mathcal {S}),\sigma )$ as follows: all graphs have as vertex set *L*(*T*) and are equipped with vertex coloring $\sigma :L(T)\rightarrow L(S)$. However, they differ in their edge sets defined as

1 $$\begin{aligned} E(G_{_{=}}(\mathcal {S}))&{:=}&\left\{ xy \mid x\ne y \text { and } \tau _{T}({{\,\textrm{lca}\,}}_T(x,y))=\tau _{S}({{\,\textrm{lca}\,}}_S(\sigma (x),\sigma (y))\right\} ,\nonumber \\ E(G_{_{<}}(\mathcal {S}))&{:=}&\left\{ xy \mid x\ne y \text { and } \tau _{T}({{\,\textrm{lca}\,}}_T(x,y)) < \tau _{S}({{\,\textrm{lca}\,}}_S(\sigma (x),\sigma (y))\right\} ,\nonumber \\ E(G_{_{>}}(\mathcal {S}))&{:=}&\left\{ xy \mid x\ne y \text { and } \tau _{T}({{\,\textrm{lca}\,}}_T(x,y)) > \tau _{S}({{\,\textrm{lca}\,}}_S(\sigma (x),\sigma (y))\right\} . \end{aligned}$$

Moreover, we write $\mathcal {G}(\mathcal {S})=(G_{_{<}}(\mathcal {S}),G_{_{=}}(\mathcal {S}),G_{_{>}}(\mathcal {S}),\sigma )$.

A vertex-colored graph $(G,\sigma )$ is an equal-divergence-time (EDT) graph, if there is a relaxed scenario $\mathcal {S}=(T,S,\sigma ,\mu ,\tau _{T},\tau _{S})$ such that $G = G_{_{=}}(\mathcal {S})$. In this case, we say that $\mathcal {S}$ *explains* $(G,\sigma )$. By construction, the edge sets of $G_{_{=}}(\mathcal {S})$, $G_{_{<}}(\mathcal {S})$, and $G_{_{>}}(\mathcal {S})$ are pairwise disjoint and their union is the edge set of the complete graph on *L*(*T*). This motivates the definition of the following tuple of vertex-colored graphs.

#### Definition 5

A *(colored) graph* 3-*partition*, denoted by $\mathcal {G}= (G_{_{<}}, G_{_{=}}, G_{_{>}}, \sigma )$, is an ordered tuple of three edge-disjoint graphs on the same vertex set *L* and with coloring $\sigma :L\rightarrow M$ such that  (i.e. every unordered pair of *L* is an edge of exactly one of the three graphs).

We say that $\mathcal {G}$ is *explained* by a scenario $\mathcal {S}$ if $G_{_{<}}=G_{_{<}}(\mathcal {S})$, $G_{_{=}}=G_{_{=}}(\mathcal {S})$, and $G_{_{>}}=G_{_{>}}(\mathcal {S})$.

**Fig. 2:**
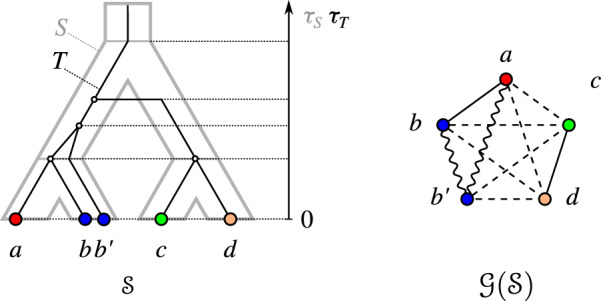
Left: a relaxed scenario $\mathcal {S}=(T,S,\sigma ,\mu ,\tau _{T},\tau _{S})$. The maps $\mu$ and $\sigma$ are shown implicitly by the embedding of *T* into *S* and the colors of the leaves of *T*, respectively. If a vertex *x* is drawn higher than a vertex *y*, this means that $\tau (y) <\tau '(x)$, $\tau ,\tau '\in \{\tau _{T},\tau _{S}\}$. In the remainder of the paper, we will omit drawing the time axis explicitly. Right: The graph 3-partition $\mathcal {G}(\mathcal {S})$ that is explained by $\mathcal {S}$. Throughout, the edges of the LDT graph $G_{_{<}}(\mathcal {S})$, EDT graph $G_{_{=}}(\mathcal {S})$, and PDT graph $G_{_{>}}(\mathcal {S})$ will always be drawn as dashed, solid straight, and wavy lines, respectively

An example for a graph 3-partition and a relaxed scenario that explains it is shown in Fig. 2.

The *restriction* $\mathcal {G}_{\vert L'}$ of a graph 3-partition $\mathcal {G}= (G_{_{<}}, G_{_{=}}, G_{_{>}}, \sigma )$ to a subset $L'\subseteq L$ of vertices is given by $(G_{_{<}}[L'], G_{_{=}}[L'], G_{_{>}}[L'], \sigma _{\vert L'})$.

#### Lemma 4

Let $\mathcal {S}=(T,S,\sigma ,\mu ,\tau _{T},\tau _{S})$ be a relaxed scenario. For all distinct vertices $x,y\in L(T)$ with $\sigma (x)=\sigma (y)$, it holds $xy\in E(G_{_{>}}(\mathcal {S}))$.

#### *Proof*

Since $x\ne y$, $u{:=}{{\,\textrm{lca}\,}}_T(x,y)$ is not a leaf. In particular, therefore, we have $\tau _{T}(x),\tau _{T}(y) < \tau _{T}(u)$ by the definition of time maps. Moreover, we have $\tau _{T}(x)=\tau _{S}(\sigma (x))$ by the definition of scenarios. If $\sigma (x)=\sigma (y)$, then ${{\,\textrm{lca}\,}}_S(\sigma (x),\sigma (y))=\sigma (x)$ is a leaf and thus $\tau _{S}({{\,\textrm{lca}\,}}_S(\sigma (x),\sigma (y)))=\tau _{S}(\sigma (x))=\tau _{T}(x)<\tau _{T}(u)$. Hence, $xy\in E(G_{_{>}}(\mathcal {S}))$. $\square$

The edge set of $G_{_{=}}(\mathcal {S})$, $G_{_{<}}(\mathcal {S})$, and $G_{_{>}}(\mathcal {S})$ are disjoint. Lemma 4 therefore implies

#### Corollary 1

Let $\mathcal {S}=(T,S,\sigma ,\mu ,\tau _{T},\tau _{S})$ be a relaxed scenario. If $xy\in E(G_{_{=}}(\mathcal {S}))$ or $xy\in E(G_{_{<}}(\mathcal {S}))$, then $\sigma (x)\ne \sigma (y)$, i.e., $G_{_{=}}(\mathcal {S})$ and $G_{_{<}}(\mathcal {S})$ are always *properly colored*.

Hence, neither the class of EDT graphs nor the class of LDT graphs is closed under complementation because the complements of $G_{_{=}}(\mathcal {S})$ and $G_{_{<}}(\mathcal {S})$ may contain edges between vertices with same color.

### Scenarios without HGT edges

In order to connect our discussion to the ample literature on DL-scenarios mentioned in the introduction, we briefly consider the case of HGT-free scenarios.

#### Lemma 5

If $\mathcal {S}=(T,S,\sigma ,\mu ,\tau _{T},\tau _{S})$ is a relaxed scenario without HGT-edges, then $x\preceq _T y$ implies $\mu (x)\preceq _S \mu (y)$ for all $x,y\in V(T)$.

#### *Proof*

Suppose $\mathcal {S}$ does not contain HGT-edges, i.e., $\mu (x)$ and $\mu (y)$ are comparable in *S* for all edges $yx\in E(T)$. Two vertices $x,y\in V(T)$ with $x\preceq _T y$ are either equal, implying $\mu (x)= \mu (y)$, or they lie on a directed path $v_1{:=}y, v_2, \dots v_k{:=}x$ with $k\ge 2$. If $yx\in E(T)$, then $x\prec _{T} y$ implies $\mu (x)\preceq _S \mu (y)$ due to Lemma 3. The vertices along a path in *T* therefore satisfy $\mu (x)\preceq _S\dots \preceq _S \mu (v_2)\preceq _S \mu (y)$. By transitivity of $\preceq _S$, we conclude that $x\prec _T y$ implies $\mu (x)\preceq _S\mu (y)$. $\square$

#### Lemma 6

If $\mathcal {S}$ is a relaxed scenario without HGT-edges, then any pair of distinct leaves $x,y\in L(T)$ satisfies ${{\,\textrm{lca}\,}}_S(\sigma (x),\sigma (y))\preceq _S \mu ({{\,\textrm{lca}\,}}_T(x,y))$ and $\tau _{S}({{\,\textrm{lca}\,}}_S(\sigma (x),\sigma (y))) \le \tau _{T}({{\,\textrm{lca}\,}}_T(x,y))$. In particular, we have ${{\,\textrm{lca}\,}}_S(\sigma (x),\sigma (y)) = \mu ({{\,\textrm{lca}\,}}_T(x,y))$ if and only if $\tau _{S}({{\,\textrm{lca}\,}}_S(\sigma (x),\sigma (y))) = \tau _{T}({{\,\textrm{lca}\,}}_T(x,y))$, i.e., $xy\in E(G_{_{=}})$.

#### *Proof*

Consider an arbitrary pair of distinct vertices *x*, *y* and $u{:=}{{\,\textrm{lca}\,}}_T(x,y)\in V(T)$. Then $x,y\preceq _T u$ and by Lemma 5 we have $\mu (x)\preceq _S \mu (u)$ and $\mu (y)\preceq _S \mu (u)$. Since *x* and *y* are leaves, we have $\sigma (x)=\mu (x)$ and $\sigma (y)=\mu (y)$. The definition of the ancestor order and the last common ancestor now imply ${{\,\textrm{lca}\,}}_S(\sigma (x),\sigma (y))\preceq _S \mu (u)$. If ${{\,\textrm{lca}\,}}_S(\sigma (x),\sigma (y)) = \mu (u)$, then time consistency implies $\tau _{S}({{\,\textrm{lca}\,}}_S(\sigma (x),\sigma (y))) = \tau _{T}(u)$. Conversely, suppose ${{\,\textrm{lca}\,}}_S(\sigma (x),\sigma (y))\prec _S \mu (u)$. If $\mu (u)$ is a vertex *v* of *S*, then we have $\tau _{S}({{\,\textrm{lca}\,}}_S(\sigma (x),\sigma (y))) < \tau _{S}(v)=\tau _{T}(u)$. If $\mu (u)$ is an edge *vw* of *S* (with $w\prec _S v$), then we have $\tau _{S}({{\,\textrm{lca}\,}}_S(\sigma (x),\sigma (y))) \le \tau _{S}(w)<\tau _{T}(u) < \tau _{S}(v)$. In either case we therefore obtain $\tau _{S}({{\,\textrm{lca}\,}}_S(\sigma (x),\sigma (y))) <\tau _{T}(u)$. $\square$

As an immediate consequence of Lemma 6, we recover [4, Cor. 6]:

#### Corollary 2

If $\mathcal {S}$ is a relaxed scenario without HGT-edges, then $G_{_{<}}(\mathcal {S})$ has no edges.

### Informative triples

If a graph 3-partition $\mathcal {G}=(G_{_{<}}, G_{_{=}}, G_{_{>}}, \sigma )$ is explained by some relaxed scenario ${\mathcal {S}=(T,S,\sigma ,\mu ,\tau _{T},\tau _{S})}$, several structural constraints on *T* and *S* can be deduced directly from $\mathcal {G}$. In particular, we show in this section that many subgraphs of $\mathcal {G}$ on three vertices enforce rooted triples that are either required or forbidden in *T* or *S*.

#### Lemma 7

Let $\mathcal {S}=(T,S,\sigma ,\mu ,\tau _{T},\tau _{S})$ be a relaxed scenario without HGT-edges, suppose $\sigma (x)$, $\sigma (y)$, and $\sigma (z)$ are pairwise distinct, the triple $xy\vert z$ is displayed by *T*, and $\mu ({{\,\textrm{lca}\,}}_T(x,z))={{\,\textrm{lca}\,}}_S(\sigma (x),\sigma (z))$. Then *S* displays $\sigma (x)\sigma (y)\vert \sigma (z)$.

#### *Proof*

By assumption ${{\,\textrm{lca}\,}}_T(x,y)\prec _T {{\,\textrm{lca}\,}}_T(x,z)={{\,\textrm{lca}\,}}_T(y,z)$. Lemma 5 implies $\mu ({{\,\textrm{lca}\,}}_T(x,y))\preceq _S\mu ({{\,\textrm{lca}\,}}_T(x,z))$ and Lemma 2 implies $\mu ({{\,\textrm{lca}\,}}_T(x,y))\ne \mu ({{\,\textrm{lca}\,}}_T(x,z))$ and thus $\mu ({{\,\textrm{lca}\,}}_T(x,y))\prec _T\mu ({{\,\textrm{lca}\,}}_T(x,z))$. Moreover, by Lemma 6 we have ${{\,\textrm{lca}\,}}_S(\sigma (x),\sigma (y))\preceq _S \mu ({{\,\textrm{lca}\,}}_T(x,y))$. We therefore conclude ${{\,\textrm{lca}\,}}_S(\sigma (x),\sigma (y))\preceq _S \mu ({{\,\textrm{lca}\,}}_T(x,y))\prec _S \mu ({{\,\textrm{lca}\,}}_T(x,z)) = {{\,\textrm{lca}\,}}_S(\sigma (x),\sigma (z))$. Therefore, *S* displays $\sigma (x)\sigma (y)\vert \sigma (z)$. $\square$

Lemma 7 defines the “informative species triples” [18–20] that play a key role for the characterization of feasible reconciliation maps in a slightly different setting.

We recall two results that link triples in *T* with the LDT graph:

#### Lemma 8

[4, Lemma 7] Let $\mathcal {S}=(T,S,\sigma ,\mu ,\tau _{T},\tau _{S})$ be a relaxed scenario with pairwise distinct leaves $x,y,z\in L(T)$. If $xy \in E(G_{_{<}}(\mathcal {S}))$ and $xz, yz \notin E(G_{_{<}}(\mathcal {S}))$, then *T* displays $xy\vert z$.

#### Lemma 9

[4, Lemma 6] Let $\mathcal {S}=(T,S,\sigma ,\mu ,\tau _{T},\tau _{S})$ be a relaxed scenario with leaves $x,y,z\in L(T)$ and pairwise distinct colors $X{:=}\sigma (x)$, $Y{:=}\sigma (y)$, and $Z{:=}\sigma (z)$. If $xz, yz \in E(G_{_{<}}(\mathcal {S}))$ and $xy \notin E(G_{_{<}}(\mathcal {S}))$, then *S* displays $XY\vert Z$.

For example, Lemma 9 applies to *b*, *c*, *d* in Fig. 2: $bc, bd \in E(G_{_{<}}(\mathcal {S}))$, $cd \notin E(G_{_{<}}(\mathcal {S}))$, and $\sigma (c) \sigma (d) \vert \sigma (b)$ is a triple of the species tree. We next show a statement similar to Lemma 8 for the corresponding PDT $G_{_{>}}(\mathcal {S})$:

#### Lemma 10

Let $\mathcal {S}=(T,S,\sigma ,\mu ,\tau _{T},\tau _{S})$ be a relaxed scenario with pairwise distinct leaves $x,y,z\in L(T)$. If $xz, yz \in E(G_{_{>}}(\mathcal {S}))$ and $xy \notin E(G_{_{>}}(\mathcal {S}))$, then *T* displays $xy\vert z$.

#### *Proof*

Suppose $xz, yz \in E(G_{_{>}}(\mathcal {S}))$ and $xy \notin E(G_{_{>}}(\mathcal {S}))$. Put $X{:=}\sigma (x)$, $Y{:=}\sigma (y)$, and $Z{:=}\sigma (z)$ and observe that $X\ne Y$ by Cor. 1. Assume for contradiction that $xy\vert z$ is not displayed by *T*. Hence, the tree *T* displays either $xz\vert y$ or $yz\vert x$ or ${{\,\textrm{lca}\,}}_T(x,y)={{\,\textrm{lca}\,}}_T(x,z)={{\,\textrm{lca}\,}}_T(y,z)$. One easily verifies that, in all three cases, it holds ${{\,\textrm{lca}\,}}_T(x,y)\succeq _{T}{{\,\textrm{lca}\,}}_T(x,z)$ and ${{\,\textrm{lca}\,}}_T(x,y)\succeq _{T}{{\,\textrm{lca}\,}}_T(y,z)$. This together with the assumption that $xz, yz \in E(G_{_{>}}(\mathcal {S}))$ and $xy \notin E(G_{_{>}}(\mathcal {S}))$ and time consistency implies

$$\begin{aligned}&\tau _{S}({{\,\textrm{lca}\,}}_S(X,Y))\ge \tau _{T}({{\,\textrm{lca}\,}}_T(x,y)) \ge \tau _{T}({{\,\textrm{lca}\,}}_T(x,z))> \tau _{S}({{\,\textrm{lca}\,}}_S(X,Z)) \text { and} \\&\tau _{S}({{\,\textrm{lca}\,}}_S(X,Y))\ge \tau _{T}({{\,\textrm{lca}\,}}_T(x,y)) \ge \tau _{T}({{\,\textrm{lca}\,}}_T(y,z)) > \tau _{S}({{\,\textrm{lca}\,}}_S(Y,Z)). \end{aligned}$$

In particular, this implies that $Y\ne Z$ and $X\ne Z$, resp., and thus *X*, *Y*, and *Z* are pairwise distinct. Since ${{\,\textrm{lca}\,}}_S(X,Y)$ and ${{\,\textrm{lca}\,}}_S(X,Z)$ are both ancestors of *X*, they are comparable in *S*. Together with $\tau _{S}({{\,\textrm{lca}\,}}_S(X,Y)) > \tau _{S}({{\,\textrm{lca}\,}}_S(X,Z))$ and the definition of time maps, this implies ${{\,\textrm{lca}\,}}_S(X,Y) \succ _S {{\,\textrm{lca}\,}}_S(X,Z)$. Thus, *S* displays the triple $XZ\vert Y$. By similar arguments, we obtain that *S* also displays the triple $YZ\vert X$; a contradiction. Hence, *T* must display $xy\vert z$. $\square$

Again using Fig. 2 as an example, one can check that *T* must display $ab|b'$ because of Lemma 10. Let us now consider the EDT graph:

#### Lemma 11

Let $\mathcal {S}=(T,S,\sigma ,\mu ,\tau _{T},\tau _{S})$ be a relaxed scenario with pairwise distinct leaves $x,y,z\in L(T)$ and suppose that $xz, yz \in E(G_{_{=}}(\mathcal {S}))$. If $xy \notin E(G_{_{=}}(\mathcal {S}))$, then *T* displays neither $xz\vert y$ nor $yz\vert x$. In particular, if $xy \in E(G_{_{<}}(\mathcal {S}))$, then *T* displays $xy\vert z$.

#### *Proof*

Suppose that $xz, yz \in E(G_{_{=}}(\mathcal {S}))$ and $xy \notin E(G_{_{=}}(\mathcal {S}))$. Recall that $G_{_{=}}(\mathcal {S})$, $G_{_{<}}(\mathcal {S})$, and $G_{_{>}}(\mathcal {S})$ are pairwise edge-disjoint. Put $X{:=}\sigma (x)$, $Y{:=}\sigma (y)$, and $Z{:=}\sigma (z)$ and observe that $X\ne Z$ and $Y\ne Z$ by Cor. 1. If $xy\in E(G_{_{<}}(\mathcal {S}))$, then Lemma 8 implies that *T* displays $xy\vert z$ and thus, none of $xz\vert y$ or $yz\vert x$. Now suppose $xy\in E(G_{_{>}}(\mathcal {S}))$ and assume, for contradiction that *T* displays $xz\vert y$ and thus ${{\,\textrm{lca}\,}}_T(x,z)\prec _T {{\,\textrm{lca}\,}}_T(x,y)={{\,\textrm{lca}\,}}_T(y,z)$. By assumption and time consistency, this implies $\tau _{S}({{\,\textrm{lca}\,}}_S(X,Z))=\tau _{T}({{\,\textrm{lca}\,}}_T(x,z)) < \tau _{T}({{\,\textrm{lca}\,}}_T(y,z)) = \tau _{S}({{\,\textrm{lca}\,}}_S(Y,Z))$. The latter implies that $X\ne Y$ and thus *X*, *Y*, and *Z* are pairwise distinct. Since ${{\,\textrm{lca}\,}}_S(X,Z)$ and ${{\,\textrm{lca}\,}}_S(Y,Z)$ are both ancestors of *Z*, they are comparable in *S*. Together with $\tau _{S}({{\,\textrm{lca}\,}}_S(X,Z)) < \tau _{S}({{\,\textrm{lca}\,}}_S(Y,Z))$ and the definition of time maps, this implies ${{\,\textrm{lca}\,}}_S(X,Z) \prec _S {{\,\textrm{lca}\,}}_S(Y,Z)$. Thus, *S* displays the triple $XZ\vert Y$. Therefore, we have ${{\,\textrm{lca}\,}}_S(X,Y)={{\,\textrm{lca}\,}}_S(Y,Z)$. In summary, we obtain $\tau _{S}({{\,\textrm{lca}\,}}_S(X,Y)) = \tau _{S}({{\,\textrm{lca}\,}}_S(Y,Z)) = \tau _{T}({{\,\textrm{lca}\,}}_T(y,z)) = \tau _{T}({{\,\textrm{lca}\,}}_T(x,y))$; a contradiction to $xy\in E(G_{_{>}}(\mathcal {S}))$. Hence, *T* does not display $xz\vert y$. For similar reasons, *T* does not display $yz\vert x$, which concludes the proof. $\square$

The case that $xz, yz \in E(G_{_{=}}(\mathcal {S}))$, $xy \in E(G_{_{>}}(\mathcal {S}))$ and $xy\vert z$ is not displayed by *T* is not covered by Lemma 11. To see that this situation is possible, consider the trees $S=((X,Y),Z)$ and $T=(x,y,z)$ with $\sigma (x)=X$, $\sigma (y)=Y$ and $\sigma (z)=Z$. Now choose $\mu$ such that $\mu (\rho _T) = \rho _S$. One easily verifies that $xz, yz \in E(G_{_{=}}(\mathcal {S}))$ and $xy \in E(G_{_{>}}(\mathcal {S}))$ while *T* by construction does not displayed $xy\vert z$.

#### Lemma 12

Let $\mathcal {S}=(T,S,\sigma ,\mu ,\tau _{T},\tau _{S})$ be a relaxed scenario with leaves $x,y,z\in L(T)$ and pairwise distinct colors $X{:=}\sigma (x)$, $Y{:=}\sigma (y)$, and $Z{:=}\sigma (z)$. Suppose that $xz, yz \in E(G_{_{=}}(\mathcal {S}))$. If $xy \notin E(G_{_{=}}(\mathcal {S}))$, then *S* displays neither $XZ\vert Y$ nor $YZ\vert X$. If, in particular, $xy \in E(G_{_{>}}(\mathcal {S}))$ then *S* displays $XY\vert Z$.

#### *Proof*

Suppose that $xz, yz \in E(G_{_{=}}(\mathcal {S}))$ and $xy \notin E(G_{_{=}}(\mathcal {S}))$. By Lemma 11, *T* does not display $xz\vert y$ or $yz\vert x$. Suppose for contradiction that *S* displays $XZ\vert Y$, i.e., ${{\,\textrm{lca}\,}}_S(X,Z)\prec _S {{\,\textrm{lca}\,}}_S(Y,Z)$. This together with the assumption that $xz, yz \in E(G_{_{=}}(\mathcal {S}))$ and time consistency implies $\tau _{T}({{\,\textrm{lca}\,}}_T(x,z)) = \tau _{S}({{\,\textrm{lca}\,}}_S(X,Z)) < \tau _{S}({{\,\textrm{lca}\,}}_S(Y,Z)) = \tau _{T}({{\,\textrm{lca}\,}}_T(y,z))$. Since ${{\,\textrm{lca}\,}}_T(x,z)$ and ${{\,\textrm{lca}\,}}_T(y,z)$ are both ancestors of *z*, they must be comparable. This together with $\tau _{T}({{\,\textrm{lca}\,}}_T(x,z)) < \tau _{T}({{\,\textrm{lca}\,}}_T(y,z))$ yields ${{\,\textrm{lca}\,}}_T(x,z)\prec _T {{\,\textrm{lca}\,}}_T(y,z)$ and thus *T* displays $xz\vert y$; a contradiction. Therefore, *S* does not display $XZ\vert Y$. For similar reasons, $YZ\vert X$ is not displayed.

Now assume in addition that $xy \in E(G_{_{>}}(\mathcal {S}))$. Since *T* does not display $xz\vert y$ and ${{\,\textrm{lca}\,}}_T(x,y)$ and ${{\,\textrm{lca}\,}}_T(x,z)$ are both ancestors of *x* and thus comparable, we have ${{\,\textrm{lca}\,}}(x,y)\preceq _T{{\,\textrm{lca}\,}}_T(x,z)$. Now this together with time consistency, $xy \in E(G_{_{>}}(\mathcal {S}))$, and $xz \in E(G_{_{=}}(\mathcal {S}))$ yields $\tau _{S}({{\,\textrm{lca}\,}}_S(X,Y)) < \tau _{T}({{\,\textrm{lca}\,}}_T(x,y)) \le \tau _{T}({{\,\textrm{lca}\,}}_T(x,z)) = \tau _{S}({{\,\textrm{lca}\,}}_S(X,Z))$. Since ${{\,\textrm{lca}\,}}_S(X,Y)$ and ${{\,\textrm{lca}\,}}_S(X,Z)$ are both ancestors of *X*, they are comparable in *S*. Together with $\tau _{S}({{\,\textrm{lca}\,}}_S(X,Y)) < \tau _{S}({{\,\textrm{lca}\,}}_S(X,Z))$ and the definition of time maps, this implies ${{\,\textrm{lca}\,}}_S(X,Y) \prec _S {{\,\textrm{lca}\,}}_S(X,Z)$. Thus, *S* displays the triple $XY\vert Z$. $\square$

Finally, we consider the species triples implied by the PDT graph. The following result in particular generalizes the last statement in Lemma 12 above.

#### Lemma 13

Let $\mathcal {S}=(T,S,\sigma ,\mu ,\tau _{T},\tau _{S})$ be a relaxed scenario with leaves $x,y,z\in L(T)$ and pairwise distinct colors $X{:=}\sigma (x)$, $Y{:=}\sigma (y)$, and $Z{:=}\sigma (z)$. If $xy \in E(G_{_{>}}(\mathcal {S}))$ and $xz, yz \notin E(G_{_{>}}(\mathcal {S}))$, then *S* displays $XY\vert Z$.

#### *Proof*

Recall that by construction $G_{_{<}}(\mathcal {S})$, $G_{_{=}}(\mathcal {S})$, and $G_{_{>}}(\mathcal {S})$ are edge-disjoint. If $xz, yz \in E(G_{_{<}}(\mathcal {S}))$ or $xz, yz \in E(G_{_{=}}(\mathcal {S}))$, the statement follows immediately from Lemma 9 and 12, respectively. Now consider the case that $xz \in E(G_{_{<}}(\mathcal {S}))$ and $yz \in E(G_{_{=}}(\mathcal {S}))$. Hence, we have $\tau _{T}({{\,\textrm{lca}\,}}_T(x,y)) > \tau _{S}({{\,\textrm{lca}\,}}_S(X,Y))$ and $\tau _{T}({{\,\textrm{lca}\,}}_T(y,z)) = \tau _{S}({{\,\textrm{lca}\,}}_S(Y,Z))$. Moreover, *T* displays $xz\vert y$ by Lemma 8 and thus ${{\,\textrm{lca}\,}}_T(x,y)={{\,\textrm{lca}\,}}_T(y,z)$. To summarize, we have $\tau _{S}({{\,\textrm{lca}\,}}_S(Y,Z)) = \tau _{T}({{\,\textrm{lca}\,}}_T(y,z)) = \tau _{T}({{\,\textrm{lca}\,}}_T(x,y)) > \tau _{S}({{\,\textrm{lca}\,}}_S(X,Y))$. Since ${{\,\textrm{lca}\,}}_S(X,Y)$ and ${{\,\textrm{lca}\,}}_S(Y,Z)$ are both ancestors of *Y*, they are comparable in *S*. Together with $\tau _{S}({{\,\textrm{lca}\,}}_S(Y,Z)) > \tau _{S}({{\,\textrm{lca}\,}}_S(X,Y))$ and the definition of time maps, this implies ${{\,\textrm{lca}\,}}_S(X,Y) \prec _S {{\,\textrm{lca}\,}}_S(Y,Z)$. Thus, *S* displays the triple $XY\vert Z$. One proceeds similarly if $yz \in E(G_{_{<}}(\mathcal {S}))$ and $xz \in E(G_{_{=}}(\mathcal {S}))$. $\square$

See $a, b', c$ in Fig. 2, which enforce $\sigma (a) \sigma (b') \vert \sigma (c)$ in the species tree by Lemma 13. With the facts that we have gathered, we can now define our set of required and forbidden triples.

#### Definition 6

Let $\mathcal {G}=(G_{_{<}}, G_{_{=}}, G_{_{>}}, \sigma )$ be a tuple of three graphs on the same vertex set *L* and with vertex coloring $\sigma$.

The set of *informative triples on* *L*, denoted by $\mathcal {R}_T(\mathcal {G})$, contains a triple $xy\vert z$ if $x,y,z\in L$ and one of the following conditions holds

- $$xy \in E(G_{_{<}})$$ and $$xz, yz\notin E(G_{_{<}})$$,
- $$xz, yz \in E(G_{_{>}})$$ and $$xy \notin E(G_{_{>}})$$.

The set of *forbidden triples on* *L*, denoted by $\mathcal {F}_T(\mathcal {G})$, contains a triple $xz\vert y$ (and by symmetry also $yz\vert x$) if $x,y,z\in L$ and $xz, yz \in E(G_{_{=}})$ and $xy \notin E(G_{_{=}})$.

The set of *informative triples on* $\sigma (L)$, denoted by $\mathcal {R}_S(\mathcal {G})$, contains a triple $XY\vert Z$ if there are $x,y,z\in L$ with pairwise distinct colors $X{:=}\sigma (x)$, $Y{:=}\sigma (y)$, and $Z{:=}\sigma (z)$ and one of the following conditions holds

(a’) $$xz, yz \in E(G_{_{<}})$$ and $$xy \notin E(G_{_{<}})$$,
(b’) $$xy \in E(G_{_{>}})$$ and $$xz, yz \notin E(G_{_{>}})$$.

The set of *forbidden triples on* *L*(*S*), denoted by $\mathcal {F}_S(\mathcal {G})$, contains a triple $XZ\vert Y$ (and by symmetry also $YZ\vert X$) if there are $x,y,z\in L$ with pairwise distinct colors $X{:=}\sigma (x)$, $Y{:=}\sigma (y)$, $Z{:=}\sigma (z)$, and $xz, yz \in E(G_{_{=}})$ and $xy \notin E(G_{_{=}})$.

The notation $\mathcal {R}_T$, $\mathcal {F}_T$, $\mathcal {R}_S$, and $\mathcal {F}_S$ in Definition 6 is motivated by Proposition 2 below, which shows that the triples on *L* and *L*(*S*), resp., provide information of the gene tree *T* and species tree *S* explaining $\mathcal {G}$, provided such trees exists. Summarizing Lemmas 8 to 13, we obtain:

#### Proposition 2

Let $\mathcal {S}=(T,S,\sigma ,\mu ,\tau _{T},\tau _{S})$ be a relaxed scenario and $\mathcal {G}=(G_{_{<}}(\mathcal {S}), G_{_{=}}(\mathcal {S}), G_{_{>}}(\mathcal {S}), \sigma )$. Then *T* agrees with $(\mathcal {R}_T(\mathcal {G}), \mathcal {F}_T(\mathcal {G}))$ and *S* agrees with $(\mathcal {R}_S(\mathcal {G}), \mathcal {F}_S(\mathcal {G}))$.

## The cograph structure

Cographs naturally appear as graph structures associated with vertex-labeled trees and more generally in the context of binary relations associated with reconciliations of gene trees and species trees. For example, orthology graphs in scenarios without horizontal gene transfer are cographs [21]. As we shall see below, both $G_{_{<}}(\mathcal {S})$ and $G_{_{>}}(\mathcal {S})$ are cographs for all relaxed scenarios $\mathcal {S}$. In contrast, $G_{_{=}}(\mathcal {S})$ is a cograph only under some additional constraints. It is, however, always a so-called perfect graph.

### Lemma 14

[4, Lemma 8] Let $\mathcal {S}=(T,S,\sigma ,\mu ,\tau _{T},\tau _{S})$ be a relaxed scenario. Then $G_{_{<}}(\mathcal {S})$ is a cograph.

It may not come as a surprise, therefore, that an analogous result holds for $G_{_{>}}(\mathcal {S})$:

### Lemma 15

Let $\mathcal {S}=(T,S,\sigma ,\mu ,\tau _{T},\tau _{S})$ be a relaxed scenario. Then $G_{_{>}}(\mathcal {S})$ is a cograph.

### *Proof*

Set $A{:=}\sigma (a)$, $B{:=}\sigma (b)$, $C{:=}\sigma (c)$, and $D{:=}\sigma (d)$. Suppose for contradiction that $G_{_{>}}(\mathcal {S})$ is not a cograph, i.e., it contains an induced $P_4$ $a-b-c-d$. By Prop. 2, *T* displays the informative triples $ac\vert b$ and $bd\vert c$. Hence, $T_{\vert abcd}=((a,c),(b,d))$ and, therefore, ${{\,\textrm{lca}\,}}_T(a,d) = {{\,\textrm{lca}\,}}_T(b,c)$. Moreover, by Cor. 1, we know that $A\ne C$, $A\ne D$, and $B \ne D$. Therefore, we have to consider the cases (i) $\vert \{A,B,C,D\}\vert =4$; (ii) $\vert \{A,B,C,D\}\vert =2$; (iii) $\vert \{A,B,C,D\}\vert =3$ and $A=B$, *C*, and *D* are pairwise distinct; (iv) $\vert \{A,B,C,D\}\vert =3$ and *A*, *B*, and $C=D$ are pairwise distinct; and (v) $\vert \{A,B,C,D\}\vert =3$ and *A*, $B=C$, and *D* are pairwise distinct.

In Case (i), *A*, *B*, *C*, and *D* are pairwise distinct. By Prop. 2, *S* displays the informative triples $AB\vert D$ and $CD\vert A$ (see Definition 6.b’). Thus, $S_{\vert ABCD} = ((A,B),(C,D))$ and we have ${{\,\textrm{lca}\,}}_S(B,C) = {{\,\textrm{lca}\,}}_S(A,D)$. In Case (ii), we must have $A=B$ and $C=D$. Thus, we again obtain ${{\,\textrm{lca}\,}}_S(B,C)={{\,\textrm{lca}\,}}_S(A,D)$.

In Case (iii), Prop. 2 implies that *S* displays the informative triple $CD\vert A (=CD\vert B)$. Thus, we have ${{\,\textrm{lca}\,}}_S(B,C) = {{\,\textrm{lca}\,}}_S(A,D)$. In Case (iv), Prop. 2 implies that *S* displays the informative triple $AB\vert D (=AB\vert C)$. Thus, we have ${{\,\textrm{lca}\,}}_S(B,C) = {{\,\textrm{lca}\,}}_S(A,D)$. In Case (v), Prop. 2 implies that *S* displays the informative triples $AB\vert D$ and $CD\vert A (=BD\vert A)$. Since *S* cannot displays both of these triples, Case (v) can be immediately excluded.

In Cases (i)–(iv), we have ${{\,\textrm{lca}\,}}_T(a,d) = {{\,\textrm{lca}\,}}_T(b,c)$ and ${{\,\textrm{lca}\,}}_S(B,C) = {{\,\textrm{lca}\,}}_S(A,D)$. Together with $bc\in E(G_{_{>}}(\mathcal {S}))$, it follows $\tau _{T}({{\,\textrm{lca}\,}}_T(a,d)) = \tau _{T}({{\,\textrm{lca}\,}}_T(b,c)) > \tau _{S}({{\,\textrm{lca}\,}}_S(B,C)) = \tau _{S}({{\,\textrm{lca}\,}}_S(A,D))$; a contradiction to $ad\notin E(G_{_{>}}(\mathcal {S}))$.

In summary, $G_{_{>}}(\mathcal {S})$ does not contain an induced $P_4$ and thus it is a cograph. $\square$

**Fig. 3:**
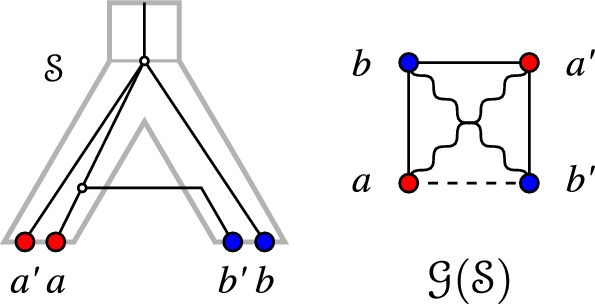
$(G_{_{=}}(\mathcal {S}),\sigma )$ can contain a 2-colored $P_4 = a-b-a'-b'$. However, due to Cor. 3, *T* cannot be a binary tree in this case

Lemmas 14 and 15 naturally suggest to ask whether an analogous result holds for $G_{_{=}}(\mathcal {S})$, i.e., whether the EDT graph is always a cograph. If this is the case, $\{G_{_{<}}(\mathcal {S}),G_{_{=}}(\mathcal {S}),G_{_{>}}(\mathcal {S})\}$ form a “cograph 3-partition” in the sense of [22, 23]. As illustrated in Fig. 3, this is not the case in general. Therefore, we investigate in the following conditions under which $G_{_{=}}(\mathcal {S})$ may or may not be a cograph and their implications for the underlying tree structure.

### Lemma 16

Let $\mathcal {S}=(T,S,\sigma ,\mu ,\tau _{T},\tau _{S})$ be a relaxed scenario. If $(G_{_{=}}(\mathcal {S}),\sigma )$ contains an induced $P_4$ $a-b-c-d$ on two colors, then $T_{\vert {abcd}} = ((a,d),b,c)$.

### *Proof*

By assumption and by Cor. 1, $A{:=}\sigma (a) = \sigma (c)$, $B{:=}\sigma (b) = \sigma (d)$, and $A\ne B$. Therefore, and since $ab, bc, cd\in E(G_{_{=}}(\mathcal {S}))$, we have $\tau _{T}({{\,\textrm{lca}\,}}_T(a,b)) = \tau _{T}({{\,\textrm{lca}\,}}_T(b,c))=\tau _{T}({{\,\textrm{lca}\,}}_T(c,d))=\tau _{S}({{\,\textrm{lca}\,}}_S(A,B))$. Def. 1 together with $\tau _{T}({{\,\textrm{lca}\,}}_T(a,b)) = \tau _{T}({{\,\textrm{lca}\,}}_T(b,c))$ implies that we can have neither ${{\,\textrm{lca}\,}}_T(a,b) \prec _T {{\,\textrm{lca}\,}}_T(b,c)$ nor ${{\,\textrm{lca}\,}}_T(b,c) \prec _T {{\,\textrm{lca}\,}}_T(a,b)$. Since ${{\,\textrm{lca}\,}}_T(a,b)$ and ${{\,\textrm{lca}\,}}_T(b,c)$ are both ancestors of *b* and thus comparable in *T*, we conclude ${{\,\textrm{lca}\,}}_T(a,b) ={{\,\textrm{lca}\,}}_T(b,c)$. Similarly, we obtain ${{\,\textrm{lca}\,}}_T(b,c) ={{\,\textrm{lca}\,}}_T(c,d)$. Moreover, since $ad\notin E(G_{_{=}}(\mathcal {S}))$, we have $\tau _{T}({{\,\textrm{lca}\,}}_T(a,d))\ne \tau _{S}({{\,\textrm{lca}\,}}_S(A,B))=\tau _{T}({{\,\textrm{lca}\,}}_T(a,b))$ and thus ${{\,\textrm{lca}\,}}_T(a,d) \ne {{\,\textrm{lca}\,}}_T(a,b)$, which implies that *T* displays one of the triples $t_1 = ab\vert d$ or $t'_1=ad\vert b$. By similar arguments, *T* displays one of the triples $t_2 = cd\vert a$ or $t'_2=ad\vert c$. We next examine the possible combination of these triples.

If *T* displays $t_1$ and $t_2$, then $T_{\vert abcd} = ((a,b),(c,d))$, in which case ${{\,\textrm{lca}\,}}_T(a,b) \ne {{\,\textrm{lca}\,}}_T(b,c)$; a contradiction. If *T* displays $t_1$ and $t'_2$, then $T_{\vert abcd} = (((a,b),d),c)$. Again ${{\,\textrm{lca}\,}}_T(a,b) \ne {{\,\textrm{lca}\,}}_T(b,c)$; again a contradiction. If *T* displays $t'_1$ and $t_2$, then $T_{\vert abcd} = (((c,d),a),b)$. Hence ${{\,\textrm{lca}\,}}_T(a,b) \ne {{\,\textrm{lca}\,}}_T(c,d)$; a contradiction. If *T* displays $t'_1$ and $t'_2$, then $T_{\vert abcd}$ is either of the form (((*a*, *d*), *c*), *b*), (((*a*, *d*), *b*), *c*), ((*a*, *d*), *b*, *c*), or ((*a*, *d*), (*b*, *c*)). For the first two cases, we obtain ${{\,\textrm{lca}\,}}_T(a,b)\ne {{\,\textrm{lca}\,}}_T(c,d)$, while for the latter case we obtain ${{\,\textrm{lca}\,}}_T(b,c)\ne {{\,\textrm{lca}\,}}_T(c,d)$. Thus we reach a contradiction in all three cases, leaving $T_{\vert abcd} = ((a,d),b,c)$ as the only possibility. $\square$

Note that the tree $T_{\vert {abcd}} = ((a,d),b,c)$ in Lemma 16 is displayed by *T* but not binary. Hence, we obtain

### Corollary 3

Let $\mathcal {S}=(T,S,\sigma ,\mu ,\tau _{T},\tau _{S})$ be a relaxed scenario. If $(G_{_{=}}(\mathcal {S}),\sigma )$ contains a 2-colored $P_4$, then *T* is not a binary tree.

### Lemma 17

Let $\mathcal {S}=(T,S,\sigma ,\mu ,\tau _{T},\tau _{S})$ be a relaxed scenario. If $(G_{_{=}}(\mathcal {S}),\sigma )$ contains an induced $P_4$ $a-b-c-a'$ on three distinct colors with $A=\sigma (a)=\sigma (a')$, $B=\sigma (b)$, and $C=\sigma (c)$, then $S_{\vert ABC} = (A,B,C)$. In particular, *S* is not a binary tree. Moreover, we have $T_{\vert {abca'}} = ((a,c),(b,a'))$.

### *Proof*

By assumption $P_3 = a-b-c$ is an induced path. Lemma 12 thus imply that *S* does not display $AB\vert C$ and $BC\vert A$. Similarly, the induced $P_3 = b-c-a'$ implies that *S* does not display $BC\vert A$ and $AC\vert B$. This leaves $S_{\vert ABC} = (A,B,C)$ as the only possibility. By Lemma 12, we immediately see that $ac, ba'\in G_{_{<}}(\mathcal {S})$ since otherwise *S* would display $AC\vert B$ or $AB\vert C$. This, together with Lemma 8 and $ab,bc,ca' \notin G_{_{<}}(\mathcal {S})$, implies that *T* displays $ac\vert b$ and $ba'\vert c$ and, therefore, $T_{\vert {abca'}} = ((a,c),(b,a'))$. $\square$

**Fig. 4:**
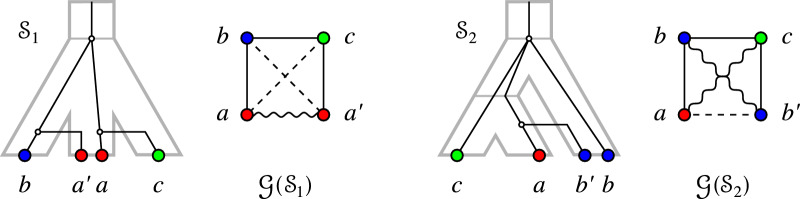
Left: $(G_{_{=}}(\mathcal {S}_1),\sigma )$ contains an induced path $P_4 = a-b-c-a'$ on three colors with $\sigma (a)= \sigma (a')$ as in Lemma 17. Right: $(G_{_{=}}(\mathcal {S}_2),\sigma ')$ contains an induced path $P_4 = a-b-c-b'$ on three colors with $\sigma '(b)= \sigma '(b')$ as in Lemma 18

### Lemma 18

Let $\mathcal {S}=(T,S,\sigma ,\mu ,\tau _{T},\tau _{S})$ be a relaxed scenario. If $E(G_{_{=}}(\mathcal {S}))$ contains an induced $P_4$ $a-b-c-b'$ on three distinct colors with $A=\sigma (a)$, $B=\sigma (b)=\sigma (b')$ and $C=\sigma (c)$, then $S_{\vert ABC} = ((A,C),B)$ and $T_{\vert abcb'}=((a,b'),b,c)$.

### *Proof*

Suppose that $G_{_{=}}(\mathcal {S})$ contains an induced $P_4$ $a-b-c-b'$ on three distinct colors $A=\sigma (a)$, $B=\sigma (b)=\sigma (b')$, and $C=\sigma (c)=C$. By Lemma 11, *T* displays neither $bc\vert b'$ nor $b'c\vert b$. Hence, we have to consider two cases: (1) $T_{\vert bcb'}=(b,c,b')$, or (2) $T_{\vert bcb'}=bb'\vert c$. By similar arguments, we have either (I) $T_{\vert abc}=(a,b,c)$ or (II) $T_{\vert abc}=ac\vert b$. We proceed by combining these alternatives:

Case (1,I) yields (i) $T_{\vert abcb'}=(a,b,c,b')$ or (ii) $T_{\vert abcb'}=((a,b'),b,c)$, Case (1,II) yields $T_{\vert abcb'}=((a,c),b,b')$, Case (2,I) yields $T_{\vert abcb'}=((b,b'),a,c)$, and Case (2,II) yields $T_{\vert abcb'} = ((b,b'),(a,c))$. In all cases except Case (1,I,ii), we have ${{\,\textrm{lca}\,}}_T(a,b)={{\,\textrm{lca}\,}}_T(a,b')$ and $ab\in E(G_{_{=}}(\mathcal {S}))$ thus implies $\tau _{S}({{\,\textrm{lca}\,}}_S(A,B)) = \tau _{T}({{\,\textrm{lca}\,}}_T(a,b))=\tau _{T}({{\,\textrm{lca}\,}}_T(a,b'))$ and $ab'\in E(G_{_{=}}(\mathcal {S}))$; a contradiction. This leaves Case (1,I,ii), $T_{\vert abcb'}=((a,b'),b,c)$, as the only possibility. Lemma 12 together with $ab,bc\in E(G_{_{=}}(\mathcal {S}))$ and $ac\notin E(G_{_{=}}(\mathcal {S}))$ implies that either $S_{\vert ABC} = (A,B,C)$ or $S_{\vert ABC} = AC\vert B$. In the first case, we have ${{\,\textrm{lca}\,}}_S(A,C)={{\,\textrm{lca}\,}}_S(B,C)$. Together with $T_{\vert abcb'}=((a,b'),b,c)$ (and thus ${{\,\textrm{lca}\,}}_T(b,c)={{\,\textrm{lca}\,}}_T(a,c)$) and $bc\in E(G_{_{=}}(\mathcal {S}))$, we obtain $\tau _{S}({{\,\textrm{lca}\,}}_S(A,C)) = \tau _{S}({{\,\textrm{lca}\,}}_S(B,C)) = \tau _{T}({{\,\textrm{lca}\,}}_T(b,c)) = \tau _{T}({{\,\textrm{lca}\,}}_T(a,c))$. Therefore, we must have $ac\in E(G_{_{=}})$; a contradiction. In summary, therefore, we have $S_{\vert ABC} = ((A,C),B)$ and $T_{\vert abcb'}=((a,b'),b,c)$. $\square$

Figure 4 shows two examples of scenarios that realize EDT graphs containing $P_4$s on three colors as described in Lemma 17 and Lemma 18, respectively.

Instead of considering the three graphs $G_{_{<}}$, $G_{_{=}}$, and $G_{_{>}}$ in isolation, we can alternatively think of a graph 3-partition $\mathcal {G}=\{G_{_{<}},G_{_{=}},G_{_{>}},\sigma \}$ as a complete graph $K_n$ whose edges are colored with three different colors depending on whether they are contained in $E(G_{_{<}})$, $E(G_{_{=}})$, or $E(G_{_{>}})$. This links our results to the literature on edge-colored graphs. Complete edge-colored permutation graphs are characterized [24] as the edge-partitions of $K_n$ such that (i) each color class induces a permutation graph in the usual sense [25], and (ii) the edge coloring is a Gallai coloring, i.e., it contains no “rainbow triangle” with three distinct colors. While every cograph is also a permutation graph [25], rainbow triangles may appear in the edge-coloring defined by $\{G_{_{<}},G_{_{=}},G_{_{>}}\}$ that is explained by a relaxed scenario. In fact, induced $P_4$s in $G_{_{=}}$ are always associated with rainbow triangles.

### Lemma 19

Let $\mathcal {S}=(T,S,\sigma ,\mu ,\tau _{T},\tau _{S})$ be a relaxed scenario. If $G_{_{=}}(\mathcal {S})$ contains an induced $P_4 = a-b-c-d$, then either $ad\in E(G_{_{<}}(\mathcal {S}))$ and $ac,bd \in E(G_{_{>}}(\mathcal {S}))$ or $ad\in E(G_{_{>}}(\mathcal {S}))$ and $ac,bd \in E(G_{_{<}}(\mathcal {S}))$. In either case, both $\{a,b,d\}$ and $\{a,c,d\}$ are rainbow triangles.

### *Proof*

Suppose $G_{_{=}}{:=}G_{_{=}}(\mathcal {S})$ contains an induced $P_4 = a-b-c-d$ and, therefore, $ac,ad,bd \notin E(G_{_{=}})$. Since $G_{_{=}}$, $G_{_{<}}{:=}G_{_{<}}(\mathcal {S})$ and $G_{_{>}}{:=}G_{_{>}}(\mathcal {S})$ are edge-disjoint, and $G_{_{<}}$ and $G_{_{>}}$ are cographs (cf. Lemmas 14 and 15), the cases $ac,ad,bd \in E(G_{_{<}})$ and $ac,ad,bd \in E(G_{_{>}})$ are not possible because otherwise $b-d-a-c$ is an induced $P_4$. Moreover, $ab\vert c, bc\vert a \in \mathcal {F}_T(\mathcal {G}(\mathcal {S}))$ as well as $cd\vert b,bc\vert d \in \mathcal {F}_T(\mathcal {G}(\mathcal {S}))$ and thus *T* displays neither of these two triples by Prop. 2. We consider two cases:

If $ad\in E(G_{_{<}})$ then at most one of the edges *ac* and *bd* can be contained in $G_{_{<}}$. Suppose, for contradiction, that $ac \in E(G_{_{>}})$ and $bd \in E(G_{_{<}})$. Then $ad, bd\in E(G_{_{<}})$ and $ac,bc,cd\notin E(G_{_{<}})$. Prop. 2 implies that *T* displays the informative triples $ad\vert c$ and $bd\vert c$. Hence, *T* also displays $ab\vert c$; a contradiction to $ab\vert c \in \mathcal {F}_T(\mathcal {G}(\mathcal {S}))$. By similar arguments, $ac \in E(G_{_{<}})$ and $bd \in E(G_{_{>}})$ implies that *T* displays $cd\vert b$; a contradiction to $cd\vert b \in \mathcal {F}_T(\mathcal {G}(\mathcal {S}))$. This leaves $ac,bd \in E(G_{_{>}})$ as the only possible case.

If $ad\in E(G_{_{>}})$ then at most one of the edges *ac* and *bd* can be contained in $G_{_{>}}$. Suppose, for contradiction, that $ac \in E(G_{_{>}})$ and $bd \in E(G_{_{<}})$. Then $bd\in E(G_{_{<}})$ and $ab, ad\notin E(G_{_{<}})$. Prop. 2 implies that *T* displays $bd\vert a$. Moreover, $ac,ad\in E(G_{_{>}})$ and $cd\notin E(G_{_{>}})$ imply that *T* displays $cd\vert a$. Thus, *T* displays $bc\vert a$; a contradiction. By similar arguments, $ac \in E(G_{_{<}})$ and $bd \in E(G_{_{>}})$ implies that *T* displays $bc\vert d$; a contradiction to $bc\vert d \in \mathcal {F}_T(\mathcal {G}(\mathcal {S}))$. Again, we are left with $ac,bd \in E(G_{_{<}})$ as the only possibility.

In summary, we have $ad\in E(G_{_{<}})$ and $ac,bd \in E(G_{_{>}})$ or $ad\in E(G_{_{>}})$ and $ac,bd \in E(G_{_{<}})$, and thus both $\{a,b,d\}$ and $\{a,c,d\}$ form a rainbow triangle in the edge coloring defined by $\mathcal {G}(\mathcal {S})$. $\square$

As an immediate consequence, we obtain

### Corollary 4

If the edge-coloring defined by $\mathcal {G}(\mathcal {S})$ does not contain a rainbow triangle, then $G_{_{=}}(\mathcal {S})$ is a cograph.

**Fig. 5:**
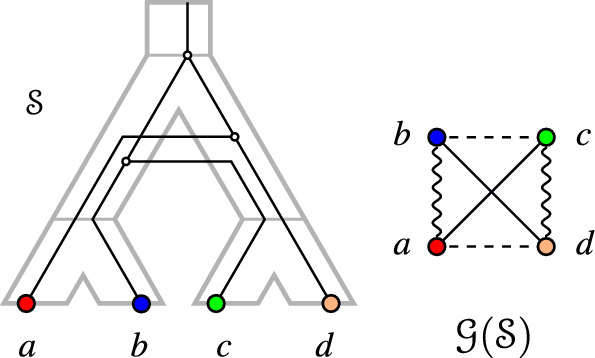
Example of a relaxed scenario $\mathcal {S}$ and corresponding graph 3-partition $\mathcal {G}(\mathcal {S})$ with $\mathcal {G}(\mathcal {S})$ containing rainbow triangles and $G_{_{=}}(\mathcal {S})$ being a cograph

The converse of Cor. 4, however, is not true in general. A counterexample is given in Fig. 5.

### Lemma 20

Let $\mathcal {S}=(T,S,\sigma ,\mu ,\tau _{T},\tau _{S})$ be a relaxed scenario. Suppose that $(G_{_{=}}(\mathcal {S}),\sigma )$ contains an induced $P_4 = a-b-c-d$ on four distinct colors $\sigma (a)=A$, $\sigma (b)=B$, $\sigma (c)=C$, and $\sigma (d)=D$. Then, exactly one of the following alternatives holds:

(i) $$ad\in E(G_{_{<}}(\mathcal {S}))$$, $$ac,bd \in E(G_{_{>}}(\mathcal {S}))$$, $$S_{\vert ABCD} = ((A,C),(B,D))$$ and $$T_{\vert abcd} = ((a,d),b,c)$$ or
(ii) $$ad\in E(G_{_{>}}(\mathcal {S}))$$, $$ac,bd \in E(G_{_{<}}(\mathcal {S}))$$, $$S_{\vert ABCD} = ((A,D),B,C)$$ and $$T_{\vert abcd} = ((a,c),(b,d))$$.

### *Proof*

Set $\mathcal {G}{:=}\mathcal {G}(\mathcal {S})$, $G_{_{<}}{:=}G_{_{<}}(\mathcal {S})$, $G_{_{=}}{:=}G_{_{=}}(\mathcal {S})$, and $G_{_{>}}{:=}G_{_{>}}(\mathcal {S})$. By Lemma 19, we have exactly one of the following two alternatives (i’) $ad\in E(G_{_{<}})$ and $ac,bd \in E(G_{_{>}})$ or (ii’) $ad\in E(G_{_{>}})$, $ac,bd \in E(G_{_{<}})$.

*Case (i’):* Since $ac,bd \in E(G_{_{>}})$ and $ab,bc,cd \notin E(G_{_{>}})$, *S* displays the informative triples $AC\vert B, BD\vert C \in \mathcal {R}_S(\mathcal {G})$ by Prop 2. Hence, $S_{\vert ABCD} = ((A,C),(B,D))$. Furthermore, by Prop. 2, *T* displays $ad\vert b, ad\vert c\in \mathcal {R}_T(\mathcal {G})$ and none of $ab\vert c, bc\vert a, bc\vert d, cd\vert b \in \mathcal {F}_T(\mathcal {G})$. If *T* displays $ac\vert b$, then this together with *T* displaying $ad\vert b$ implies that *T* also displays $cd\vert b$; a contradiction. Thus, it holds $T_{\vert abc}=(a,b,c)$. Together with the fact that *T* displays $ad\vert b$, this implies $T_{\vert abcd} = ((a,d),b,c)$. In summary, Case (i) is satisfied.

*Case (ii’):* Since $ac,bd \in E(G_{_{<}})$ and $ab,bc,cd \notin E(G_{_{>}})$, *T* displays the informative triples $ac\vert b, bd\vert c \in \mathcal {R}_T(\mathcal {G})$ by Prop. 2. Hence, $T_{\vert abcd} = ((a,c),(b,d))$. Furthermore, by Prop 2, *S* displays $AD\vert B, AD\vert C\in \mathcal {R}_S(\mathcal {G})$ and none of $AB\vert C, BC\vert A, BC\vert D, CD\vert B \in \mathcal {F}_S(\mathcal {G})$. Re-using analogous arguments as for *T* in Case (i’), we conclude that $S_{\vert ABCD} = ((A,D),B,C)$. In summary, Case (ii) is satisfied. $\square$

**Fig. 6:**
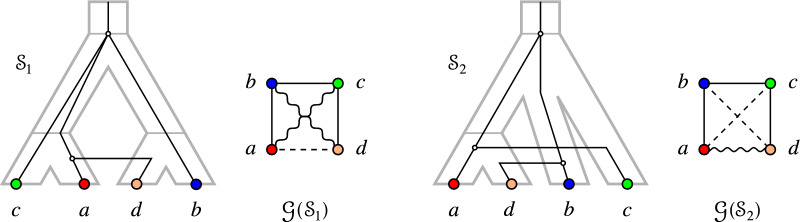
$(G_{_{=}}(\mathcal {S}_1),\sigma )=(G_{_{=}}(\mathcal {S}_2),\sigma )$ contains an induced path $P_4 = a-b-c-d$ on four colors as in Lemma 20

Cor. 1 implies that two adjacent vertices in $G_{_{=}}(\mathcal {S})$ cannot have the same color. The $2-$, $3-$ and 4-colored $P_4$s considered in Lemmas 16, 17, 18, and 20 therefore cover all possible colorings of an induced $P_4$ in $(G_{_{=}}(\mathcal {S}),\sigma )$. Moreover, in each case, the existence of a $P_4$ in $(G_{_{=}}(\mathcal {S}),\sigma )$ implies that at least one of *S* and *T* is non-binary. We summarize this discussion and Lemmas 14 and 15 in the following

### Theorem 7

Let $\mathcal {S}=(T,S,\sigma ,\mu ,\tau _{T},\tau _{S})$ be a relaxed scenario. Then $G_{_{<}}(\mathcal {S})$ and $G_{_{>}}(\mathcal {S})$ are cographs. If both *S* and *T* are binary trees, then $G_{_{=}}(\mathcal {S})$ is also a cograph.

In the case of HGT-free scenarios, the condition that *S* and *T* are binary is no longer necessary:

### Lemma 21

Let $\mathcal {S}$ be a relaxed scenario without HGT-edges. Then $G_{_{=}}(\mathcal {S})$ is a cograph.

### *Proof*

By Cor. 2, $G_{_{<}}(\mathcal {S})$ is edge-less. Therefore, $G_{_{=}}(\mathcal {S})$ is the complement of the cograph $G_{_{>}}(\mathcal {S})$ (cf. Lemma 15) and thus, by Prop. 1, also a cograph. $\square$

**Fig. 7:**
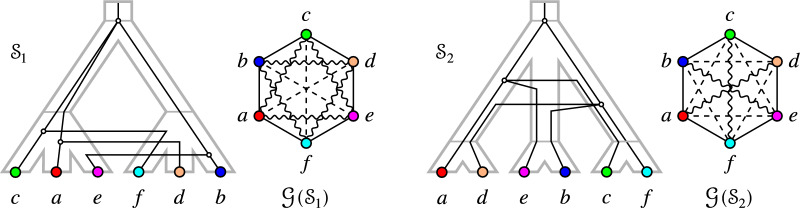
The EDT graph may contain an induced $C_6$, i.e, a cycle on six vertices. In this case, the EDT graph also contains induced $P_5$s

The similarities of $\mathcal {G}$ and edge-colored permutations graphs noted above naturally lead to the question whether $G_{_{=}}$ is a permutation graph. The example in Fig. 7 shows that this is not the case, however: The cycle on six vertices, $C_6$, is not a permutation graph [26].

### Lemma 22

If $\mathcal {S}$ is a relaxed scenario, then $G_{_{=}}(\mathcal {S})$ does not contain an induced $P_6$.

### *Proof*

Set $G_{_{<}}{:=}G_{_{<}}(\mathcal {S})$, $G_{_{=}}{:=}G_{_{=}}(\mathcal {S})$, and $G_{_{>}}{:=}G_{_{>}}(\mathcal {S})$. Suppose, for contradiction, that $G_{_{=}}$ contains an induced $P_6=a-b-c-d-e-f$ (where the colors of these six vertices are not necessarily all distinct). Since $a-b-c-d$ is an induced $P_4$ in $G_{_{=}}$ in this case, Lemma 19 implies that either (i) $ad\in E(G_{_{<}})$ and $ac,bd \in E(G_{_{>}})$ or (ii) $ad\in E(G_{_{>}})$ and $ac,bd\in E(G_{_{<}})$. Consider Case (i). Since $b-c-d-e$ is an induced $P_4$ in $G_{_{=}}$ and $bd\in E(G_{_{>}})$, Lemma 19 implies $be\in E(G_{_{<}})$ and $ce \in E(G_{_{>}})$. Repeating this argument for the induced $P_4$ $c-d-e-f$ in $G_{_{=}}$ now yields $cf\in E(G_{_{<}})$ and $df \in E(G_{_{>}})$. Consider the pair *af*. If $af\in E(G_{_{<}})$, then $G_{_{<}}$ contains the induced $P_4$ $d-a-f-c$, a contradiction to Lemma 14. Similarly, if $af\in E(G_{_{>}})$, then $G_{_{<}}$ contains the induced $P_4$ $d-f-a-c$, a contradiction to Lemma 15. Thus, only $af\in E(G_{_{=}})$ remains, which contradicts that $a-b-c-d-e-f$ is an induced $P_6$ in $G_{_{=}}$. Case (ii) is not possible for analogous reasons. Hence, $G_{_{=}}(\mathcal {S})$ cannot contain an induced $P_6$. $\square$

$P_6$-free graphs have been characterized in [27, 28]. Since any induced $P_k$ with $k\ge 6$ also contains an induced $P_6$, Lemma 22 implies that the longest possible induced path in an EDT graph has 5 vertices. Figure 7 shows that this situation can indeed be realized. In particular, the $P_5$s in these examples are part of induced cycles on six vertices. Using Lemma 19 and the arguments in the proof of Lemma 22, we can conclude that $\mathcal {G}(\mathcal {S}_1)$ and $\mathcal {G}(\mathcal {S}_2)$, as shown in Fig. 7, are the only two configurations for an induced $C_6$ that can appear in an EDT graph.

A graph is *odd-hole free* it it does not contain an induced cycle of odd length greater than three [29].

### Proposition 3

If $\mathcal {S}$ is a relaxed scenario, then $G_{_{=}}(\mathcal {S})$ does not contain an induced $C_5$ and induced cycles $C_\ell$ on $\ell \ge 7$ vertices. In particular, EDT graphs are odd-hole free.

### *Proof*

Set $G_{_{<}}{:=}G_{_{<}}(\mathcal {S})$, $G_{_{=}}{:=}G_{_{=}}(\mathcal {S})$, and $G_{_{>}}{:=}G_{_{>}}(\mathcal {S})$. Suppose, for contradiction, that $G_{_{=}}$ contains an induced $C_5$ on vertices *a*, *b*, *c*, *d*, *e* with $ab,bc,cd,de,ea\in E(G_{_{=}})$. Thus, $a-b-c-d$ is an induced $P_4$ in $G_{_{=}}$ and Lemma 19 implies that either (i) $ad\in E(G_{_{<}})$ and $ac,bd \in E(G_{_{>}})$ or (ii) $ad\in E(G_{_{>}})$ and $ac,bd\in E(G_{_{<}})$. In Case (i), we have $ad\in E(G_{_{<}})$ and $ac,bd \in E(G_{_{>}})$. Since $b-c-d-e$ is an induced $P_4$ in $G_{_{=}}$ and $bd\in E(G_{_{>}})$, Lemma 19 implies $be\in E(G_{_{<}})$ and $ce \in E(G_{_{>}})$. Repeating this argument for the induced $P_4$ $c-d-e-a$ in $G_{_{=}}$ now yields $ac\in E(G_{_{<}})$; a contradiction. Case (ii) is not possible for analogous reasons. Hence, $G_{_{=}}(\mathcal {S})$ cannot contain an induced $C_5$. Moreover, by Lemma 22, $G_{_{=}}(\mathcal {S})$ does not contain induced $P_6$s. Since every induced $C_{\ell }$ with $\ell \ge 7$ contains an induced $P_6$, such induced cycles cannot be part of an EDT graph. In particular, this implies that EDT graphs are odd-hole free. $\square$

Prop. 3 implies that not every $P_6$-free graph $(G,\sigma )$ is an EDT graph, even if we restrict ourselves to properly-colored graphs. In particular, the cycle on 5 vertices with pairwise distinct colors is a properly colored $P_6$-free graph that is not an EDT graph. Moreover, the example in Fig. 7 shows that an EDT graph may contain induced $C_6$s, i.e., they are in general not even-hole free. Moreover, EDT graphs may contain induced $C_4$s. To see this, consider the trees $T=((a_1,a_2),(b_1.b_2))$, $S=(A,B)$ and assume that $\sigma (a_i)=A$ and $\sigma (b_i)=B$, $1\le i\le 2$. Now put $\mu (\rho _T)=\rho _S$ and $\mu ({{\,\textrm{lca}\,}}_T(a_1,a_2))=\rho _SA$ and $\mu ({{\,\textrm{lca}\,}}_T(b_1,b_2))=\rho _SB$. It is now an easy exercise to verify that $a_1, b_1, a_2, b_2$ form an induced $C_4$ in $G_{_{=}}$.

A graph *G* is *perfect*, if the chromatic number of every induced subgraph equals the order of the largest clique of that subgraph [30]. A *Berge graph* is a graph that contains no odd-hole and no odd-antihole (the complement of an odd-hole) [31]. The strong perfect graph theorem [32] asserts that a graph is perfect iff it is a Berge graph.

### Proposition 4

If $\mathcal {S}$ is a relaxed scenario, then $G_{_{=}}(\mathcal {S})$ is a perfect graph.

### *Proof*

By Prop. 3, $G_{_{=}}(\mathcal {S})$ is odd-hole free. By the strong perfect graph theorem, it suffices, therefore, to show that $G_{_{=}}(\mathcal {S})$ does not contain an odd-antihole. Assume, for contradiction, that $G_{_{=}}(\mathcal {S})$ contains an odd-antihole *K*. Its complement ${\overline{K}}$ is, thus, an odd cycle that is entirely composed of edges of $G_{_{<}}(\mathcal {S})$ and $G_{_{>}}(\mathcal {S})$. Since ${\overline{K}}$ is a cycle of odd length $\ge 5$, the edges along this cycle cannot be alternatingly taken from $G_{_{<}}(\mathcal {S})$ and $G_{_{>}}(\mathcal {S})$. In other words, in $\overline{K}$ there are at least two incident edges *ab*, *bc* that are either both contained in $G_{_{<}}(\mathcal {S})$ or $G_{_{>}}(\mathcal {S})$. In addition, ${\overline{K}}$ must contain an edge *cd* and thus, $cd\notin E(G_{_{=}}(\mathcal {S}))$. This, however, implies that $G_{_{=}}(\mathcal {S})$ contains an induced $P_4$ $c-a-d-b$. By Lemma 19, $\{c, a, b\}$ should induce a rainbow triangle, which is a contradiction since *ab* and *bc* are both either in the graph $G_{_{<}}(\mathcal {S})$ or $G_{_{>}}(\mathcal {S})$. $\square$

Since perfect graphs are closed under complementation we obtain

### Corollary 5

If $\mathcal {S}$ is a relaxed scenario, then $G_{_{<}}(\mathcal {S})\cup G_{_{>}}(\mathcal {S})$ is a perfect graph.

**Fig. 8:**
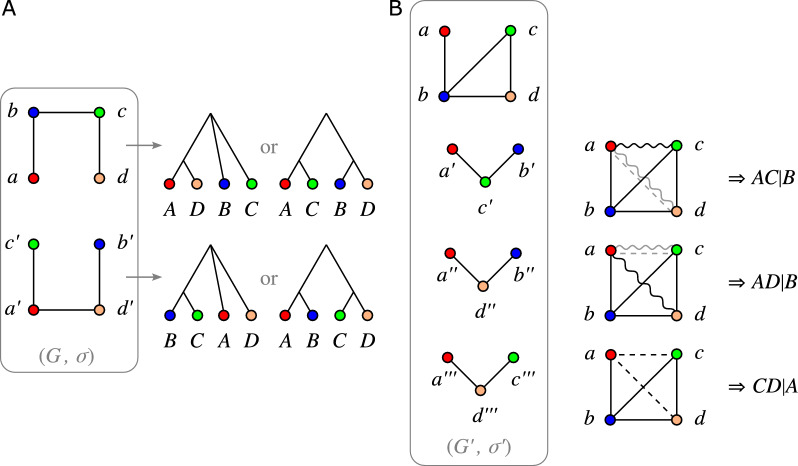
**A** A properly-colored perfect graph $(G, \sigma )$ on 8 vertices that is not an EDT graph. Next to the graph, the possible topologies of the species tree that are implied by the induced $P_4$ according to Lemma 20 are shown. **B** A properly-colored cograph $(G',\sigma ')$ that is not an EDT graph. All possible assignments for the edges *ac* and *ad* are shown on the right-hand side together with the informative triples that they imply for the species tree according to Prop. 2. The assignment of the gray edges do not affect the respective triple

The converse of Prop. 4 does not hold as shown by the examples in Fig. 8, even under the restriction to properly-colored graphs. Suppose the graph $(G,\sigma )$ in Fig. 8(A) is explained by a relaxed scenario $\mathcal {S}$. Put $A{:=}\sigma (a)=\sigma (a')$, $B{:=}\sigma (b)=\sigma (b')$, $C{:=}\sigma (c)=\sigma (c')$, and $D{:=}\sigma (d)=\sigma (d')$. By Lemma 20, the induced $P_4 = a-b-c-d$ implies that $S_{\vert ABCD} = ((A,C),(B,D))$ or $S_{\vert ABCD} = ((A,D),B,C)$, and the induced $P_4=c'-a'-d'-b'$ implies that $S_{\vert ABCD} = ((A,B),(C,D))$ or $S_{\vert ABCD} = ((B,C),A,D)$; a contradiction. Clearly, *G* contains no odd hole and no odd antihole and, thus, it is a perfect graph. Moreover, it is not sufficient to require that $(G',\sigma )$ is a properly colored cograph. To see this, suppose that the cograph $(G',\sigma ')$ in Fig. 8(B) is explained by a relaxed scenario $\mathcal {S}$. All possible assignments for the edges *ac* and *ad* are shown on the right-hand side, i.e., we have $ac\in E(G_{_{>}}(\mathcal {S}))$, $ad\in E(G_{_{>}}(\mathcal {S}))$, or $ac,ad\in E(G_{_{<}}(\mathcal {S}))$ yielding the informative triples (for the species tree *S*) $AC\vert B$, $AD\vert B$, and $CD\vert A$, respectively. However, all of these three triples are forbidden triples for *S* as a consequence of the three smaller connected components of $(G',\sigma ')$; a contradiction.
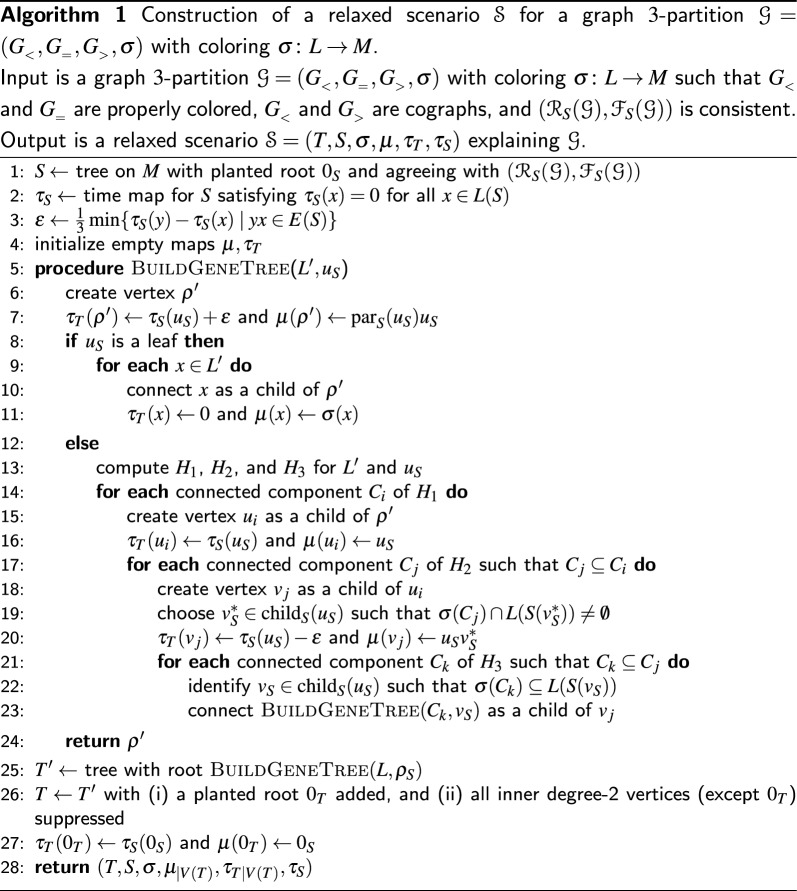

## Explanation of $\varvec{\mathcal {G}}$ by relaxed scenarios

**Fig. 9:**
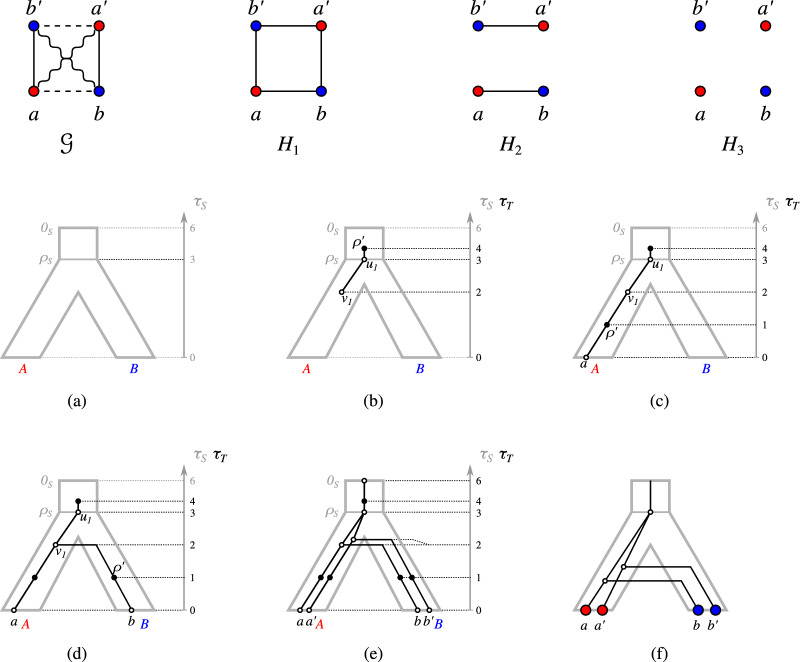
Illustration of Algorithm 1 with a valid input $\mathcal {G}= (G_{_{<}}, G_{_{=}}, G_{_{>}}, \sigma )$. We have $\sigma (a) = \sigma (a'){=:}A$ and $\sigma (b) = \sigma (b'){=:}B$. Line 1 constructs species tree *S* that agrees with $(\mathcal {R}_S(\mathcal {G}), \mathcal {F}_S(\mathcal {G}))$. Here, $\mathcal {R}_S(\mathcal {G}) = \mathcal {F}_S(\mathcal {G}) = \emptyset$ and *S* is unique. In Line 2, a time map $\tau _{S}$ for *S* such that $\tau _{S}(x)=0$ for all $x\in L(S)$ is initialized. We choose $\tau _{S}(0_S)=6$ and $\tau _{S}(\rho _S)=3$, see panel (a). Hence, $\epsilon =1$ (Line 3). Line 25 then calls BuildGeneTree$(\{a,a',b,b'\},\rho _S)$ for the first time, hence $u_S = \rho _S$. In Line 6 a vertex $\rho '$ is created. Its time map is set to $\tau _{T}(\rho ') = \tau _{S}(\rho _{S}) + \epsilon = 3+1=4$ and the reconciliation is set to $\mu (\rho ') = 0_S \rho _S$ in Line 7. Since $u_S=\rho _S$ is not a leaf, we proceed with computing $H_1$, $H_2$, and $H_3$ for $L= \{a,a',b,b'\}$ and $\rho _S$ in Line 13, illustrated in the top row. Since $H_1$ has only one connected component *C*, the for-loop in Line 14 runs only once. In Line 15, we thus create a single vertex $u_1$ as a child of $\rho '$. We then consider the two connected components $C_1$ and $C_2$ of $H_2$ as both satisfy $C_j\subseteq C$, $j\in \{1,2\}$. Here, we start with considering the component $C_1$ that is induced by the vertices *a* and *b* and create a vertex $v_1$ as a child of $u_1$ in Line 18. We choose $v^*_S = A$ in Line 19 (note that we also could have chosen $v^*_S = B$) and set $\tau _{T}(v_1) = \tau _{S}(\rho _{S}) - \epsilon = 2$ and $\mu (v_1) = \rho _S A$ in Line 20. These steps are illustrated in panel (b). Line 21 then considers the connected components $C_k$ of $H_3$ that satisfy $C_k\subseteq C_1 = \{a,b\}$; both of these connected components are the single vertex graphs induced by *a* and *b*, respectively. Starting with $C'=\{a\}$, Line 22 identifies $v_S\in {{\,\textrm{child}\,}}_S(\rho _{S})$ such that $\sigma (C')=\{A\}\subseteq L(S(v_S))$, i.e., $v_S = A$ and calls BuildGeneTree$(\{a\}, A)$; the subtree returned by this call is attached as a child of $v_1$ in Line 23. Hence, we are now back in Line 6 where $u_S = A$. In Line 6, a further (new) vertex $\rho '$ is created. Line 7 computes $\tau _{T}(\rho ') = \tau _{S}(A) + \epsilon = 0+1=1$ and $\mu (\rho ') = \rho _S A$. Now $u_S=A$ is a leaf of *S*, hence we proceed in Line 8 and connect each $x\in L' = \{a\}$ as a child of $\rho '$ in Line 10. In Line 11, we put $\tau _{T}(a) = 0$ and $\mu (a)= \sigma (a)=A$. These steps are illustrated in panel (c). Then BuildGeneTree$(\{b\}, B)$ is executed and we obtain the “partial” gene tree and reconciliation shown in panel (d). The algorithm proceeds on component $C_2$ of $H_2$, which is induced by the vertices $a'$ and $b'$ and creates a vertex $v_2$ as a child of $u_1$ in Line 18. Again, we chose $v^*_S = A$ in Line 19. By similar arguments as in the previous part, we obtain the “partial” gene tree and reconciliation shown in panel (e). The tree $T'$ returned in Line 25 is the gene tree shown in panel (e) except for the planted root $0_T$, which is added in Line 26. In addition, all resulting inner degree-2 vertices (highlighted as black circuits) are suppressed in Line 26. The resulting gene tree (without specified time map) and the resulting relaxed scenario is shown in panel (f). Note, if we choose $v^*_S = B$ in Line 19 when proceeding on the connected component $C_2$ of $H_2$ induced by $a'$ and $b'$, we would obtain the restricted scenario $\mathcal {S}_2$ as shown in Fig. 11

In [4], we derived an algorithmic approach that recognizes LDT graphs and constructs a relaxed scenario $\mathcal {S}$ for $(G_{_{<}},\sigma )$ in the positive case. Here, we adapt the algorithmic idea to the case that, instead of $(G_{_{<}},\sigma )$, the graph 3-partition $\mathcal {G}= (G_{_{<}}, G_{_{=}}, G_{_{>}}, \sigma )$ is given, see Algorithm 1, which is illustrated in Fig. 9. As we shall see, the additional information can be leveraged to separate the construction of *S* and *T* in such a way that a suitable species tree can be computed first using a well-known approach. This then considerably simplifies the construction of a corresponding gene tree *T*. More precisely, we construct the gene tree and its reconciliation with *S* in a top-down fashion via a recursive decomposition of *L* into subsets that is guided by $\mathcal {G}$ and *S*. We first introduce three auxiliary graphs that we will use for this purpose.

### Definition 8

Let $\mathcal {G}= (G_{_{<}}, G_{_{=}}, G_{_{>}}, \sigma )$ be a graph 3-partition on vertex set *L* with coloring $\sigma :L\rightarrow M$ and *S* be a tree on *M*.

For $L'\subseteq L$ and $u\in V^0(S)$ such that $\sigma (L')\subseteq L(S(u))$, we define the auxiliary graphs on $L'$:

$$\begin{aligned} H_1 {:=}\ {}&(L', E(G_{_{<}}[L']) \cup E(G_{_{=}}[L']))&\\ H_2 {:=}\ {}&(L', E(G_{_{<}}[L']) \cup \{xy \in E(G_{_{=}}[L']) \mid \sigma (x), \sigma (y)\prec _S v \text { for some } v\in {{\,\textrm{child}\,}}_S(u)\} )&\\ H_3 {:=}\ {}&(L', \{xy \mid x \text { and } y \text { are in the same connected component of } H_2 \text { and }&\\&\sigma (x), \sigma (y)\preceq _S v \text { for some } v\in {{\,\textrm{child}\,}}_S(u)\})&\end{aligned}$$

By construction, $H_2$ is a subgraph of $H_1$. In particular, therefore, every connected component of $H_2$ is entirely included in some connected component of $H_1$. In turn, one easily verifies that the connected components of $H_3$ are complete graphs. Moreover, $H_3$ contains all edges of $H_2\cap G_{_{=}}$ while there might be edges of $G_{_{<}}[L']$ that are not contained in $H_3$. This implies that every connected component of $H_3$ is entirely included in some connected component of $H_2$.

**Fig. 10:**
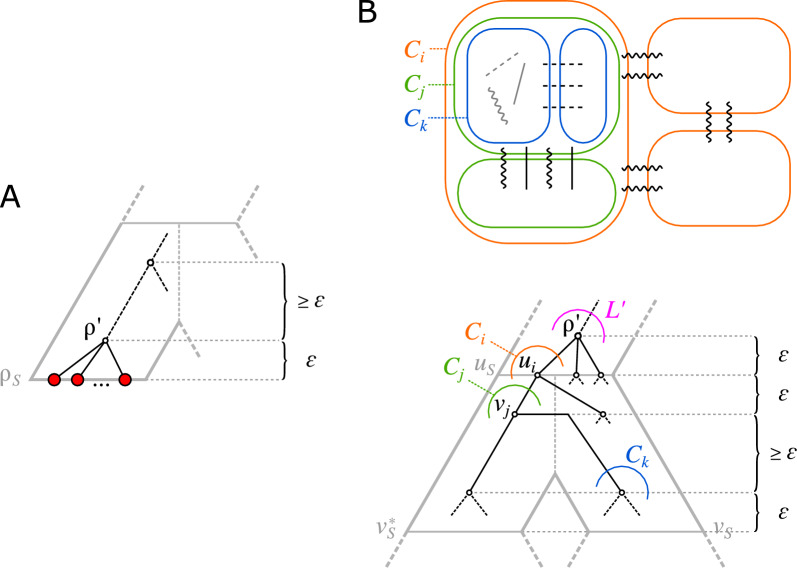
Illustration of a recursion step in BuildGeneTree (Algorithm 1). **A** The current vertex in the species tree, $u_S$, is a leaf. **B** The current vertex in the species tree, $u_S$, is an inner vertex. The connected components of $H_1$, $H_2$, and $H_3$ are represented by the orange, green, and blue boxes, respectively. For simplicity, only those connected components of $H_2$ and $H_3$ are shown that are included in $C_i$ and $C_j$, respectively. Two vertices *x* and *y* must form an edge in (a) $G_{_{>}}$ (wavy lines) if they are in distinct components of $H_1$, (b) $G_{_{>}}$ or $G_{_{=}}$ (solid straight lines) if they are in the same component of $H_1$ but distinct components of $H_2$, and (c) $G_{_{<}}$ (dashed lines) if they are in the same component of $H_2$ but distinct components of $H_3$. Below, the construction of the reconciliation map and the time map is illustrated. The half circles indicate that $L'=L(T(\rho '))$, $C_i=L(T(u_i))$, etc. if the respective vertex is not suppressed

We use the inclusion relation of the connected components to construct the local topology of *T* in a recursive manner, see Fig. 10 for an illustration of the following description. In each step, i.e., for some $L'\subseteq L$ and $u_S\in V(S)$, we first construct a “local root” $\rho '$ (cf. Algorithm 1, Line 6). If $u_S$ is a leaf of *S* (the base case of the recursion), we directly attach the elements of $L'$ as children of $\rho '$ (Lines 8–11). On the other hand, if $u_S$ is an inner vertex, we create a new child of $\rho '$ for each connected component of $H_1$ in Line 15. For a specific connected component $C_i$ of $H_1$ (corresponding to child $u_i$ of $\rho '$), we then add a new child $v_j$ of $u_i$ for each connected component $C_j$ of $H_2$ such that $C_j\subseteq C_i$ in Line 18. We proceed similarly for the connected components $C_k$ of $H_3$, which necessarily are subsets of a specific connected component $C_j$ of $H_2$. The vertex corresponding to $C_k$ is the “local root” created in a recursive call operating on $C_k$ as new subset of *L* and $v_S\in {{\,\textrm{child}\,}}_S(u_S)$ as new vertex of *S*, which is chosen such that $\sigma (C_k)\subseteq L(S(u_S))$ in Line 22. If $C_j=C_i$ or $C_k=C_j$, then the corresponding vertices $v_i$ and $v_j$, respectively, have a single child. As a consequence, the resulting tree $T'$ is in general not phylogenetic. The final gene tree *T* is then obtained by suppressing all vertices with a single child (Line 26).

By definition, two vertices *x* and *y* are in the same connected component $C_k$ of the auxiliary graph $H_3$ only if $\sigma (x)$ and $\sigma (y)$ are descendants of the same child $v_S$ of the species tree vertex $u_S$. In particular, we therefore can always find $v_S\in {{\,\textrm{child}\,}}_S(u_{S})$ such that $\sigma (C_k)\subseteq L(S(v_S))$ in Line 22 of Algorithm 1. This guarantees that all colors appearing on the vertices in $L'$ are descendants of the species tree vertex $u_S$ in each recursion step:

### Observation 2

In every recursion step of Algorithm 1, it holds $\sigma (L')\subseteq L(S(u_s))$. In particular, the auxiliary graphs $H_1$, $H_2$, and $H_3$ are always well-defined.

The recursion in Algorithm 1 can be thought of as a tree with the root being the top-level call of BuildGeneTree on *L* and $\rho _S$ and leaves being the calls in which $u_S$ is a leaf of *S*. Note that, for some recursion steps *R* on $L'$ and $u_S$, all of its “descendant recursion steps” have input $L''$ and $u'_S$ satisfying $L''\subseteq L'$ and $u'_S\prec _{S} u_S$. Therefore, and because all leaves that are descendants of $\rho '$ (created in $R'$) must have been attached in some descendant recursion step of *R*, we have $L(T'(\rho '))\subseteq L'$. In turn, all elements $x\in L'$ are either directly attached to $\rho '$ if $u_S$ is a leaf, or will eventually be passed down to a recursion step on a leaf $l\prec _{S} u_S$ because each $x\in L'$ is in some connected component $C_k$ of $H_3$, $C_k$ is entirely included in a connected component $C_j$ of $H_2$, and $C_j$ is entirely included in a connected component $C_i$ of $H_1$. In this “leaf recursion step”, *x* is therefore attached to some descendant of $\rho '$, implying $L'\subseteq L(T'(\rho '))$. Therefore, we have $L'=L(T'(\rho '))$. We can apply very similar arguments to see that $L(T'(u_i))=C_i$ and $L(T'(v_j))=C_j$ hold for each connected component $C_i$ of $H_1$ and $C_j$ of $H_2$ with corresponding vertices $u_i$ and $v_j$ created in Lines 15 and 18, respectively. Clearly, contraction of the redundant vertices to obtain the final tree *T* does not change these relationship. We summarize these considerations as follows:

### Observation 3

Let *T* be a tree returned by Algorithm 1 and $u\in V^0(T)$ be an inner vertex created in a recursion step on $L'$ and $u_S$.

1. If *u* is a vertex $\rho '$ created in Line 6, then $L(T(u))=L'$.
2. If *u* is a vertex $u_i$ created in Line 15, then $L(T(u))=C_i$ where $C_i$ is the connected component of $H_1$ corresponding to $u_i$.
3. If *u* is a vertex $v_j$ created in Line 18, then $L(T(u))=C_j$ where $C_j$ is the connected component of $H_2$ corresponding to $v_j$.

In particular, every $x\in L(T(u))$ satisfies $x\in L'$.

Algorithm 1 is a generalization of the algorithm presented in [4] for the construction of a relaxed scenario $\mathcal {S}$ for a given LDT graph $(G,\sigma )$. A key property of the algorithm is that the restriction of $\mathcal {S}$ to $S(u_S)$, i.e., the incomplete scenarios obtained for given $u_S$ satisfies the time consistency constraints (S2) and (S3). The construction of $\mathcal {S}$ in Algorithm 1 differs from the procedure described in [4] only by including in $V(T) {\setminus } (L(T) \cup \{0_T\})$ the additional vertices $u_i$ created in Line 15. These satisfy $\mu (u_i)=u_S$. In the following line, we set $\tau _{T}(u_i)\leftarrow \tau _{S}(u_S)$. Hence, constraint (S2) remains satisfied and (S3) is void because $\mu (u_i)\in V(S)$. One easily checks, furthermore, that the reconciliation map $\mu$ constructed in Algorithm 1 satisfies (S0) (Line 27) and (S1) (Line 11).

### Definition 9

$\mathcal {G}= (G_{_{<}}, G_{_{=}}, G_{_{>}}, \sigma )$ is a *valid input* for Algorithm 1 if $(G_{_{<}}, G_{_{=}}, G_{_{>}}, \sigma )$ is a 3-partition, $G_{_{<}}$ and $G_{_{=}}$ are properly colored, $G_{_{<}}$ and $G_{_{>}}$ are cographs, and $(\mathcal {R}_S(\mathcal {G}), \mathcal {F}_S(\mathcal {G}))$ is consistent.

### Lemma 23

Given a valid input $\mathcal {G}= (G_{_{<}}, G_{_{=}}, G_{_{>}}, \sigma )$ with vertex set *L*, Algorithm 1 returns a relaxed scenario $\mathcal {S}=(T,S,\sigma ,\mu ,\tau _{T},\tau _{S})$ such that $L(T)=L$.

### *Proof*

In order to keep this contribution self-contained, a detailed proof of Lemma 23, which largely parallels the material in [4], is given in Appendix . $\square$

We continue with a number of intermediate results that we will need to establish the correctness of Algorithm 1.

### Lemma 24

Let $\mathcal {G}= (G_{_{<}},G_{_{=}},G_{_{>}},\sigma )$ with vertex set *L* be a valid input for Algorithm 1. Consider a recursion step on $L'\subseteq L$ and $u_S\in V^0(S)$ of Algorithm 1. Then there are no $x,y\in L'$ in the same connected component of $H_1$ such that $xy\in E(G_{_{>}})$ and ${{\,\textrm{lca}\,}}_S(\sigma (x),\sigma (y))=u_S$.

### *Proof*

Assume for contradiction that, for some $L'$ and $u_S\in V^0(S)$ appearing in the recursion, there is a connected component $C_i$ of $H_1$ with vertices $x,y\in C_i$ and colors $X{:=}\sigma (x)$ and $Y{:=}\sigma (y)$ such that $xy\in E(G_{_{>}})$ and ${{\,\textrm{lca}\,}}_S(X,Y)=u_S$. By assumption, $u_S$ is an interior vertex and thus $X\ne Y$. Since the input $G_{_{>}}$ is a cograph, the induced subgraph $G_{_{>}}[L']$ and its complement, which by construction equals $H_1=G_{_{<}}[L'] \cup G_{_{=}}[L']$, are also cographs (cf. Prop. 1).

Consider a shortest path *P* in $H_1$ connecting *x* and *y*, which exists since $x,y\in C_i$. Since $G_{_{>}}[L']$ and $H_1$ are edge-disjoint and $xy\in E(G_{_{>}}[L'])$, *P* contains at least 3 vertices. Since $H_1$ is a cograph and thus does not contain induced $P_4$s, *P* contains at most 3 vertices. Hence, *P* is of the form $x-z-y$ and we have $xy\in E(G_{_{>}})$ and $xz, yz\notin E(G_{_{>}})$. Therefore, and since $G_{_{<}}$ and $G_{_{=}}$ are properly colored, we have $Z{:=}\sigma (z)\notin \{X,Y\}$, and thus *X*, *Y*, *Z* are pairwise distinct colors. By Prop. 2, $XY\vert Z\in \mathcal {R}_S(\mathcal {G})$. Taken together, the latter arguments and the construction of *S* in Line 1 imply that *S* displays the informative triple $XY\vert Z$. Since $x,y,z\in L'$, we have $X,Y,Z\in L(S(u_s))$ by Obs. 2. In particular, therefore, $Z\preceq _S u_S$. Thus $XY\vert Z$ implies that ${{\,\textrm{lca}\,}}_S(X,Y) \prec _S u_S$; a contradiction. $\square$

### Lemma 25

Let $\mathcal {G}= (G_{_{<}},G_{_{=}},G_{_{>}},\sigma )$ with vertex set *L* be a valid input for Algorithm 1. Consider a recursion step on $L'\subseteq L$ and $u_S\in V^0(S)$ of Algorithm 1. Then, for all $x,y \in L'$ that are contained in the same connected component of $H_2$ but in distinct connected components of $H_3$, it holds $xy\in E(G_{_{<}})$.

### *Proof*

Suppose that, for some $L'$ and $u_S\in V^0(S)$ appearing in the recursion, there is a connected component $C_j$ of $H_2$ with $x,y\in C_j$ such that *x* and *y* are in distinct connected components of $H_3$. In addition, suppose for contradiction that $xy\notin E(G_{_{<}})$. We may assume w.l.o.g. that *x* and *y* have minimal distance in $H_2$, i.e., there are no vertices $x',y'\in C_j$ such that $x'$ and $y'$ are in distinct connected components of $H_3$, $x'y'\notin E(G_{_{<}})$, and in addition the distance of $x'$ and $y'$ in $H_2$ is smaller than that of *x* and *y*. Set $X{:=}\sigma (x)$ and $Y{:=}\sigma (y)$ and let $C_x$ and $C_y$ be the connected components of $H_3$ that contain *x* and *y*, respectively. By Obs. 2, we have $\sigma (L')\subseteq L(S(u_S))$. This and the fact that *x* and *y* are in distinct connected components of $H_3$ but in the same connected component $C_j$ of $H_2$ implies that $X\preceq _S v_X$ and $Y \preceq _S v_Y$ for two distinct children $v_X,v_Y \in {{\,\textrm{child}\,}}_S(u_S)$. In particular, we have $X\ne Y$ and ${{\,\textrm{lca}\,}}_S(X,Y)=u_S$. Moreover, by construction, every connected component of $H_2$ is contained in a connected component of $H_1$ and thus, *x* and *y* are in the same connected component of $H_1$. The latter two arguments together with Lemma 24 imply $xy\notin E(G_{_{>}})$. In summary, we therefore have $xy\in E(G_{_{=}})$.

Consider a shortest path *P* connecting *x* and *y* in $H_2$, which exists since $x,y\in C_j$. By construction, $xy\in E(G_{_{=}})$ and ${{\,\textrm{lca}\,}}_S(X,Y)=u_S$ imply that $xy\notin E(H_2)$ and thus *P* contains at least 3 vertices. Let $z\in C_j\setminus \{x,y\}$ be the neighbor of *x* in *P*. We consider the two possibilities (a) $xz\in E(G_{_{=}})$ and (b) $xz\in E(G_{_{<}})$. Note that $X\ne \sigma (z) {=:}Z$ holds in both cases since $G_{_{=}}$ and $G_{_{<}}$ are properly colored.

In Case (a), we must have $Z\preceq _S v_X$ since *xz* is an edge in $H_2$. This implies that $Z\ne Y$ (and thus *X*, *Y*, *Z* are pairwise distinct) and ${{\,\textrm{lca}\,}}_S(Y,Z)=u_S$. Based on the latter arguments, *S* must display the triple $XZ\vert Y$. Suppose that $yz\notin E(G_{_{=}})$. Together with $xy,xz \in E(G_{_{=}})$, we have $XZ\vert Y\in \mathcal {F}_S$ and thus, by construction of *S* in Line 1, *S* cannot display $XZ\vert Y$; a contradiction. Hence, $yz\in E(G_{_{=}})$ must hold. Since $Z\preceq _S v_X$ and $Y\preceq _S v_Y$, we have $yz\notin E(H_3)$. Note that connected components in $H_3$ are complete graphs. Hence, $yz\notin E(H_3)$ implies that *y* and *z* are in distinct connected components of $H_3$. However, the distance of *y* and *z* in $H_2$ is strictly smaller than that of *x* and *y* (because *z* is closer to *y* than *x* in the shortest path *P*); a contradiction to our choice of *x* and *y*. In summary, Case (a) therefore cannot occur.

In Case (b) we have $xz\in E(G_{_{<}})$. If $yz\in E(G_{_{<}})$, then $Y\ne Z$ (because $G_{_{<}}$ is properly colored) and, by definition, $XY\vert Z\in \mathcal {R}_S$. By construction in Line 1, the species tree *S* displays $XY\vert Z$. Together with $X,Y,Z\in L(S(u_S))$ by Obs. 2, this contradicts that ${{\,\textrm{lca}\,}}_S(X,Y)=u_S$. Similarly, if $yz\in E(G_{_{=}})$, then *S* displays neither of the forbidden triples $XY\vert Z$ and $YZ\vert X$. Hence, *S* displays $XZ\vert Y$ or $S_{\vert XYZ}$ is the star tree on the three colors. In both cases, we have ${{\,\textrm{lca}\,}}_S(Y,Z)={{\,\textrm{lca}\,}}_S(X,Y)=u_S$. In particular, therefore *y* and *z* are in distinct connected components of $H_3$. As argued before, the distance of *y* and *z* is smaller than that of *x* and *y*. Taken together the latter arguments again contradict our choice of *x* and *y*, and thus $yz\in E(G_{_{>}})$ is left as the only remaining choice.

In summary, only case (b) $xz\in E(G_{_{<}})$ is possible, which in particular implies $yz\in E(G_{_{>}})$. Therefore, we have $yz\notin E(H_2)$ and thus the path *P* contains at least 4 vertices. Thus, consider the neighbor $w\in C_j\setminus \{x,y,z\}$ of *y* in *P* and set $W{:=}\sigma (w)$. We can apply analogous arguments for *x*, *y*, *w* as we have used for *x*, *y*, *z* to exclude the case (a’) $yw\in E(G_{_{=}})$ and, in case (b’) $yw\in E(G_{_{<}})$, we obtain $xw\in E(G_{_{>}})$ as the only possibility.

Taking the latter arguments together, it remains to consider the case $xy \in E(G_{_{=}})$, $xz, yw\in E(G_{_{<}})$, and $xw, yz\in E(G_{_{>}})$. Since $G_{_{<}}$ and $G_{_{>}}$ are cographs, we have $zw\in E(G_{_{=}})$ because otherwise $x-z-w-y$ or $x-w-z-y$ would be an induced $P_4$ in $G_{_{<}}$ and $G_{_{>}}$, respectively.

Now, *x* and *w* must be in the same connected component of $H_3$, as otherwise $xw \notin E(G_<)$ and the fact that *x* and *w* are at a shorter distance than *x* and *y* in $H_2$ would contradict our choice of *x* and *y*. Likewise, *y* and *z* are in the same connected component of $H_3$ since $yz \notin E(G_<)$ and they are closer than *x* and *y* in $H_2$. It follows that *w* and *z* are in distinct connected components of $H_3$, again yielding a contradiction since they are closer than *x* and *y* in $H_2$ and $wz \notin E(G_<)$. In summary, therefore, we have $xy\in E(G_{_{<}})$. $\square$

The following result is a consequence of Lemma 25 and will be helpful later on.

### Corollary 6

Let $\mathcal {G}= (G_{_{<}}, G_{_{=}}, G_{_{>}}, \sigma )$ with vertex set *L* be a valid input for Algorithm 1. Consider a recursion step on $L'\subseteq L$ and $u_S\in V^0(S)$ of Algorithm 1. If $xy \in E(H_1)\setminus E(H_2)$, then *x* and *y* are in distinct connected components of $H_2$.

### *Proof*

Suppose $xy \in E(H_1)\setminus E(H_2)$. By construction of the auxiliary graphs, this implies that $xy\in E(G_{_{=}})$ and there is no $v\in {{\,\textrm{child}\,}}_S(u_S)$ such that $\sigma (x),\sigma (y) \prec _{S} v$. The latter in particular yields that $xy \notin E(H_3)$. This, together with the fact that $H_3$ is a graph whose connected components are complete graphs, implies that *x* and *y* are in distinct connected components of $H_3$. We can now use Lemma 25 to conclude that *x* and *y* must also be in distinct connected components of $H_2$ as otherwise we would obtain $xy\in E(G_{_{<}})$; a contradiction. $\square$

We are now in the position to demonstrate that Algorithm 1 is correct.

### Lemma 26

Let $\mathcal {G}$ be a valid input for Algorithm 1. Then, Algorithm 1 returns a relaxed scenario $\mathcal {S}=(T,S,\sigma ,\mu ,\tau _{T},\tau _{S})$ that explains $\mathcal {G}$.

### *Proof*

Let $\mathcal {G}= (G_{_{<}}, G_{_{=}}, G_{_{>}}, \sigma )$ be a valid input with vertex set *L* for Algorithm 1. By Lemma 23, Algorithm 1 returns a relaxed scenario $\mathcal {S}=(T,S,\sigma ,\mu ,\tau _{T},\tau _{S})$ such that $L(T)=L$. We continue with showing that $\mathcal {S}$ explains $\mathcal {G}$.

Consider two distinct vertices $x,y\in L=L(T)$ and their last common ancestor ${{\,\textrm{lca}\,}}_T(x,y)$. Let $L'\subseteq L$ and $u_S\in V(S)$ be the input of the recursive call of BuildGeneTree in which ${{\,\textrm{lca}\,}}_T(x,y)$ was created. By Obs. 2 and 3, we have $\sigma (L')\subseteq L(S(u_s))$ and $x,y\in L'$, respectively, and therefore ${{\,\textrm{lca}\,}}_S(\sigma (x), \sigma (y)) \preceq _S u_S$. Moreover, time consistency yields $\tau _{S}({{\,\textrm{lca}\,}}_S(\sigma (x), \sigma (y))) \le \tau _{S}(u_S)$. The vertex ${{\,\textrm{lca}\,}}_T(x,y)$ has been created in exactly one of the following three locations in the algorithm: (a) in Line 6, (b) in Line 15, and (c) in Line 18.

In Case (a), ${{\,\textrm{lca}\,}}_T(x,y)$ equals $\rho '$ in the recursion step of interest. Suppose first that $u_S$ is a leaf of *S* and thus $\sigma (x)=\sigma (y)=u_S$. Hence, we have $xy\in E(G_{_{>}}(\mathcal {S}))$ by Cor. 1 and $xy\in E(G_{_{>}})$, since $G_{_{<}}$ and $G_{_{=}}$ are properly colored. Now suppose that $u_S$ is not a leaf. Then ${{\,\textrm{lca}\,}}_T(x,y)=\rho '$ implies that *x* and *y* lie in distinct connected components of the auxiliary graph $H_1$ and thus $xy\notin E(H_1)$. By construction of this graph, the latter yields $xy\in E(G_{_{>}})$. Moreover, we have set $\tau _{T}(\rho ')=\tau _{S}(u_S)+\epsilon > \tau _{S}(u_S)$. Together with ${{\,\textrm{lca}\,}}_T(x,y)=\rho '$, this implies $\tau _{S}({{\,\textrm{lca}\,}}_S(\sigma (x), \sigma (y))) \le \tau _{S}(u_S) < \tau _{T}({{\,\textrm{lca}\,}}_T(x,y))$ and thus $xy\in E(G_{_{>}}(\mathcal {S}))$.

In Case (b), $u_S$ is an inner vertex of *S* and ${{\,\textrm{lca}\,}}_T(x,y)$ equals $u_i$. We have set $\tau _{T}({{\,\textrm{lca}\,}}_T(x,y)) = \tau _{T}(u_i) = \tau _{S}(u_S)$. By construction, moreover, *x* and *y* must be in the same connected component $C_i$ of $H_1$ but in distinct connected components of $H_2$. Hence, we have $xy\notin E(H_2)$ which implies $xy\notin E(G_{_{<}})$ by the construction of $H_2$. Suppose first ${{\,\textrm{lca}\,}}_S(\sigma (x), \sigma (y)) = u_S$. Then $xy\in E(G_{_{=}})$ as otherwise it would hold $xy\in E(G_{_{>}})$; a contradiction to Lemma 24. Moreover, we have $\tau _{T}({{\,\textrm{lca}\,}}_T(x,y)) = \tau _{S}(u_S)=\tau _{S}({{\,\textrm{lca}\,}}_S(\sigma (x), \sigma (y)))$ and thus $xy\in E(G_{_{=}}(\mathcal {S}))$. Now suppose ${{\,\textrm{lca}\,}}_S(\sigma (x), \sigma (y)) \prec _S u_S$ and thus, by time consistency, $\tau _{S}({{\,\textrm{lca}\,}}_S(\sigma (x), \sigma (y))) < \tau _{S}(u_S) = \tau _{T}({{\,\textrm{lca}\,}}_T(x,y))$. This yields $xy\in E(G_{_{>}}(\mathcal {S}))$. Moreover, from ${{\,\textrm{lca}\,}}_S(\sigma (x), \sigma (y)) \prec _S u_S$, we conclude that $\sigma (x),\sigma (y)\preceq _S w$ for some child $w\in {{\,\textrm{child}\,}}_S(u_S)$. Therefore, we must have $xy\in E(G_{_{>}})$ since otherwise $xy\in E(G_{_{=}})$ would imply that $xy\in E(H_2)$.

In Case (c), $u_S$ is an inner vertex of *S* and ${{\,\textrm{lca}\,}}_T(x,y)$ equals $v_j$. We have set $\tau _{T}({{\,\textrm{lca}\,}}_T(x,y)) = \tau _{T}(v_j) = \tau _{S}(u_S) - \epsilon < \tau _{S}(u_S)$. By construction, moreover, *x* and *y* must be in the same connected component $C_j$ of $H_2$ (and thus also in the same connected component $C_i$ of $H_1$) but in distinct connected components of $H_3$. This immediately implies (i) that $xy \in E(G_{_{<}})$ by Lemma 25 and (ii), by construction of $H_3$, that $\sigma (x)$ and $\sigma (y)$ lie below distinct children of $u_S$. In particular, therefore, we have ${{\,\textrm{lca}\,}}_S(\sigma (x), \sigma (y)) = u_S$ and thus $\tau _{S}({{\,\textrm{lca}\,}}_S(\sigma (x), \sigma (y))) = \tau _{S}(u_S) > \tau _{T}({{\,\textrm{lca}\,}}_T(x,y))$. This implies $xy\in E(G_{_{<}}(\mathcal {S}))$.

In summary, we have shown that $xy\in E(G_{_{<}})$ iff $xy\in E(G_{_{<}}(\mathcal {S}))$, $xy\in E(G_{_{=}})$ iff $xy\in E(G_{_{=}}(\mathcal {S}))$, and $xy\in E(G_{_{>}})$ iff $xy\in E(G_{_{>}}(\mathcal {S}))$. Since $x,y\in L$ where chosen arbitrarily and $L=L(T)$, this proves that the relaxed scenario $\mathcal {S}$ returned by the algorithm indeed explains the input $\mathcal {G}$. $\square$

As outlined in the proof of Lemma 26, edges $xy\in E(G_{_{=}})$ are considered only in Case (b) and we have ${{\,\textrm{lca}\,}}_T(x,y) = u_i$ and ${{\,\textrm{lca}\,}}_S(\sigma (x), \sigma (y)) = u_S$. In this case, we put $\mu (u_i) = u_S$ in Line 16 of Algorithm 1. The reconciliation map $\mu$ therefore has the following property:

### Observation 4

Let $\mathcal {S}$ be a scenario produced by Algorithm 1 for a valid input $\mathcal {G}=(G_{_{<}}, G_{_{=}}, G_{_{>}}, \sigma )$. Then $xy\in E(G_{_{=}})$ implies $\mu ({{\,\textrm{lca}\,}}_T(x,y)) = {{\,\textrm{lca}\,}}_S(\sigma (x),\sigma (y))$.

A main result of this section is the following characterization of graph 3-partitions that derive from relaxed scenarios:

### Theorem 10

A graph 3-partition $\mathcal {G}=(G_{_{<}}, G_{_{=}}, G_{_{>}}, \sigma )$ can be explained by a relaxed scenario if and only if $G_{_{<}}$ and $G_{_{=}}$ are properly colored, $G_{_{<}}$ and $G_{_{>}}$ are cographs, and $(\mathcal {R}_S(\mathcal {G}), \mathcal {F}_S(\mathcal {G}))$ is consistent.

### *Proof*

Suppose first that $\mathcal {G}$ can be explained by a relaxed scenario. Then $G_{_{<}}$ and $G_{_{=}}$ are properly colored by Cor. 1, $G_{_{<}}$ and $G_{_{>}}$ are cographs by Lemmas 14 and 15, respectively, and $(\mathcal {R}_S(\mathcal {G}), \mathcal {F}_S(\mathcal {G}))$ is consistent by Prop. 2. Conversely, suppose $G_{_{<}}$ and $G_{_{=}}$ are properly colored, $G_{_{<}}$ and $G_{_{>}}$ are cographs, and $(\mathcal {R}_S(\mathcal {G}), \mathcal {F}_S(\mathcal {G}))$ is consistent. In this case, $\mathcal {G}= (G_{_{<}}, G_{_{=}}, G_{_{>}}, \sigma )$ is a valid input for Algorithm 1 and Lemma 26 implies that Algorithm 1 returns a relaxed scenario that explains $\mathcal {G}$. $\square$

This result implies almost immediately that the property of being explainable by a relaxed scenario is hereditary:

### Corollary 7

A graph 3-partition $\mathcal {G}=(G_{_{<}}, G_{_{=}}, G_{_{>}}, \sigma )$ with vertex set *L* can be explained by a relaxed scenario if and only if $\mathcal {G}_{\vert L'}$ can be explained by a relaxed scenario for all subsets $L'\subseteq L$.

### *Proof*

The *if*-part is clear as $\mathcal {G}=\mathcal {G}_{\vert L}$. Conversely, suppose that $\mathcal {G}=(G_{_{<}}, G_{_{=}}, G_{_{>}}, \sigma )$ is explained by a relaxed scenario $\mathcal {S}=(T,S,\sigma ,\mu ,\tau _{T},\tau _{S})$ and let $L'\subseteq L$. By Prop. 2, therefore, *S* agrees with $(\mathcal {R}_S(\mathcal {G}), \mathcal {F}_S(\mathcal {G}))$. By Thm. 10, $G_{_{<}}$ and $G_{_{=}}$ are properly colored and $G_{_{<}}$ and $G_{_{>}}$ are cographs. Now consider $\mathcal {G}_{\vert L'} = (G_{_{<}}[L'], G_{_{=}}[L'], G_{_{>}}[L'], \sigma _{\vert L'})$. Clearly, the induced subgraphs $G_{_{<}}[L']$ and $G_{_{=}}[L']$ are also properly colored. By Prop. 1, $G_{_{<}}[L']$ and $G_{_{>}}[L']$ are also cographs. By definition of the informative and forbidden triples in Def. 6 and the induced subgraph relationships, we observe furthermore that $\mathcal {R}_S(\mathcal {G}_{\vert L'}) \subseteq \mathcal {R}_S(\mathcal {G})$ and $\mathcal {F}_S(\mathcal {G}_{\vert L'}) \subseteq \mathcal {F}_S(\mathcal {G})$. Hence, *S* displays all triples in $\mathcal {R}_S(\mathcal {G}_{\vert L'})$ and none of the triples in $\mathcal {F}_S(\mathcal {G}_{\vert L'})$, which yields that $(\mathcal {R}_S(\mathcal {G}_{\vert L'}), \mathcal {F}_S(\mathcal {G}_{\vert L'}))$ is consistent. We can now again apply Thm. 10 to conclude that $\mathcal {G}_{\vert L'}$ is explainable. $\square$

Using the characterization in Thm. 10, we can decide in polynomial time whether a graph 3-partition is explainable by a relaxed scenario:

### Corollary 8

It can be decided in $O(\vert L\vert ^4 \log \vert L\vert )$ time whether a graph 3-partition $\mathcal {G}=(G_{_{<}}, G_{_{=}}, G_{_{>}}, \sigma )$ can be explained by a relaxed scenario.

### *Proof*

It can be checked in $O(\vert L\vert ^2)$ time whether $G_{_{<}}$ and $G_{_{=}}$ are properly colored. It can be decided in in $O(\vert L\vert + \vert E\vert )$ time whether a graph $G=(L,E)$ is a cograph [33]. In particular, it can also be verified in $O(\vert L\vert ^2)$ time that $G_{_{<}}$ and $G_{_{>}}$ are cographs. Extraction of $\mathcal {R}{:=}\mathcal {R}_S(\mathcal {G})$ and $\mathcal {F}{:=}\mathcal {F}_S(\mathcal {G})$ according to Def. 6 requires $O(\vert L\vert ^3)$. Let $M'\subseteq M$ be the subset of colors that appear on the leaves of the triples in $\mathcal {R}\cup \mathcal {F}$. By construction, we have $\vert M'\vert \in O(\vert L\vert )$. The algorithm MTT, which stands for *mixed triplets problem restricted to trees* and was described in [34], constructs a tree on $M'$ that agrees with $(\mathcal {R},\mathcal {F})$, if one exists, in $O(\vert \mathcal {R}\vert \cdot \vert M'\vert + \vert \mathcal {F}\vert \cdot \vert M'\vert \log \vert M'\vert + \vert M'\vert ^2 \log \vert M'\vert )$ time. This, together with $\vert \mathcal {R}\vert , \vert \mathcal {F}\vert \in O(\vert L\vert ^3)$ and $\vert M'\vert \in O(\vert L\vert )$ implies that it can be decided in $O(\vert L\vert ^4\log \vert L\vert )$ whether $(\mathcal {R},\mathcal {F})$ is consistent. $\square$

In particular, it can be decided in $O(\vert L\vert ^4 \log \vert L\vert )$ whether $\mathcal {G}=(G_{_{<}}, G_{_{=}}, G_{_{>}}, \sigma )$ can be explained by a relaxed scenario without explicit construction of such a scenario. We will show in the following that the construction of relaxed scenarios is bounded by the same complexity. For simplicity, we will explicitly require that $\sigma :L\rightarrow M$ is surjective, i.e., that $\sigma (L)=M$ holds. One easily verifies, however, that the existence of “unused colors” in *M* only increases the size of the species tree *S* (in particular, the number of leaves in *S* that are attached to $\rho _S$) but does not affect the existence of a relaxed scenario that explains $\mathcal {G}$.

### Lemma 27

Algorithm 1 can be implemented to run in $O(\vert L\vert ^4\log \vert L\vert )$ time (for valid inputs $\mathcal {G}=(G_{_{<}}, G_{_{=}}, G_{_{>}}, \sigma )$ such that $\sigma$ is surjective).

### *Proof*

Let $\mathcal {G}=(G_{_{<}}, G_{_{=}}, G_{_{>}}, \sigma )$ with vertex set *L* be a valid input and surjective coloring $\sigma :L\rightarrow M$ that is given as input for Algorithm 1. By assumption, $G_{_{<}}$ and $G_{_{=}}$ are properly colored, $G_{_{<}}$ and $G_{_{>}}$ are cographs, and $(\mathcal {R}_S(\mathcal {G}), \mathcal {F}_S(\mathcal {G}))$ is consistent. Extraction of $\mathcal {R}{:=}\mathcal {R}_S(\mathcal {G})$ and $\mathcal {F}{:=}\mathcal {F}_S(\mathcal {G})$ according to Def. 6 requires $O(\vert L\vert ^3)$ operations. As argued in the proof of Corollary 8, a tree *S* on *M* that agrees with $(\mathcal {R},\mathcal {F})$ can be constructed in $O(\vert L\vert ^4\log \vert L\vert )$ time using algorithm MTT [34].

A suitable time map $\tau _{S}$ can be constructed in $O(\vert M\vert )=O(\vert L\vert )$ time by Lemma 1.

We can employ the LCA data structure described by Bender et al. [35], which pre-processes *S* in $O(\vert M\vert )=O(\vert L\vert )$ time to allow *O*(1)-query of the last common ancestor of pairs of vertices in *S* afterwards. In addition, we want to access the vertex $w\in {{\,\textrm{child}\,}}_{S}(u)$ satisfying $v\preceq _{S} w$ for two given vertices $u,v\in V(T)$ with $v\prec _{S} u$. To achieve this, we pre-process *S* as follows: We first compute ${{\,\textrm{depth}\,}}(v)$ for each $v\in V(T)$, i.e., the number of edges on the path from the root to *v* in a top-down traversal of *S* in $O(\vert L\vert )$ time. The *Level Ancestor (LA) Problem* asks for the ancestor ${{\,\textrm{LA}\,}}(v,d)$ of a given vertex *v* that has depth *d*, and has solutions with $O(\vert L\vert )$ pre-processing and *O*(1) query time [36, 37]. Hence, we can obtain the desired vertex *w* as ${{\,\textrm{LA}\,}}(v,{{\,\textrm{depth}\,}}(u)+1)$ in constant time.

Since $\sigma (L')\subseteq L(S(u_S))$ always holds by Obs. 2, every $x\in L$ appears at most once in a loop corresponding to Line 9. Hence, the total effort of handling the cases where $u_S$ is a leaf is bounded by $O(\vert L\vert )$. Consider now one execution of BuildGeneTree (without the recursive calls) in which $u_S$ is not a leaf. Construction of the auxiliary graphs $H_1$ and $H_2$ is done in $O(\vert L'\vert ^2)$, where the condition $\sigma (x),\sigma (y) \prec _{S} v$ for some $v\in {{\,\textrm{child}\,}}_S(u_S)$ in the construction of $H_2$ is equivalent to querying the LCA data structure in *O*(1) time whether ${{\,\textrm{lca}\,}}_S(\sigma (x),\sigma (y))\ne u_S$. The connected components of $H_1$ can be obtained in $O(\vert L'\vert + \vert E(H_1)\vert )=O(\vert L'\vert ^2)$ time using breadth-first search. Since $H_2$ is a subgraph of $H_1$, we can, for each connected component $C_i$ of $H_1$, determine the connected components $C_j$ of $H_2$ with $C_j\subseteq C_i$ again using breadth-first search and only the vertices in $C_i$ as start vertices. The overall effort for this is again bounded by $O(\vert L'\vert + \vert E(H_1)\vert )=O(\vert L'\vert ^2)$. We can now, for each connected component $C_j$ of $H_2$, construct the connected components $C_k$ of $H_3$ with $C_k\subseteq C_j$ by (i) adding the edge *xy* to $H_3$ if ${{\,\textrm{lca}\,}}_S(\sigma (x),\sigma (y)) \ne u_S$ for all $x,y\in C_j$ and (ii) performing breadth-first search on $H_3$ using only the vertices in $C_j$ as start vertices. Again, the overall effort for these breadth-first searches is bounded by $O(\vert L'\vert ^2)$. The number of connected component of the three graph $H_1$, $H_2$, and $H_3$ is bounded by $O(\vert L'\vert )$. For each connected component $C_j$ of $H_2$, we have to choose $v^*_S\in {{\,\textrm{child}\,}}_S(u_{S})$ such that $\sigma (C_j)\cap L(S(v^*_S))\ne \emptyset$ in Line 19. To this end, we pick $x\in C_j$ arbitrarily and query $v^*_S={{\,\textrm{LA}\,}}(\sigma (x), {{\,\textrm{depth}\,}}(u_S)+1)$. For each connected component $C_k$ of $H_3$, we can find $v_S\in {{\,\textrm{child}\,}}_S(u_{S})$ such that $\sigma (C_k)\subseteq L(S(v_S))$ in Line 22 in the same way. In summary, for each connected component of each graph, the effort of creating a new vertex (in case of $H_1$ and $H_2$), attaching the vertex to the tree ($H_1$, $H_2$, and $H_3$), choosing $v^*_S$ in Line 19 ($H_2$), choosing $v_S$ in Line 22 ($H_3$), and assigning the values for $\tau _{T}$ and $\mu$ for the newly created vertices are all constant-time operations. The overall effort for one recursion step (excluding the recursive calls) is therefore bounded by $O(\vert L' \vert ^2)$.

To bound the total effort of BuildGeneTree, consider the recursion tree *R* of the algorithm and let *d* be its maximum depth (i.e. the maximum distance from $\rho _R$ to a leaf). Notice that when a recursion receives $u_S \in V(S)$ as input, it passes a child of $u_S$ to any recursive call that it makes. Since terminal calls occur on leaves of *S*, it follows that *d* is at most the height of *S*, which is $O(\vert V(S)\vert ) = O(\vert L\vert )$ under the assumption that $\sigma$ is surjective. For $r \in V(R)$, denote by $L'_r$ the set $L'$ received as input on the recursive call corresponding to *r*. If *r* is not a leaf of *R*, then notice that $\{L'_q: q \in {{\,\textrm{child}\,}}_R(r)\}$ is a partition of $L'_r$ (without repeated subsets), since a recursive call is made precisely for each connected component of $H_3$.

Let $\ell \in \{0,1,\ldots ,d\}$. We claim that for any two vertices $r, q \in V(R)$ at distance $\ell$ from $\rho _R$, $L'_r \cap L'_q = \emptyset$. This can be seen by induction, with $\ell = 0$ as the trivial base case. Consider $\ell > 0$. If *r* and *q* have the same parent, then $L'_r \cap L'_q = \emptyset$ follows from the observation that recursions partition their input $L'$ to their child calls. If *r* and *q* have distinct parents in *R*, we know by induction that $L'_{par_R(r)} \cap L'_{par_R(q)} = \emptyset$. Since recursions pass a subset of their input $L'$, $L'_r \cap L'_q = \emptyset$ holds as well. Thus our claim is true. Now, for a given depth $\ell \in \{0,1,\ldots ,d\}$, denote by $r_1, \ldots , r_k$ the set of vertices of *R* at distance $\ell$ from $\rho _R$. The total effort of these vertices is $O(\vert L'_{r_1}\vert ^2 + \ldots + \vert L'_{r_k}\vert ^2)$ and, since $\vert L'_{r_1}\vert + \ldots + \vert L'_{r_k}\vert \le \vert L\vert$ by our claim, the total time spent at depth $\ell$ is $O(\vert L\vert ^2)$. Because this holds for every depth from 0 to $d \in O(\vert L\vert )$, the total time spent in BuildGeneTree is $O(\vert L\vert ^3)$.

It only remains to argue on the time spent constructing the final output tree *T*. Note that in each recursion with corresponding vertex $r \in V(R)$, BuildGeneTree adds at most $2\vert L'_r\vert +1$ nodes to the constructed tree $T'$ (we always add $\rho '$ and, additionally, in non-terminal calls, we add one $u_i$ and one $v_j$ vertex for each of the $O(\vert L'_r\vert )$ connected components of $H_1$ and $H_2$, respectively, and in terminal calls we add $\vert L'_r\vert$ leaves). Since the vertices of *R* at the same depth $\ell$ receive pairwise disjoint $L'_r$ sets, it follows that a total of at most $O(\vert L\vert )$ vertices are added to *T* by the recursive calls at the same depth $\ell$. Since $d \in O(\vert L\vert )$, the resulting tree $T'$ has at most $O(\vert L\vert ^2)$ vertices. To obtain the final gene tree *T*, we can traverse $T'$ and suppress all vertices with a single child by removing the vertex and reconnecting its child to its parent in $(O(\vert V(T')\vert )=O(\vert L\vert ^2)$ total time.

Hence, the overall time complexity of Algorithm 1 is $O(\vert L\vert ^4 \log \vert L\vert )$. $\square$

## Explanation of $\varvec{\mathcal {G}}$ by restricted scenarios

Relaxed scenarios may contain combinations of HGT and deletion events that render the HGT event “unobservable” from extant data, because the gene family died out in the lineage from which that HGT originated. It is therefore of interest to consider more restrictive classes of scenarios that exclude such “unobservable” events. In this section, we show that if a relaxed scenario explains $\mathcal {G}$, then there is always some scenario without these “unobservable” events that also explains $\mathcal {G}$. To this end, we introduce the notion of a “witness”:

### Definition 11

Let $\mathcal {S}=(T,S,\sigma ,\mu ,\tau _{T},\tau _{S})$ be a relaxed scenario. We say that $x\in L(T)$ is a *witness* for $v\in V(T)$ if $x\preceq _T v$ and the path from *v* to *x* in *T* does not contain an HGT-edge. The scenario $\mathcal {S}$ is *fully witnessed* if every $v\in V(T)$ has a witness.

It is not difficult to verify that, in order for a relaxed scenario $\mathcal {S}=(T,S,\sigma ,\mu ,\tau _{T},\tau _{S})$ to be fully witnessed, it is necessary and sufficient that every vertex $v\in V^0(T)$ has a child *w* such that $\mu (w)\preceq _{S}\mu (v)$. In essence, this matches condition (2b) assumed in the work of Tofigh et al. [16] and is also a direct consequence of condition (O2) in [19, 38].

A vertex $x\in V(T)$ with $\mu (x)\in V(S)$ describes an evolutionary event that coincides with a *speciation*. This suggests to require additional constraints on $\mu$ that exclude scenarios that do not have a simple biological interpretation. In particular, it seems natural to prevent HGT-edges from emanating from such a vertex. This amounts to the assumption that speciations and HGT events are not allowed to be lumped into the same event (cf. [19]). Another interesting constraint on a speciation *u* is to require that they are witnessed by a pair of descendants *x* and *y* in two of the lineages that are separated by the speciation, i.e., such that $u={{\,\textrm{lca}\,}}_T(x,y)$ and $\mu ({{\,\textrm{lca}\,}}_T(x,y))={{\,\textrm{lca}\,}}_S(\sigma (x),\sigma (y))$. This condition is reminiscent, but weaker, than the Last Common Ancestor reconciliation [39, 40].

### Definition 12

A relaxed scenario $\mathcal {S}=(T,S,\sigma ,\mu ,\tau _{T},\tau _{S})$ is a *restricted scenario* if it satisfies the following three constraints:

(S4) $\mathcal {S}$ is fully witnessed.
(S5) If $\mu (u)\in V^0(S)$, then $\mu (v)\prec _{S} \mu (u)$ holds for all $v\in {{\,\textrm{child}\,}}_T(u)$.
(S6) If $\mu (u)\in V^0(S)$, then there exist at least two leaves $x,y \in L(T)$ such that ${{\,\textrm{lca}\,}}_T(x,y)=u$, both *x* and *y* are witnesses for *u*, and $\mu (u) = {{\,\textrm{lca}\,}}_S(\sigma (x),\sigma (y))$.

It is worth noting that conditions (S4), (S5), and (S6) are not necessarily satisfied by the most commonly studied classes of evolutionary scenarios. For example, the DTL scenarios considered in [38] do not need to satisfy (S5) if *S* or *T* is non-binary. In the remainder of this section, we show that—curiously enough—any data $\mathcal {G}=(G_{_{<}}, G_{_{=}}, G_{_{>}}, \sigma )$ that can be explained by a relaxed scenario can also be explained by a restricted scenario. We start by showing that Algorithm 1 already enforces some additional constraints.

### Lemma 28

Given a valid input $\mathcal {G}= (G_{_{<}}, G_{_{=}}, G_{_{>}}, \sigma )$, the scenario $\mathcal {S}=(T,S,\sigma ,\mu ,\tau _{T},\tau _{S})$ returned by Algorithm 1 satisfies (S4), i.e., it is fully witnessed.

### *Proof*

Consider the intermediate tree $T'$ constructed in Algorithm 1 which is not necessarily phylogenetic. By a slight abuse of notation, we will simply write $\mu$ and $\tau _{T}$ also for restrictions to subsets of *V*(*T*). We start with showing that each inner vertex $u\in V^0(T')$ has a child $v\in V(T')$ such that $\mu (v)\preceq _{S} \mu (u)$ and, thus, that *uv* is not an HGT edge. Let $L'\subseteq L$ and $u_S\in V(S)$ be the input of the recursive call of BuildGeneTree in which $u\in V^0(T')$ was created in one of Lines 6, 15, or 18.

Suppose first $u=\rho '$ was created in Line 6 and thus $\mu (u)={{\,\textrm{par}\,}}_S(u_S) u_S$. If $u_S$ is a leaf, then we attached all of the elements $x\in L'$ as children of *u* and set $\mu (x)=\sigma (x)$. Since $\sigma (L')\subseteq L(S(u_S))=\{u_S\}$ holds by Obs. 2, we have $\mu (x)=\sigma (x)=u_S$. Therefore, and since $L'$ is non-empty, *u* has a child *v* such that $\mu (v)= u_S \preceq _{S} {{\,\textrm{par}\,}}_S(u_S) u_S = \mu (u)$. If $u_S$ is not a leaf, then we have attached at least one vertex $u_i$ corresponding to a connected component $C_i$ of $H_1$ as a child of *u* in the same recursion step. In particular, we have set $\mu (u_i)=u_S$ in Line 16, and thus, $\mu (u_i)= u_S \preceq _{S} {{\,\textrm{par}\,}}_S(u_S) u_S = \mu (u)$.

Suppose $u=u_i$ was created in Line 15 and thus $\mu (u)=u_S$. In particular, $u=u_i$ corresponds to some connected component $C_i$ of $H_1$. Since $H_2\subseteq H_1$ there is at least one connected component $C_j$ of $H_2$ such that $C_j\subseteq C_i$ and thus we have attached at least one vertex $v_j$ as created in Line 18 as a child of *u* and set $\mu (v_j)= u_S v^*_S$ for some $v^*_S\in {{\,\textrm{child}\,}}_{S}(u_S)$. Hence, we have $\mu (v_j)= u_S v^*_S \preceq _{S} u_S = \mu (u)$.

Suppose, finally, that $u=v_j$ was created in Line 18. Hence, $v_j$ corresponds to some connected component $C_j$ of $H_2$ and we have set $\mu (v_j)= u_S v^*_S$ for some $v^*_S\in {{\,\textrm{child}\,}}_{S}(u_S)$ such that $\sigma (C_j) \cap L(S(v^*_S)) \ne \emptyset$. The latter implies that there is $x\in C_j$ such that $\sigma (x)\in L(S(v^*_S))$. By construction of the auxiliary graphs, there is a connected component $C_k$ such that $x\in C_k$ and $C_k\subseteq C_j$. Moreover, we have chosen $v_S\in {{\,\textrm{child}\,}}_{S}(u_S)$ in Line 22 such that $\sigma (C_k)\subseteq L(S(v_S))$. This together with $\sigma (x)\in L(S(v^*_S))$ and $\sigma (x)\in \sigma (C_k)$ implies that $v^*_S=v_S$. In particular, we have attached the vertex $\rho '$ as a child to $u=v_j$ that was created in Line 6 of the the recursion step $\texttt {BuildGeneTree}(C_k, v^*_S)$ and that satisfies $\mu (\rho ')= {{\,\textrm{par}\,}}_S(v^*_S) v^*_S = u_S v^*_S$. Hence, we have $\mu (\rho ')= u_S v^*_S \preceq _{S} u_S v^*_S = \mu (u)$.

In summary, each inner vertex $u\in V^0(T')$ has a child $v\in V(T')$ such that $\mu (v)\preceq _{S} \mu (u)$. Therefore and since $T'$ is finite, we can find a descendant leaf $x\in L(T')$ for each $u\in V^0(T')$ that can be reached from *u* by non-HGT-edges.

Now consider a vertex $v\in V^0(T) {\setminus } \{0_T\} \subseteq V^0(T')$. By the arguments above, we find a path $P'= (v{=:}v'_1 - v'_2 - \dots - v'_{k'}{:=}x)$ in $T'$ from *v* to some of its descendant leaves $x\in L(T')=L(T)$ that does not contain any HGT-edge, i.e., it holds $\mu (v'_{i+1}) \preceq \mu (v'_i)$ for all $1\le i < k'$. Therefore and since *T* is obtained from $T'$ by adding $0_T$ and suppression of all vertices with a single child, we have $x\prec _{T} v$ and, moreover, the path $P= (v{=:}v_1 - v_2 - \dots - v_k{:=}x)$ connecting *v* and *x* in *T* contains only vertices that are also contained in $P'$ in the same order. We therefore conclude that $\mu (v_{i+1}) \preceq \mu (v_i)$ holds for all $1\le i < k$, i.e., *P* does not contain any HGT-edge. Hence, there is a witness for each vertex $v\in V^0(T) {\setminus } \{0_T\}$ By definition, each leaf $x\in L(T)$ is a witness of itself. Finally, consider $0_T$ (and its unique child $\rho _T$). By construction, it holds $\mu (0_T)=0_S$. Therefore and since every element $z\in V(S)\cup E(S)$ satisfies $z\preceq _{S} 0_T$, we have that $\mu (\rho _T) \preceq _{S} \mu (0_T)$, and thus $0_T \rho _T$ is not an HGT-edge. Hence, every witness of $\rho _T$ is also a witness of $0_T$, which concludes the proof. $\square$

### Lemma 29

Given a valid input $\mathcal {G}= (G_{_{<}}, G_{_{=}}, G_{_{>}}, \sigma )$, the scenario $\mathcal {S}=(T,S,\sigma ,\mu ,\tau _{T},\tau _{S})$ returned by Algorithm 1 satisfies (S5), i.e., $\mu (u)\in V^0(S)$ implies that $\mu (v)\prec _{S} \mu (u)$ for all $v\in {{\,\textrm{child}\,}}_T(u)$.

### *Proof*

Suppose that $\mu (u) \in V^0(S) = V(S) {\setminus } (L(S) \cup \{0_S\})$ and let $v\in {{\,\textrm{child}\,}}_T(u)$ be an arbitrary child of *u*. Inspection of Algorithm 1 shows that *u* must have been created in Line 15 in some recursion step on $L'\subseteq L$ and $u_S\in V^0(S)$ and thus $\mu (u)=u_S$. Consider the intermediate tree $T'$ constructed in the algorithm from which *T* is obtained by adding the planted root $0_T$ and suppression of all inner vertices with a single child. In particular, the path connecting *u* and *v* in $T'$ passes through some child $v'$ of *u* in $T'$ (where $v=v'$ is possible). By construction, we have set $\mu (v')=u_S v^*_S$ for some $v^*_S\in {{\,\textrm{child}\,}}_{S} (u_S)$ in Line 20. Re-using the arguments in the proof of Lemma 28, we find a path $P= (v' {=:}v_1 - \dots - v_{k}{:=}x)$ in $T'$ from $v'$ to some of its descendant leaves $x\in L(T')=L(T)$ that satisfies $\mu (v_{i+1}) \preceq _S \mu (v_i)$ for all $1\le i < k$. If *v* lies on the path *P*, then the latter and transitivity of $\preceq _S$ immediately implies $\mu (v)\preceq _{S} \mu (v') = u_S v^*_S \prec _{S} u_S = \mu (u)$. Suppose for contradiction that *v* is not a vertex in *P*. Then there must be some vertex $v_i(\ne v)$ with $1 \le i < k$ that is the last common ancestor of *v* and *x* in $T'$. In this case, $v_i$ must have at least two children in $T'$ and thus it was not suppressed. Since $v_i$ furthermore lies on the path connecting *u* and *v*, this contradicts that $v\in {{\,\textrm{child}\,}}_T(u)$. Hence, the case that *v* is not a vertex in *P* does not occur. Therefore, we have $\mu (v) \prec _{S} \mu (u)$, which together with the fact that $v\in {{\,\textrm{child}\,}}_T(u)$ was chosen arbitrarily, implies that $\mathcal {S}$ satisfies (S5). $\square$

**Fig. 11:**
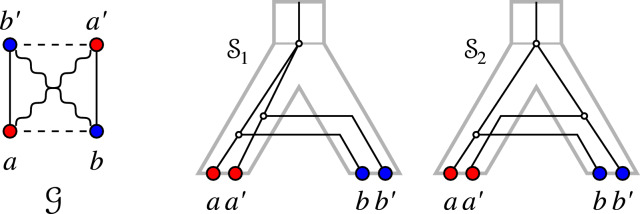
The graph 3-partition $\mathcal {G}= (G_{_{<}}, G_{_{=}}, G_{_{>}}, \sigma )$ used in Fig. 9 as illustration of Algorithm 1 is explained by different scenarios: Depending on the choice in Line 19, Algorithm 1 can return $\mathcal {S}_1$ as well as the restricted scenario $\mathcal {S}_2$. To ensure that *always* a restricted scenario is returned we provide an alternative subroutine (summarized in Algorithm 2 below) that can be used in Algorithm 1

The example in Fig. 11 shows that Algorithm 1 is in general not guaranteed to return a restricted scenario since it may violate (S6).

As we shall see in the following, however, we can construct such a scenario for any valid input $\mathcal {G}= (G_{_{<}}, G_{_{=}}, G_{_{>}}, \sigma )$ by choosing the vertex $v^*_S\in {{\,\textrm{child}\,}}_{S}(u_S)$ in Line 19 in a more sophisticated manner. More precisely, consider a connected component $C_i$ of $H_1$, for which we have created a corresponding vertex $u_i$ in Line 15). If there is only one connected component $C_j$ of $H_2$ such that $C_j\subseteq C_i$ (thus implying $C_j= C_i$), then we proceed as in the original algorithm. Otherwise, $C_i$ includes at least two connected components of $H_2$. In this case, there exists an edge $xy\in E(H_1) {\setminus } E(H_2)$ with $x,y\in C_i$. From Cor. 6 and $H_2\subseteq H_1$ we obtain $x\in C_x \subseteq C_i$ and $y\in C_y\subseteq C_i$ for two distinct connected components $C_x$ and $C_y$ of $H_2$. From the construction of the auxiliary graphs $H_1$ and $H_2$ and $\sigma (L')\subseteq L(S(u_S))$, we know that $xy \in E(G_{_{=}})$. Moreover, we have $\sigma (x)\preceq _S v_{\sigma (x)}$ and $\sigma (y)\preceq _S v_{\sigma (y)}$ for distinct vertices $v_{\sigma (x)},v_{\sigma (y)} \in {{\,\textrm{child}\,}}_S(u_S)$ because otherwise *xy* would be an edge in $H_2$. Upon encountering $C_x$ and $C_y$ during the iteration over connected components in Line 17, we simply choose $v_{\sigma (x)}$ and $v_{\sigma (y)}$ in Line 19, respectively. Notice that this is in line with the condition in Line 19 because $\sigma (x)\in \sigma (C_x)\cap L(S(v_{\sigma (x)}))$ and $\sigma (y)\in \sigma (C_y)\cap L(S(v_{\sigma (y)}))$. For all other connected components, we simply choose $v^*_S$ as in the original algorithm. These modifications of Algorithm 1 (which are restricted to the **else**-block starting in Line 12) are summarized in Algorithm 2.
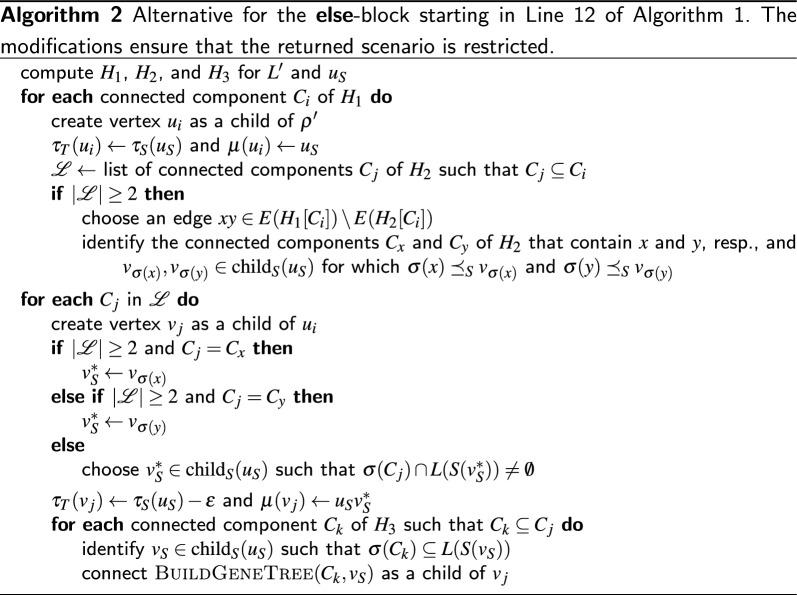


By the latter arguments we have only constrained choices that were arbitrary in the original algorithm. All results for Algorithm 1 (with exception of the complexity results) therefore remain valid for the modified version. As an immediate consequence of Lemmas 26, 28, and 29, we therefore obtain:

### Observation 5

The modifications of Algorithm 1 summarized in Algorithm 2 ensure that it returns a scenario that explains the valid input $\mathcal {G}$ and satisfies (S4) and (S5).

For completeness we show that the modifications do not increase the time complexity.

### Lemma 30

Algorithm 1 with the modifications as summarized in Algorithm 2 can be implemented to run in $O(\vert L\vert ^4 \log \vert L\vert )$ time (for valid inputs $\mathcal {G}=(G_{_{<}}, G_{_{=}}, G_{_{>}}, \sigma )$ such that $\sigma$ is surjective).

### *Proof*

Re-using the arguments in the proof of Lemma 27, it suffices to show that, in the modified algorithm, the effort of the additional steps in one recursion step on $L'\subseteq L$ and some inner vertex $u_S\in V^0(S)$ (excluding the recursive calls) is bounded by $O(\vert L'\vert ^2)$.

We have already shown in the proof of Lemma 27 how the lists $\mathscr {L}$ of connected components $C_j$ of $H_2$ such that $C_j\subseteq C_i$ are obtained using breadth-first search with a total effort of $O(\vert L'\vert ^2)$ time. We can store, for each vertex $x\in L'$, a pointer to the connected component of $H_2$ in a hash table in $O(\vert L'\vert )$ time. For a given connected component $C_i$ of $H_1$, choosing an edge $xy\in E(H_1[C_i]) \setminus E(H_2[C_i])$ is easily done by iterating over all pairs of vertices in $C_i$. Since distinct connected components of $H_1$ are vertex-disjoint, the overall effort for this is again bounded by $O(\vert L'\vert ^2)$. For a given connected component $C_i$ of $H_1$, identifying the respective connected components $C_x$ and $C_y$ and vertices $v_{\sigma (x)},v_{\sigma (y)} \in {{\,\textrm{child}\,}}_S(u_S)$ can be done in constant time by querying the above-mentioned hash table and the LA data structure, respectively. Since $H_1$ has at most $O(\vert L'\vert )$ connected components, the total effort for the latter look-ups is bounded by $O(\vert L'\vert )$. Finally, checking whether $C_j=C_x$ and $C_j=C_y$ can clearly be done in constant time if we compare only pointers to the connected components. The total time complexity of the second **for**-loop in Algorithm 2 is therefore the same as in the original algorithm.

In summary, the total effort of one recursion step (excluding the recursive calls) is still bounded by $O(\vert L'\vert ^2)$, which completes the proof. $\square$

We note that scenario $\mathcal {S}_2$ in Fig. 11 may be obtained from Algorithm 1 using the subroutine in Algorithm 2 if the edge $ab'\in E(H_1[C_i]) \setminus E(H_2[C_i])$ is chosen (over the alternative choice $a'b$) in the **“if** $\vert \mathscr {L}\vert \ge 2$ **then”** block.

### Lemma 31

Given a valid input $\mathcal {G}= (G_{_{<}}, G_{_{=}}, G_{_{>}}, \sigma )$, the scenario $\mathcal {S}=(T,S,\sigma ,\mu ,\tau _{T},\tau _{S})$ returned by Algorithm 1 with the modifications as summarized in Algorithm 2 satisfies (S6).

### *Proof*

Suppose that $\mu (u) \in V^0(S) = V(S) {\setminus } (L(S) \cup \{0_S\})$. Inspection of Algorithm 1 shows that *u* can only have been created in Line 15 in some recursion step on $L'\subseteq L$ and $u_S\in V^0(S)$. In particular, we have $\mu (u)=u_S$ and *u* corresponds to some connected component $C_i$ of $H_1$. Consider the intermediate tree $T'$ constructed in the algorithm from which *T* is obtained by adding the planted root $0_T$ and suppression of all inner vertices with a single child. Since *u* was not suppressed, we must have added at least to distinct vertices as children of *u* in the same recursion step. In particular, the output of the modified algorithm satisfies $\mu (v_j)=u_S v_S$ and $\mu (v_{j'})=u_S v'_S$ for two distinct children $v_j, v_{j'}$ of *u* and two distinct vertices $v_S,v'_S \in {{\,\textrm{child}\,}}_S(u_S)$. Re-using the arguments in the proof of Lemma 28 and the fact that $\mu (v_j)=u_S v_S \prec _S u_S = \mu (u)$, we find a path $P'= (u{=:}v'_1 - v_j {=:}v'_2 - \dots - v'_{k'}{:=}x)$ in $T'$ from *u* to some of its descendant leaves $x\in L(T')=L(T)$ that passes through $v_j$ and does not contain any HGT-edge, i.e., it holds $\mu (v'_{i+1}) \preceq \mu (v'_i)$ for all $1\le i < k'$. In particular $\sigma (x)=\mu (x)\prec _{S} \mu (v_j) = u_S v_S$. Therefore, and because *T* is obtained from $T'$ by adding $0_T$ and suppression of all vertices with a single child, we have $x\prec _{T} u$ and, moreover, the path $P= (u{=:}v_1 - v_2 - \dots - v_k{:=}x)$ connecting *u* and *x* in *T* contains only vertices that are also contained in $P'$ in the same order. We therefore conclude that $\mu (v_{i+1}) \preceq \mu (v_i)$ holds for all $1\le i < k$, i.e., *P* does not contain any HGT-edge. Analogously, we find a descendant leaf $y\prec _{S} u$ such that the path from *u* to *y* in $T'$ passes through $v_{j'}$, the path from *u* to *y* in *T* does not contain HGT-edges, and furthermore $\sigma (y)\prec _{S} u_S v'_S$.

By construction, we have ${{\,\textrm{lca}\,}}_{T'}(x,y)=u$, which implies ${{\,\textrm{lca}\,}}_{T}(x,y)=u$ since we only added $0_T$ and suppressed the vertices with a single child to obtain *T* from $T'$. The paths from *u* to *x* and to *y* in *T* do not contain HGT-edges. Thus the path from *x* to *y* in *T* does not contain HGT-edges. Finally $\sigma (x)\prec _{S} u_S v_S$ and $\sigma (y)\prec _{S} u_S v'_S$ with $v_S$ and $v'_S$ being distinct children of $u_S$ implies ${{\,\textrm{lca}\,}}_S(\sigma (x), \sigma (y)) = u_S = \mu (u)$. Taken together, the latter arguments imply that $\mathcal {S}$ satisfies (S6). $\square$

### Theorem 13

A graph 3-partition $\mathcal {G}=(G_{_{<}}, G_{_{=}}, G_{_{>}}, \sigma )$ can be explained by a relaxed scenario if and only if it can be explained by a restricted scenario. In particular, Algorithm 1 with the modifications summarized in Algorithm 2 constructs a restricted scenario in this case.

### *Proof*

The *if*-direction trivially holds since every restricted scenario is also a relaxed scenario. Conversely, suppose $\mathcal {G}$ is explained by a relaxed scenario. Then Algorithm 1 with the modifications as summarized in Algorithm 2 returns a scenario $\mathcal {S}$ that explains $\mathcal {G}$ by Lemma 26. By Lemmas 28, 29, and 31, respectively, $\mathcal {S}$ satisfies (S4), (S5), and (S6), and thus, it is a restricted scenario. $\square$

### Corollary 9

Let $\mathcal {G}=(G_{_{<}}, G_{_{=}}, G_{_{>}}, \sigma )$ be graph 3-partition with vertex coloring $\sigma :L\rightarrow M$. If $\mathcal {G}=(G_{_{<}}, G_{_{=}}, G_{_{>}}, \sigma )$ can be explained by a relaxed scenario, then, for every species tree $S^*$ on *M* that agrees with $(\mathcal {R}_S(\mathcal {G}), \mathcal {F}_S(\mathcal {G}))$, there is a relaxed scenario $\mathcal {S}=(T,S^*,\sigma ,\mu ,\tau _{T},\tau _{S})$ that explains $\mathcal {G}$. Moreover, $\mathcal {S}$ can be chosen to be a restricted scenario.

### *Proof*

Suppose $\mathcal {G}=(G_{_{<}}, G_{_{=}}, G_{_{>}}, \sigma )$ can be explained by a relaxed scenario. By Thm. 10, therefore, $\mathcal {G}$ is a valid input for Algorithm 1 with the modifications in summarized in Algorithm 2. Since the species tree *S* constructed in Line 1 of Algorithm 1 is an arbitrary tree $S^*$ on *M* that agrees with $(\mathcal {R}_S(\mathcal {G}), \mathcal {F}_S(\mathcal {G}))$, i.e., not necessarily the tree constructed by MTT [34], Obs. 5 immediately implies that there is a relaxed scenario $\mathcal {S}=(T,S^*,\sigma ,\mu ,\tau _{T},\tau _{S})$ that explains $\mathcal {G}$. Moreover, if $\mathcal {S}$ is constructed using the modified algorithm, then it is a restricted scenario by Thm. 13. $\square$

## Explanation of EDT graphs by relaxed scenarios

In the two preceding sections, we have seen that it can be decided efficiently whether a given vertex-colored graph $(G,\sigma )$ is an EDT graph provided we also know how the complement $(\overline{G},\sigma )$ is partitioned into a putative LDT graph $(G_{_{>}},\sigma )$ and putative PDT graph $(G_{_{<}},\sigma )$. It is of immediate interest to understand whether the information on $(G_{_{>}},\sigma )$ and $(G_{_{<}},\sigma )$ is necessary, or whether EDT graphs can also be recognized efficiently in isolation. We consider the following decision problem:

### Problem 1

(EDT-Recognition)

**Table:** 

*Input:*	A colored graph $(G, \sigma )$.
*Question:*	Is $(G,\sigma )$ an EDT graph?

As we shall see, EDT-Recognition can be answered in polynomial-time, if we suppose that the scenario explaining $(G, \sigma )$ is HGT-free while, for the general case, EDT-Recognition is NP-complete. We start with a characterization of the EDT graphs that can be explained by HGT-free relaxed scenarios. For this purpose, it will be useful to note that edge-less LDT graphs rule out the existence of HGT-edges in fully witnessed scenarios:

### Lemma 32

If a relaxed scenario $\mathcal {S}$ is fully witnessed and $E(G_{_{<}}(\mathcal {S}))=\emptyset$, then $\mathcal {S}$ is HGT-free.

### *Proof*

Suppose for contradiction that $\mathcal {S}=(T,S,\sigma ,\mu ,\tau _{T},\tau _{S})$ contains an HGT-edge $uv\in E(T)$ (where $v\prec _{T} u$), i.e., $\mu (u)$ and $\mu (v)$ are incomparable in *S*. By assumption, *u* has a witness $x\in L(T)$, and *v* has a witness $y\in L(T)$. In particular, it holds $\sigma (x)=\mu (x)\preceq _{S} \mu (u)$ and $\sigma (y)=\mu (y)\preceq _{S} \mu (v)$ which, together with $\mu (u)$ and $\mu (v)$ being incomparable, implies that $\mu (u)\prec _{S} {{\,\textrm{lca}\,}}_S(\sigma (x),\sigma (y))$. Moreover, since *uv* is an HGT-edge and the path from *u* to *x* does not contain an HGT-edge, *x* cannot be a descendant of *v*. Hence, ${{\,\textrm{lca}\,}}_{T}(x,y)=u$. We now distinguish cases (a) $\mu (u)\in V(S)$ and (b) $\mu (u)\in E(S)$. In Case (a), we have $\tau _{T}(u)=\tau _{S}(\mu (u))$ by Condition (S2) and $\tau _{S}(\mu (u)) < \tau _{S}({{\,\textrm{lca}\,}}_S(\sigma (x),\sigma (y)))$ as a consequence of $\mu (u)\prec _{S} {{\,\textrm{lca}\,}}_S(\sigma (x),\sigma (y))$. In Case (b), we have $\mu (u)=ab\in E(S)$ and, by Condition (S3), $\tau _{T}(u) < \tau _{S}(a)$. Moreover, $\mu (u)\prec _{S} {{\,\textrm{lca}\,}}_S(\sigma (x),\sigma (y))$ implies $a\preceq _{S} {{\,\textrm{lca}\,}}_S(\sigma (x),\sigma (y))$ by the definition of $\preceq _{S}$. Hence, we have $\tau _{T}(u) < \tau _{S}(a) \le \tau _{S}({{\,\textrm{lca}\,}}_S(\sigma (x),\sigma (y)))$. In summary, it holds $\tau _{T}({{\,\textrm{lca}\,}}_{T}(x,y))=\tau _{T}(u)<\tau _{S}({{\,\textrm{lca}\,}}_S(\sigma (x),\sigma (y)))$ and thus $xy\in E(G_{_{<}}(\mathcal {S}))$ in both cases; a contradiction to $E(G_{_{<}}(\mathcal {S}))=\emptyset$. Therefore, $\mathcal {S}$ must be HGT-free. $\square$

The recognition of EDT graphs can be achieved in polynomial-time in the HGT-free case.

### Theorem 14

Let $(G_{_{=}}=(L,E), \sigma )$ be a vertex-colored graph, and let $\mathcal {R}$ be the set of triples such that $\sigma (x)\sigma (y)\vert \sigma (z)\in \mathcal {R}$ iff $xz,yz\in E$ and $xy\notin E$ for some $x,y,z\in L$ of pairwise distinct colors. Then $(G_{_{=}},\sigma )$ is an EDT graph that can be explained by an HGT-free relaxed scenario if and only if it is a properly colored cograph and $\mathcal {R}$ is consistent. In particular, EDT graphs explained by HGT-free relaxed scenario can be recognized in $O(\vert L \vert ^3 + \vert L \vert \vert \mathcal {R}\vert )$ time.

### *Proof*

Suppose $(G_{_{=}},\sigma )$ is an EDT graph that is explained by the HGT-free relaxed scenario $\mathcal {S}$. By Cor. 1 and Lemmas 21, $(G_{_{=}},\sigma )$ is a properly colored cograph. Suppose $xz,yz\in E$ and $xy\notin E$. Since in addition $G_{_{<}}(\mathcal {S})$ is edge-less by Cor. 2, we have $xz,yz\notin E(G_{_{>}}(\mathcal {S}))$ and $xy\in E(G_{_{>}}(\mathcal {S}))$. Hence, we obtain $\mathcal {R}\subseteq \mathcal {R}_S(\mathcal {G}(\mathcal {S}))$. By Thm. 10, $\mathcal {R}_S(\mathcal {G}(\mathcal {S}))$ and thus also its subset $\mathcal {R}$ are consistent.

Now suppose $(G_{_{=}},\sigma )$ is a properly colored cograph and $\mathcal {R}$ is consistent. Consider $\mathcal {G}=(G_{_{<}}{:=}(L,\emptyset ), G_{_{=}}, G_{_{>}}{:=}\overline{G_{_{=}}})$. Since $(G_{_{<}},\sigma )$ is edge-less, it is a properly-colored cograph. Since $G_{_{>}}$ is the complement of the cograph $G_{_{=}}$, it is also a cograph. One easily verifies that $\mathcal {R}=\mathcal {R}_S(\mathcal {G})$ and thus there is a tree *S* that displays all triples in $\mathcal {R}_S(\mathcal {G})$. Now consider a triple $XZ\vert Y \in \mathcal {F}_S(\mathcal {G})$. By construction, this implies that there are $x,y,z\in L$ with pairwise distinct colors $X=\sigma (x)$, $Y=\sigma (y)$, and $Z=\sigma (z)$ such (a) $xz,yz \in E(G_{_{=}})$ and $xy\notin E(G_{_{=}})$ or (b) $xz,xy \in E(G_{_{=}})$ and $yz\notin E(G_{_{=}})$. In Case (a), we have $xz,yz \notin E(G_{_{>}})$ and $xy\in E(G_{_{>}})$ and thus *S* displays the informative triple $XY\vert Z\in \mathcal {R}_S(\mathcal {G})$. In Case (a), we have $xz,xy \notin E(G_{_{>}})$ and $yz\in E(G_{_{>}})$ and thus *S* displays the informative triple $YZ\vert X\in \mathcal {R}_S(\mathcal {G})$. Therefore, the tree *S* does not display the forbidden triple $XZ\vert Y$. Since $XZ\vert Y \in \mathcal {F}_S(\mathcal {G})$ was chosen arbitrarily, we can conclude that *S* agrees with $(\mathcal {R}_S(\mathcal {G}), \mathcal {F}_S(\mathcal {G}))$. In summary, therefore, we can apply Theorem 10 to conclude that $\mathcal {G}$ is explained by a relaxed scenario $\mathcal {S}$. By Theorem 13, $\mathcal {S}$ can be chosen to be fully witnessed. This together with the fact that $G_{_{<}}(\mathcal {S})=G_{_{<}}$ is edge-less and Lemma 32 yields that $\mathcal {S}$ is HGT-free. In summary, $(G_{_{=}},\sigma )$ is an EDT graph that can be explained by a relaxed HGT-free scenario.

Checking whether $(G=(L,E),\sigma )$ is properly colored can be done in $O(\vert E\vert )$ time, cographs can be recognized in $O(\vert L\vert +\vert E\vert )$ time [33], extraction of $\mathcal {R}$ requires $O(\vert L\vert ^3)$ time and testing whether $\mathcal {R}$ is consistent can be achieved in $O(\vert L\vert \vert \mathcal {R}\vert )$ time [41]. Thus, EDT graphs can be recognized in time $O(\vert L \vert ^3 + \vert L \vert \, \vert \mathcal {R}\vert )$ in the HGT-free case. $\square$

The examples in Fig. 8 have shown that the connected components of a given vertex-colored graph $(G,\sigma )$ are not “independent” in the sense that $(G,\sigma )$ is an EDT graph if and only if all of its connected components are EDT graphs, since the components may impose contradictory constraints on the species tree. However, we will show next that we can assume w.l.o.g. that, if a relaxed scenario $\mathcal {S}$ explaining $(G_{_{=}},\sigma )$ exists, all pairs $x,y\in L$ that are in distinct connected components of $G_{_{=}}$ form an edge in $G_{_{>}}(\mathcal {S})$. More precisely, we have

### Lemma 33

Suppose $\mathcal {G}= (G_{_{<}}, G_{_{=}}, G_{_{>}}, \sigma )$ is explained by $\mathcal {S}$ and consider the edge set $F{:=}\{xy \mid x,y\in L \text { are in distinct connected components of } G_{_{=}}\}$. Then $\mathcal {G}'=(G_{_{<}}', G_{_{=}}, G_{_{>}}', \sigma )$ where $G_{_{<}}'{:=}(L, E(G_{_{<}}) {\setminus } F)$ and $G_{_{>}}'{:=}(L, E(G_{_{>}}) \cup F)$ is explained by a relaxed scenario $\mathcal {S}'$.

### *Proof*

Observe first that all pairs $x,y\in L$ that are in distinct connected components of $G_{_{=}}$ satisfy $xy\in E(G_{_{>}}')$. By Theorem 10, $G_{_{<}}$ and $G_{_{=}}$ are properly colored, $G_{_{<}}$ and $G_{_{>}}$ are cographs, and $(\mathcal {R}_S(\mathcal {G}), \mathcal {F}_S(\mathcal {G}))$ is consistent. Since $G_{_{<}}'$ is a subgraph of $G_{_{<}}$, it is still properly colored.

Suppose for contradiction that $G_{_{<}}'$ is not a cograph, i.e., it contains an induced $P_4=a-b-c-d$. In this case, $ab,bc,cd \in E(G_{_{<}}')$ implies that $ab,bc,cd \notin F$ and thus, that *a* and *b*, *b* and *c* as well as *c* and *d* are contained in the same connected component of $G_{_{=}}$. Consequently, *a*, *b*, *c*, and *d* are contained in a single connected component of $G_{_{=}}$, which implies that $ac,bd,ad\notin F$. Therefore, $a-b-c-d$ is also an induced $P_4$ in $G_{_{<}}$; a contradiction. Now suppose for contradiction that $G_{_{>}}'$ contains an induced $P_4=a-b-c-d$. In this case, $ac,bd,ad \notin E(G_{_{>}}')$ implies $ac,bd,ad \notin G_{_{>}}$ and $ac,bd,ad \notin F$. The latter in particular implies that *a*, *b*, *c*, and *d* are contained in a single connected component of $G_{_{=}}$ and thus $ab,bc,cd \notin F$. It follows that *ab*, *bc*, and *cd* must also be edges in $G_{_{>}}$ and, thus, $a-b-c-d$ is an induced $P_4$ in $G_{_{>}}$; a contradiction. In summary, $G_{_{<}}'$ and $G_{_{>}}'$ are cographs.

We continue with showing that $(\mathcal {R}_S(\mathcal {G}'), \mathcal {F}_S(\mathcal {G}'))$ remains consistent. Suppose $XY\vert Z\in \mathcal {R}_S(\mathcal {G}')$, i.e., there are $x,y,z\in L$ with pairwise distinct colors $X=\sigma (x)$, $Y=\sigma (y)$, and $Z=\sigma (z)$ such that (a’) $xz, yz \in E(G_{_{<}}')$ and $xy \notin E(G_{_{<}}')$, or (b’) $xy \in E(G_{_{>}}')$ and $xz, yz \notin E(G_{_{>}}')$. In both cases, we can apply similar arguments as before to conclude that $xy,xz,yz\notin F$. Thus, $xz, yz \in E(G_{_{<}})$ and $xy \notin E(G_{_{<}})$, and $xy \in E(G_{_{>}})$ and $xz, yz \notin E(G_{_{>}})$, respectively. This in turn implies $XY\vert Z\in \mathcal {R}_S(\mathcal {G})$. Hence, we have $\mathcal {R}_S(\mathcal {G}')\subseteq \mathcal {R}_S(\mathcal {G})$. Moreover, $\mathcal {F}_S(\mathcal {G}')$ does only depend on the (non-)edges of $G_{_{=}}$ and since $G_{_{=}}$ remained unchanged in $\mathcal {G}'$, we have $\mathcal {F}_S(\mathcal {G}') = \mathcal {F}_S(\mathcal {G})$. The latter two arguments together with $(\mathcal {R}_S(\mathcal {G}), \mathcal {F}_S(\mathcal {G}))$ being consistent imply that $(\mathcal {R}_S(\mathcal {G}'), \mathcal {F}_S(\mathcal {G}'))$ is also consistent.

In summary, $G_{_{<}}'$ and $G_{_{=}}$ are properly colored, $G_{_{<}}'$ and $G_{_{>}}'$ are cographs, and $(\mathcal {R}_S(\mathcal {G}'), \mathcal {F}_S(\mathcal {G}'))$ is consistent. Theorem 10 therefore implies that $\mathcal {G}'$ is explained by a relaxed scenario $\mathcal {S}'$. $\square$

### Corollary 10

If $(G_{_{=}},\sigma )$ is an EDT graph, then it is explained by a relaxed scenario $\mathcal {S}$ that satisfies $xy\in E(G_{_{>}}(\mathcal {S}))$ for all $x,y\in L$ that are contained in distinct connected components of $G_{_{=}}$.

Let us now turn the general case of EDT-Recognition. We show that it is NP-hard by reducing from a problem of deciding whether there is a tree that displays a given set of fan triples and a suitable choice of rooted triples. The precise problem statement requires some definitions. Let *U* be a set. Let $C_F$ be a set of fan triples whose leaves are in *U*, and let $C_R$ be a set of unordered pairs of rooted triples of the form $\{{x}{y}\vert {z},{x}{z}\vert {y}\}$ with $x,y,z \in U$. We say that a tree $S^*$ on the leaf set *U* *satisfies* $(C_F, C_R)$ if the following holds:

- For each $x\vert y\vert z \in C_F$, $S^*$ displays $x\vert y\vert z$;
- For each $\{{x}{y}\vert {z},{x}{z}\vert {y}\} \in C_R$, $S^*$ displays either $xy\vert z$ or $xz\vert y$.

This suggests the following decision problem.

### Problem 2

($$(C_F,C_R)$$-Satisfiability)

**Table:** 

*Input:*	A tuple $(U, C_F, C_R)$ where *U* is a set, $C_F$ is a set of fan triples and
	$C_R$ is a set of pairs of rooted triples of the form $\{{x}{y}\vert {z},{x}{z}\vert {y}\}$.
*Question:*	Does there exist a tree $S^*$ on leaf set *U* that satisfies $(C_F, C_R)$?

Jansson et al. [42] showed that a slightly different version of $(C_F,C_R)$-Satisfiability, known as $(F^{+-})$-Consistency, is NP-hard. In the $(F^{+-})$-Consistency problem the input are two sets $F^+$ and $F^-$ of fan triples and one asks for a tree that displays all fan triples in $F^+$ but none of the ones in $F^-$. The latter is equivalent to asking for a tree that that displays all fan triples in $F^+$ and that displays for every $x\vert y\vert z\in F^-$ exactly one of the triples $xy\vert z$, $xz\vert y$, or $yz\vert x$. This translated to a slightly different version of $(C_F, C_R)$-Satisfiability by requiring (i) the elements of $C_R$ to be of the form $\{xy\vert z, xz\vert y, yz\vert x\}$ and (ii) that one of the three triples must be displayed by the final tree. For our purposes, we must restrict $C_R$ to pairs of triples instead of triple sets of size 3. The NP-hardness proof in [42] can be adapted to establish the following result:

### Theorem 15

$(C_F,C_R)$-Satisfiability is NP-complete.

### *Proof*

See Appendix. $$\square $$

Theorem 15, in turn, can be used to prove

### Theorem 16

EDT-Recognition is NP-complete. Moreover, it remains NP-complete if the input graph $(G,\sigma )$ is a cograph.

### *Proof*

See Appendix. $$\square $$

## Explanation of PDT graphs by relaxed scenarios

If only the information of $G_{_{<}}\in \mathcal {G}$ is available, it can be tested whether $G_{_{<}}$ is an LDT graph and, in the affirmative case, a relaxed scenario that explains $G_{_{<}}$ can be constructed in polynomial-time [4]. In contrast, we have seen above that the problem of recognizing an EDT graph is NP-hard (Theorem 16). This begs the question whether recognition of PDT graphs is an easy or hard task.

### Theorem 17

A graph $(G,\sigma )$ is a PDT graph if and only if the following conditions are satisfied:

1. *G* is a cograph, and
2. $({\overline{G}},\sigma )$ is properly colored, and
3. The set of triples $R(G) {:=}\{\sigma (x)\sigma (y)\vert \sigma (z) :xy \in E(G) \text { and } xz, yz \notin E(G) \text { and } \sigma (x),\sigma (y),\sigma (z) \text { are pairwise distinct}\}$ is consistent.

In particular, it can be verified if $(G,\sigma )$ is a PDT graph and, in the affirmative, a scenario that explains $(G,\sigma )$ can be constructed in polynomial time.

### *Proof*

Suppose that $(G,\sigma )$ is a PDT graph. Hence, there is a relaxed scenario $\mathcal {S}$ such that $G=G_{_{>}}(\mathcal {S})$. By Lemma 15, *G* must be a cograph. Since $G=G_{_{>}}(\mathcal {S})$, its complement ${\overline{G}}$ comprises all edges of $G_{_{=}}(\mathcal {S})$ and $G_{_{<}}(\mathcal {S})$. By Cor. 1, $G_{_{=}}(\mathcal {S})$ and $G_{_{<}}(\mathcal {S})$ are always properly colored and so $({\overline{G}},\sigma )$ is also properly colored. The set *R*(*G*) is precisely the set of triples as specified in Def. 6(b’) and, in particular, $R(G)\subseteq \mathcal {R}_S(\mathcal {G})$ where $\mathcal {G}=(G_{_{<}}(\mathcal {S}), G_{_{=}}(\mathcal {S}), G_{_{>}}(\mathcal {S}), \sigma )$. By Theorem 10, $(\mathcal {R}_S(\mathcal {G}), \mathcal {F}_S(\mathcal {G}))$ is consistent, an thus in particular *R*(*G*) is consistent.

Conversely, assume that $(G,\sigma )$ satisfies Conditions (1), (2) and (3). Consider $\mathcal {G}=(G_{_{<}}, G_{_{=}}, G_{_{>}}, \sigma )$ such that $G_{_{>}}= G$, $G_{_{=}}=(V(G),\emptyset )$ and $G_{_{<}}= {\overline{G}}$. Since *G* is a cograph and $G_{_{<}}= {\overline{G}}$, Prop. 1 implies that $G_{_{<}}$ is a cograph. Moreover, by Condition (2), $(G_{_{<}},\sigma )$ is a properly colored cograph. Since there are no edges in $G_{_{=}}$, it follows that $G_{_{=}}$ is also a properly colored cograph. Since $G_{_{=}}$ is edge-less, we have $\mathcal {F}_S(\mathcal {G}) = \emptyset$. Moreover, since *G* is the complement of $G_{_{<}}$, Def. 6(b’) and the definition of *R*(*G*) imply $R(G) = \mathcal {R}_S(\mathcal {G})$. Condition (3) now implies that $\mathcal {R}_S(\mathcal {G})$ is consistent. Together with $\mathcal {F}_S(\mathcal {G}) = \emptyset$ this implies that $(\mathcal {R}_S(\mathcal {G}), \mathcal {F}_S(\mathcal {G}))$ is consistent. Hence, all conditions of Theorem 10 are satisfied and we conclude that there is a relaxed scenario that explains $\mathcal {G}=(G_{_{<}}, G_{_{=}}, G_{_{>}}, \sigma )$. In particular, $G=G_{_{>}}$ is a PDT graph. Re-using the arguments in the proof of Lemma 27, we can construct a scenario for $\mathcal {G}=(G_{_{<}}, G_{_{=}}, G_{_{>}}, \sigma )$ (and thus for $G=G_{_{>}}$ in $O(\ell ^4 \log \ell )$ where $\ell =\max (\vert L \vert , \vert \sigma (L) \vert )$. $\square$

We note that PDT graphs can be recognized faster than the construction of an explaining scenario with the help of Theorem 17. Cographs can be recognized in $O(\vert V\vert +\vert E\vert )$ time [33] and $O(\vert V\vert ^2)$ operations are sufficient to verify that the complement of *G* is properly colored. The triple set *R*(*G*) contains at most $O(\vert \sigma (V)\vert ^3)$ triples which can be constructed in $O(\vert V\vert ^3)$ time. The Aho et al. algorithm checks triple consistency in $O(\vert R\vert \,\vert V\vert )$ time. Hence, PDT graphs can be recognized in $O( \vert V\vert (\vert V\vert ^2 + \vert \sigma (V)\vert ^3))$ time.

## Orthology and quasi-orthology

Most of the mathematical results concerning orthology have been obtained in an HGT-free setting. There, a pair of genes *x* and *y* is orthologous if their last common ancestor ${{\,\textrm{lca}\,}}_T(x,y)$ coincides with the last common ancestor of the two species in which they reside [1]. Thus, we expect a close connection between orthology and the graph $G_{_{=}}(\mathcal {S})$. Thm. 14 in the previous section, furthermore, is reminiscent of the characterization of orthology graphs that can be reconciled with species trees in HGT-free duplication/loss scenarios [18, 19]. We therefore close this contribution by connecting the graph $G_{_{=}}(\mathcal {S})$ with different notions of orthology in scenarios with HGT that have been discussed in the literature.

Disagreements on the “correct” definition of orthology in the presence of HGT stem for the fact that, in general, pairs of genes originating from a speciation event may be separated by HGT, and thus become xenologs. They may even eventually reside in the same species and therefore appear as paralogs. Choanozoa, for example, have two CCA-adding enzymes, one vertically inherited through the eukaryotic lineage, the other horizontally acquired from a bacterial lineage [43]. To accommodate such differences, Darby et al. [8] proposed a classification of subtypes of xenology and, in line with [1], reserve the terms *ortholog* and *paralog* to situations in which the path between *x* and *y* does not contain an HGT event. In this section, we briefly survey notions of orthology that have “natural” definitions in the setting of relaxed scenarios and explore their mathematical properties and their relationships with EDT graphs.

### Definition 18

Let $\mathcal {S}=(T,S,\sigma ,\mu ,\tau _{T},\tau _{S})$ be a relaxed scenario. Two distinct vertices $x,y\in L(T)$ are *weak quasi-orthologs* if $\mu ({{\,\textrm{lca}\,}}_T(x,y))\in V^0(S)$.

Def. 18 is, in essence, Walter Fitch’s original, purely event-based definition of orthology [6]. The graph $\Psi ^{w}(\mathcal {S})$ with vertex set *L*(*T*) and the weak quasi-orthologous pairs as its edges is the *weak quasi-orthology graph* of $\mathcal {S}$.

In later work, Walter M. Fitch [1] emphasizes the condition that “the common ancestor lies in the cenancestor (i.e., the most recent common ancestor) of the taxa from which the two sequences were obtained”, which translates to the following notion:

### Definition 19

Let $\mathcal {S}=(T,S,\sigma ,\mu ,\tau _{T},\tau _{S})$ be a relaxed scenario. Then two distinct genes $x,y\in L(T)$ are *strict quasi-orthologs* if $\mu ({{\,\textrm{lca}\,}}_T(x,y))={{\,\textrm{lca}\,}}_S(\sigma (x),\sigma (y))$.

The graph $\Psi ^{s}(\mathcal {S})$ with vertex set *L*(*T*) and the strict quasi-orthologous pairs as its edges is the *strict quasi-orthology graph* of $\mathcal {S}$. By Obs. 4, all edges of $G_{_{=}}$ form strictly quasi-orthologous pairs in the scenarios produced by Algorithm 1.

Later definitions explicitly exclude xenologs [1, 7]. Translating the concept of orthology used by Darby et al. [8] to our notation yields

### Definition 20

Let $\mathcal {S}=(T,S,\sigma ,\mu ,\tau _{T},\tau _{S})$ be a relaxed scenario. Two distinct vertices $x,y\in L(T)$ are *weak orthologs* if $\mu ({{\,\textrm{lca}\,}}_T(x,y))\in V^0(S)$ and $\lambda (e)=0$ for all edges *e* along the path between *x* and *y* in *T*.

The graph $\Theta ^{w}(\mathcal {S})$ with vertex set *L*(*T*) and the pairs of weak orthologs as its edges will be called the *weak orthology graph* of $\mathcal {S}$. The most restrictive notion of orthology is obtained by enforcing both the matching of last common ancestors and the exclusion of horizontal transfer:

### Definition 21

Let $\mathcal {S}=(T,S,\sigma ,\mu ,\tau _{T},\tau _{S})$ be a relaxed scenario. Two distinct vertices $x,y\in L(T)$ are *strict orthologs* if $\mu ({{\,\textrm{lca}\,}}_T(x,y)) = {{\,\textrm{lca}\,}}_S(\sigma (x), \sigma (y))$ and $\lambda (e)=0$ for all edges *e* along the path between *x* and *y* in *T*.

The graph $\Theta ^{s}(\mathcal {S})$ with vertex set *L*(*T*) and the pairs of (strict) orthologs as its edges will be called the *(strict) orthology graph* of $\mathcal {S}$. We note that strict orthologs also appear in the definition of property (S6): A relaxed scenario satisfies (S6) if and only if $\mu (u)\in V^0(S)$ implies that there is a pair of strict orthologs *x* and *y* with ${{\,\textrm{lca}\,}}_T(x,y)=u$. The alternative notions of orthology and the proposed terminology are summarized in Table 1.

**Table 1:** Summary of the alternative notions of orthology in the presence of HGT events

Reconciliation condition	HGT irrelevant	HGT excluded
$\mu ({{\,\textrm{lca}\,}}_T(x,y)) \in V^0(S)$	$\Psi ^{w}(\mathcal {S})$	$\Theta ^{w}(\mathcal {S})$
	Weak quasi-ortholog	Weak ortholog
$\mu ({{\,\textrm{lca}\,}}_T(x,y)) = {{\,\textrm{lca}\,}}_S(\sigma (x), \sigma (y))$	$\Psi ^{s}(\mathcal {S})$	$\Theta ^{s}(\mathcal {S})$
	Strict quasi-ortholog	(Strict) ortholog

From $\mu ({{\,\textrm{lca}\,}}_T(x,y))={{\,\textrm{lca}\,}}_S(\sigma (x),\sigma (y))$, we obtain $\mu ({{\,\textrm{lca}\,}}_T(x,y))\in V(S)$. Furthermore, if *x* and *y* are distinct, then ${{\,\textrm{lca}\,}}_T(x,y)$ is not a leaf and (S1) in the definition of relaxed scenarios implies that $\mu ({{\,\textrm{lca}\,}}_T(x,y))$ is also not a leaf. Hence we have:

### Observation 6

If $x,y\in L$ are distinct and $\mu ({{\,\textrm{lca}\,}}_T(x,y))={{\,\textrm{lca}\,}}_S(\sigma (x),\sigma (y))$, then $\mu ({{\,\textrm{lca}\,}}_T(x,y))\in V^0(S)$ for every relaxed scenario $\mathcal {S}=(T,S,\sigma ,\mu ,\tau _{T},\tau _{S})$.

As an immediate consequence, every strict quasi-ortholog is a weak quasi-ortholog and every strict ortholog is a weak ortholog. Furthermore strict or weak orthologs are strict or weak quasi-orthologs, respectively. In terms of the corresponding graphs, we therefore have the following subgraph relations:

2 $$\begin{aligned} \Theta ^{s}(\mathcal {S})\subseteq \Psi ^{s}(\mathcal {S}), \qquad \Theta ^{w}(\mathcal {S})\subseteq \Psi ^{w}(\mathcal {S}), \qquad \Psi ^{s}(\mathcal {S})\subseteq \Psi ^{w}(\mathcal {S}), \qquad \Theta ^{s}(\mathcal {S})\subseteq \Theta ^{w}(\mathcal {S}). \end{aligned}$$

That is, we have $\Theta ^{s}(\mathcal {S})\subseteq \Psi ^{s}(\mathcal {S})\subseteq \Psi ^{w}(\mathcal {S})$ and $\Theta ^{s}(\mathcal {S})\subseteq \Theta ^{w}(\mathcal {S})\subseteq \Psi ^{w}(\mathcal {S})$, while $\Psi ^{s}(\mathcal {S})$ and $\Theta ^{w}(\mathcal {S})$ are incomparable w.r.t. the subgraph relation.

### Lemma 34

The weak quasi-orthology graph $\Psi ^{w}(\mathcal {S})$ and the weak orthology graph $\Theta ^{w}(\mathcal {S})$ are cographs for every relaxed scenario $\mathcal {S}$.

### *Proof*

Let $\mathcal {S}=(T,S,\sigma ,\mu ,\tau _{T},\tau _{S})$ be a relaxed scenario. Consider the labeling $t:V^0(T)\rightarrow \{0,1\}$ with $t(u)=1$ iff $\mu (u)\in V^0(S)$. We have $xy\in E(\Psi ^{w}(\mathcal {S}))$ if and only if $t({{\,\textrm{lca}\,}}_T(x,y))=1$. Thus (*T*, *t*) is a cotree that explains $\Psi ^{w}(\mathcal {S})$. By Prop. 1, $\Psi ^{w}(\mathcal {S})$ is a cograph.

Consider (*T*, *t*) and remove all HGT-edges from *T* to obtain the forest $(T^*,t)$. Although the tree(s) in $(T^*,t)$ are not necessarily phylogenetic, we can obtain a cograph *G* with edges $xy\in E(G)$ precisely if *x*, *y* are leaves of a connected component of $(T^*,t)$ and $t({{\,\textrm{lca}\,}}_{T^*}(x,y))=1$. One easily verifies that any two leaves *x* and *y* in a connected component of $T^*$ satisfy ${{\,\textrm{lca}\,}}_{T^*}(x,y)={{\,\textrm{lca}\,}}_T(x,y)$. Therefore, $xy\in E(G)$ precisely if the path connecting *x* and *y* in *T* does not contain an HGT edge and $t({{\,\textrm{lca}\,}}_T(x,y))=1$ (or, equivalently $\mu (u)\in V^0(S)$). Consequently, $G=\Theta ^{w}(\mathcal {S})$ and thus, $\Theta ^{w}(\mathcal {S})$ is a cograph. $\square$

It is worth noting that $xy\in E(\Psi ^{w}(\mathcal {S}))$ does not imply $\sigma (x)\ne \sigma (y)$, i.e., $(\Psi ^{w}(\mathcal {S}),\sigma )$ is not necessarily properly colored. The genes *a* and $a'$ in Fig. 3 serve as an example. Now consider the two relaxed scenarios $\mathcal {S}$ as shown in Fig. 6. In both cases, one observes that $G_{_{=}}(\mathcal {S}) = \Psi ^{s}(\mathcal {S})$. In each case, $G_{_{=}}(\mathcal {S})$ contains an induced $P_4$. Therefore, we obtain

### Observation 7

In general, $\Psi ^{s}(\mathcal {S})$ is not a cograph.

### Lemma 35

The strict orthology graph $\Theta ^{s}(\mathcal {S})$ is a cograph for every relaxed scenario $\mathcal {S}$.

### *Proof*

Let $\mathcal {S}=(T,S,\sigma ,\mu ,\tau _{T},\tau _{S})$ be a relaxed scenario. Note that $\Theta ^{s}(\mathcal {S})\subseteq \Theta ^{w}(\mathcal {S})$. Furthermore, if $xx'\in E(\Theta ^{w}(\mathcal {S}))$, then *x* and $x'$ are leaves in the same subtree of the forest *F*(*T*) obtained by removing all HGT edges from *T*, i.e., *x* and $x'$ are witnesses of ${{\,\textrm{lca}\,}}_T(x,x')$. By definition, we have $\Theta ^{s}(\mathcal {S})\ne \Theta ^{w}(\mathcal {S})$ if and only if there are two vertices $x,x'\in L(T)$ with $\mu ({{\,\textrm{lca}\,}}_T(x,x'))\in V^0(S)$ but $\mu ({{\,\textrm{lca}\,}}_T(x,x'))\ne {{\,\textrm{lca}\,}}_S(\sigma (x),\sigma (x'))$, and there is no HGT-edge on the path between *x* and $x'$ in *T*. Note that the latter condition is equivalent to *x* and $x'$ being witnesses of ${{\,\textrm{lca}\,}}_T(x,x')$. In this case, $xx'\in E(\Theta ^{w}(\mathcal {S}))$ but $xx'\notin E(\Theta ^{s}(\mathcal {S}))$ and Lemma 6 implies ${{\,\textrm{lca}\,}}_S(\sigma (x),\sigma (x'))\prec _S \mu ({{\,\textrm{lca}\,}}_T(x,x'))$. In the following, set $p{:=}{{\,\textrm{lca}\,}}_T(x,x')$, $w{:=}\mu (p)$, $\Theta ^{s}{:=}\Theta ^{s}(\mathcal {S})$ and $\Theta ^{w}{:=}\Theta ^{w}(\mathcal {S})$.

We proceed by modifying $(T,\tau _{T})$ and the reconciliation map $\mu$ to obtain a scenario $\mathcal {S}' = (T',S,\sigma ,\mu ',\tau _{T}',\tau _{S})$ such that $\Theta ^{s}=\Theta ^{s}(\mathcal {S}')$ remains unchanged and the edge $xx'$ is removed from $\Theta ^{w}$. This, in particular, ensures that $\Theta ^{s}\subseteq \Theta ^{w}(\mathcal {S}')\subsetneq \Theta ^{w}$ holds.

Since ${{\,\textrm{lca}\,}}_S(\sigma (x),\sigma (x'))\prec _S w = \mu (p)$, and both *x* and $x'$ are witnesses of *p*, there is a unique child $w^*\in {{\,\textrm{child}\,}}_S(w)$ such that ${{\,\textrm{lca}\,}}_S(\sigma (x),\sigma (x'))\preceq _S w^*$. For this vertex $w^*$, let $A^*\subseteq {{\,\textrm{child}\,}}_T(p)$ be the subset of all children *q* of *p* that satisfy (i) *q* has a witness and (ii) for every witness *y* of *q* holds $\sigma (y)\in L(S(w^*))$. By construction, the unique children $q_x$ and $q_{x'}$ of *p* that satisfy $x\preceq _T q_x$ and $x'\preceq _T q_{x'}$ are contained in $A^*$, i.e., $A^*\ne \emptyset$. Moreover, for any two distinct $q_1,q_2\in A^*$ and all $x_1\in L(T(q_1))$ and $x_2\in L(T(q_2))$ such that $x_1$ is a witness of $q_1$ and $x_2$ is a witness $q_2$, we have ${{\,\textrm{lca}\,}}_S(\sigma (x_1),\sigma (x_2))\preceq _S w^*$. Note that *pq* cannot be an HGT-edge of *T* for all $q\in A^*$, since incomparability of $\mu (p)$ and $\mu (q)$ would imply that at least one edge *uv* along the path from *q* to its witness $x_q$ must satisfy that $\mu (u)$ and $\mu (v)$ are incomparable (otherwise, condition (ii) in the construction of $A^*$ is is not possible). Thus, if $pq\in E(T)$ is an HGT edge for some $q\in {{\,\textrm{child}\,}}_T(p)$, then $q\notin A^*$.

Now construct a modified gene tree $$T'$$ as follows: If $$A^*= {{\,\textrm{child}\,}}_T(p)$$ we set $$T'=T$$ and relabel *p* as $$p^*$$. Otherwise, we insert an additional vertex $$p^*$$ into *T* that has *p* as its parent and the vertices $$q_i\in A^*$$, $$1\le i\le \vert A^*\vert $$ as its children. Note that by construction $$w^*$$ has at least 2 children. The time map for the modified tree is set by $$\tau _{T'}(v)=\tau _{T}(v)$$, $$v\in V(T)$$, and $$\tau _{T'}(p^*)=\tau _{T}(p)-\epsilon $$ for sufficiently small $$\epsilon >0$$. Since we started with a relaxed scenario that explains $$\Theta ^{s}$$, $$T'$$ remains a phylogenetic tree. Moreover, we define the modified reconciliation $$\mu '$$ by setting $$\mu (p^*)=ww^*\in E(S)$$ and $$\mu '(v)=\mu (v)$$ for all $$v\in V(T'){\setminus }\{p^*\}$$ and set $$\mathcal {S}'{:=}(T',S,\mu ',\sigma ,\tau _{T'},\tau _{S})$$. By construction, $${{\,\textrm{lca}\,}}_{T'}(x,x')=p^*$$ and thus, $$\mu (p^*)\in E(S)$$ implies $$xx'\notin E(\Theta ^{w}(\mathcal {S}'))$$. Furthermore, if $${{\,\textrm{lca}\,}}_T(y_1,y_2)=p$$, $$y_1\in L(T(q_1))$$ for some $$q_1\in A^*$$ and $$y_2\in L(T(q_2))$$ for some $$q_2\in {{\,\textrm{child}\,}}_T(p){\setminus } A^*$$, then $${{\,\textrm{lca}\,}}_{T'}(y_1,y_2)=p$$ because $$y_2$$ is not a descendant of $$p^*$$ in $$\mathcal {S}'$$. Finally, if $${{\,\textrm{lca}\,}}_{T}(y_1,y_2)\ne p$$, then $${{\,\textrm{lca}\,}}_{T'}(y_1,y_2)={{\,\textrm{lca}\,}}_{T}(y_1,y_2)$$. The latter two arguments together with the fact that the reconciliation maps for *T* and $$T'$$ coincide for all vertices distinct from $$p^*$$ imply $$\Theta ^{s}(\mathcal {S}')=\Theta ^{s}$$. Furthermore, $$x_1x_2\in E(\Theta ^{w}(\mathcal {S}'))$$ if and only if $$x_1x_2\in E(\Theta ^{w})$$ and $${{\,\textrm{lca}\,}}_{T'}(x_1,x_2)\ne p^*$$. In particular, $$\vert E(\Theta ^{w}(\mathcal {S}')\vert < \vert E(\Theta ^{w})\vert $$. The modification of $$\mathcal {S}$$ also preserves witnesses: if *x* is a witness of $$v\ne p$$ in $$\mathcal {S}$$ then *x* remains a witness of *v* in $$\mathcal {S}'$$; if *x* is a witness of *p* in $$\mathcal {S}$$ then it is a witness of $$p^*$$ in $$\mathcal {S}'$$ and, since $$pp^*$$ is not a HGT-edge, *x* remains a witness of *p*. Thus $$q\in A^*$$ has a witness *x* that is also a witness of *p* in both $$\mathcal {S}$$ and $$\mathcal {S}'$$, and a witness of $$p^*$$ in $$\mathcal {S}'$$. In particular, therefore, $$p^*q$$ with $$q\in A^*$$ is not an HGT edge. Conversely, if $$pq\in E(T)$$ is an HGT edge in $$\mathcal {S}$$, *pq* is also an HGT edge in $$\mathcal {S}'$$ because $$\mu '(p)=\mu (p)$$ and $$\mu '(q)=\mu (q)$$ and *S* remains unchanged. The latter argument holds for all HGT edges in $$\mathcal {S}$$, resp., $$\mathcal {S}'$$. Therefore, *uv* is an HGT-edge in $$\mathcal {S}$$ if and only if *uv* it an HGT edge in $$\mathcal {S}'$$. In particular, therefore, if the path from $$u\in 
V^0(T)$$ to the leaf $$x\in L(T)$$ is HGT-free in $$\mathcal {S}$$, then it is also HGT-free in $$\mathcal {S}'$$.

Repeating this construction produces a finite sequence of scenarios $\mathcal {S}=\mathcal {S}_0,\mathcal {S}_1,\dots ,\mathcal {S}_k$ with the same strict orthology graphs $\Theta ^{s}=\Theta ^{s}(\mathcal {S}_1)=\dots =\Theta ^{s}(\mathcal {S}_k)$ and in each step strictly reduces the number of edges in the weak orthology graph, i.e., $\Theta ^{w}(\mathcal {S}_{i})\subsetneq \Theta ^{w}(\mathcal {S}_{i-1})$ for $1\le i\le k$ as long as in $\mathcal {S}_{i-1}$ there is a vertex *p* with a set $A^*$ with $\vert A^*\vert \ge 2$. Eventually we arrive at a relaxed scenario $\mathcal {S}_k$ with a refined gene tree $T_k$ that contains no vertex *p* with set $A^*$ as defined above. In $\mathcal {S}_k$, therefore, $w=\mu _k({{\,\textrm{lca}\,}}_{T_k}(x,y))\in V^0$ implies ${{\,\textrm{lca}\,}}_S(\sigma (x),\sigma (y))=w$, which in turn implies $\Theta ^{w}(\mathcal {S}_k)=\Theta ^{s}(\mathcal {S}_k)=\Theta ^{s}$. The assertion now follows since $\Theta ^{w}(\mathcal {S}_k)$ is a cograph by Lemma 34. $\square$

The modification of a relaxed scenario $\mathcal {S}$ in the proof of Lemma 35 only affects the last common ancestors of pairs of genes $x,x'$ with $\mu ({{\,\textrm{lca}\,}}_T(x,x;))\succ _S {{\,\textrm{lca}\,}}_S(\sigma (x),\sigma (x'))$ and thus $xy\in E(G_{_{<}})$. Furthermore, in the modified scenario $\mathcal {S}'$, by construction we still have $\mu ({{\,\textrm{lca}\,}}_{T'}(x,x;))\succ _S {{\,\textrm{lca}\,}}_S(\sigma (x),\sigma (x'))$, since either ${{\,\textrm{lca}\,}}_{T'}(x,x')={{\,\textrm{lca}\,}}_{T}(x,x')$ or $\tau _T({{\,\textrm{lca}\,}}_{T'}(x,x'))=\tau _T({{\,\textrm{lca}\,}}_{T'}(x,x'))-\epsilon$ for an arbitrarily small $\epsilon$. Therefore, we have $\mathcal {G}(\mathcal {S})=\mathcal {G}(\mathcal {S}')$ in each step, which immediately implies

### Proposition 5

A graph 3-partition $\mathcal {G}$ is explained by a relaxed scenario if and only if it is explained by a relaxed scenario satisfying $\Theta ^{s}(\mathcal {S})=\Theta ^{w}(\mathcal {S})$.

Finally, we show that every valid input $\mathcal {G}=(G_{_{<}}, G_{_{=}}, G_{_{>}}, \sigma )$ has an explanation such that the EDT graph $G_{_{=}}$ represents the strict quasi-orthologs. This explanation can, in particular, by obtained with Alg. 1. To see this, we first provide

### Lemma 36

Let $\mathcal {S}$ be a relaxed scenario. Then $\Psi ^{s}(\mathcal {S})\subseteq G_{_{=}}(\mathcal {S})$.

### *Proof*

Assume that $xy\in E(\Psi ^{s}(\mathcal {S}))$. Thus we have $x\ne y$ and $\mu ({{\,\textrm{lca}\,}}_T(x,y))={{\,\textrm{lca}\,}}_S(\sigma (x), \sigma (y))\in V(S)$, which in turn yields $\tau _{S}(\mu ({{\,\textrm{lca}\,}}_T(x,y))) = \tau _{S}({{\,\textrm{lca}\,}}_S(\sigma (x), \sigma (y)))$. Together with (S2), this implies that $\tau _{S}({{\,\textrm{lca}\,}}_S(\sigma (x), \sigma (y))) = \tau _{T}({{\,\textrm{lca}\,}}_T(x,y))$ and, therefore, $xy\in E(G_{_{=}}(\mathcal {S}))$. Hence, we have $\Psi ^{s}(\mathcal {S})\subseteq G_{_{=}}(\mathcal {S})$. $\square$

### Lemma 37

If $\mathcal {S}$ is a scenario produced by Algorithm 1 to explain the valid input $\mathcal {G}=(G_{_{<}},G_{_{=}},G_{_{>}},\sigma )$, then $G_{_{=}}=\Psi ^{s}(\mathcal {S})$.

### *Proof*

Obs. 4 implies that $G_{_{=}}\subseteq \Psi ^{s}(\mathcal {S})$ for every scenario $\mathcal {S}$ produced by Algorithm 1. Conversely, every scenario $\mathcal {S}$ produced by Algorithm 1 with input $\mathcal {G}=(G_{_{<}},G_{_{=}},G_{_{>}},\sigma )$ is relaxed (cf. Lemma 26) and satisfies, in particular, $G_{_{=}}= G_{_{=}}(\mathcal {S})$. Hence, we can apply Lemma 36 to conclude that $\Psi ^{s}(\mathcal {S})\subseteq G_{_{=}}(\mathcal {S}) = G_{_{=}}$. $\square$

It is important to note, however, that there are scenarios for which $G_{_{=}}\subseteq \Psi ^{s}(\mathcal {S})$ is not true. As an example, consider the scenario $\mathcal {S}$ in Fig. 1(top row, middle) in which $xy\in G_{_{=}}(\mathcal {S})$ but $\mu ({{\,\textrm{lca}\,}}_T(x,y))\ne {{\,\textrm{lca}\,}}_S(\sigma (x),\sigma (y))$ and thus, $xy\notin \Psi ^{s}(\mathcal {S})$.

*Generic Scenarios.* It will sometimes be useful to assume that time maps are generic in the sense that two inner vertices of the gene or species tree have the same time stamp only if they belong to the same biological event. For our purposes, it seems sufficient to rule out that concurrent nodes are mapped to different positions in the species tree, i.e., we postulate the following “genericity” axiom for evolutionary scenarios:

(G) If $\tau _{T}(v)=\tau _{S}(U)$ for $v\in V^0(T)$ and $U\in V^0(S)$, then $\mu (v)=U$.

Axiom (G) stipulates that no two distinct speciation *events*, i.e., inner nodes of the species tree are concurrent and that no other evolutionary event (duplication or horizontal transfer) happens concurrent with a speciation. Note that two vertices of the gene tree “belong” to the same speciation event if they are reconciled with the same vertex of *S*. Thus $u,u'\in V(T)$ with $\mu (u)=\mu (u')\in V(S)$ are considered as the same speciation event and thus also necessarily have the same time stamp $\tau _{T}(u)=\tau _{T}(u')$.

As an immediate consequence of (G), we observe that $\tau _{T}({{\,\textrm{lca}\,}}_T(x,y))=\tau _{S}(U)$ implies $\mu ({{\,\textrm{lca}\,}}_T(x,y))=U$. Conversely, since *T* is phylogenetic, every $v\in V^0(T)$ (except the planted root) is the last common ancestor of some pair of vertices, and $\mu (0_T)=0_S$, we can equivalently express (G) as

(G’) If $\tau _{T}({{\,\textrm{lca}\,}}_T(x,y))=\tau _{S}(U)$ for $x,y\in L(T)$ and $U\in V^0(S)$, then $\mu ({{\,\textrm{lca}\,}}_T(x,y))=U$.

### Definition 22

A relaxed scenario satisfying (G), or equivalently (G’), is called *generic*.

We note in passing that it is not a trivial endeavor to modify a relaxed scenario $\mathcal {S}$ to a generic one $\mathcal {S}'$ such that $\mathcal {G}(\mathcal {S}) = \mathcal {G}(\mathcal {S}')$. Simply adjusting the time maps is, in general, not enough. For example, consider scenario $\mathcal {S}_3=(T,S,\sigma ,\mu ,\tau _{T},\tau _{S})$ in Fig. 13(C). Without adjusting the reconciliation map $\mu$, any generic scenario $\mathcal {S}'=(T,S,\sigma ,\mu ,\tau _{T}',\tau _{S}')$ would satisfy $ab\notin E(G_{_{=}}(\mathcal {S}'))$ although $ab\in E(G_{_{=}}(\mathcal {S}_3))$. Hence, additional effort is needed to adjust $\mu$, i.e., to map ${{\,\textrm{lca}\,}}_T(a,b)$ to ${{\,\textrm{lca}\,}}_S(\sigma (a),\sigma (b))$ instead of mapping it to the edge $\rho _S\sigma (c)$. However, for every scenario $\mathcal {S}$, there exists a (possibly alternative) scenario $\mathcal {S}'$ that is computed using $\mathcal {G}(\mathcal {S})$ as input for Algorithm 1 in conjunction with Algorithm 2. Therefore, $\mathcal {S}'$ satisfies $\mathcal {G}(\mathcal {S}') = \mathcal {G}(\mathcal {S})$ and the conditions provided in Observation 4 and 5. These strong constraints on $\mathcal {S}'$ might be helpful in transforming it into a generic scenario.

### Theorem 23

For a generic scenario $\mathcal {S}=(T,S,\sigma ,\mu ,\tau _{T},\tau _{S})$ it always holds that $G_{_{=}}(\mathcal {S}) = \Psi ^{s}(\mathcal {S})$ and thus, $G_{_{=}}(\mathcal {S})\subseteq \Psi ^{w}(\mathcal {S})$. In particular, if $\mathcal {S}$ is HGT-free or *S* and *T* are binary, then $\Psi ^{s}(\mathcal {S})$ is a cograph.

### *Proof*

Let $\mathcal {S}=(T,S,\sigma ,\mu ,\tau _{T},\tau _{S})$ be a generic scenario. Assume first that $xy\in E(G_{_{=}}(\mathcal {S}))$. By definition, $x\ne y$ and $\tau _{T}({{\,\textrm{lca}\,}}_T(x,y))=\tau _{S}({{\,\textrm{lca}\,}}_S(\sigma (x),\sigma (y)))$. By (G’), $\mu ({{\,\textrm{lca}\,}}_T(x,y))={{\,\textrm{lca}\,}}_S(\sigma (x),\sigma (y))$. Hence, $xy\in E(\Psi ^{s}(\mathcal {S}))$ and, therefore, $G_{_{=}}(\mathcal {S})\subseteq \Psi ^{s}(\mathcal {S})$.

By Lemma 36, we have $\Psi ^{s}(\mathcal {S})\subseteq G_{_{=}}(\mathcal {S})$ and, thus, $\Psi ^{s}(\mathcal {S}) = G_{_{=}}(\mathcal {S})$. By Equ. (2), we have $G_{_{=}}(\mathcal {S}) = \Psi ^{s}(\mathcal {S})\subseteq \Psi ^{w}(\mathcal {S})$. Moreover, $\Psi ^{s}(\mathcal {S}) = G_{_{=}}(\mathcal {S})$ together with Lemma 21 and Theorem 7 implies that $\Psi ^{s}(\mathcal {S})$ is a cograph whenever $\mathcal {S}$ is HGT-free or *S* and *T* are binary. $\square$

**Fig. 12:**
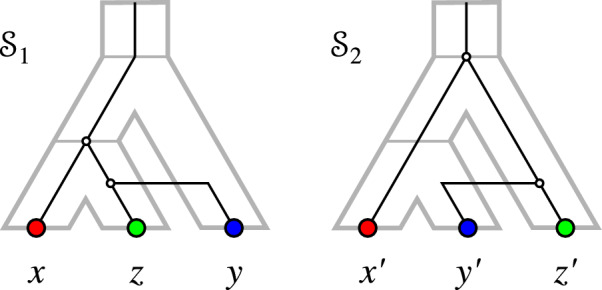
The two pairs *x* and *y* as well as $x'$ and $y'$ are weak quasi-orthologs in $\mathcal {S}_1=(T,S,\sigma ,\mu ,\tau _{T},\tau _{S})$ and $\mathcal {S}_2=(T',S',\sigma ',\mu ',\tau _{T'},\tau _{S'})$, respectively, but it holds $\tau _{T}({{\,\textrm{lca}\,}}_T(x,y)) < \tau _{S}({{\,\textrm{lca}\,}}_S(\sigma (x),\sigma (y))$ and $\tau _{T'}({{\,\textrm{lca}\,}}_{T'}(x',y')) > \tau _{S'}({{\,\textrm{lca}\,}}_{S'}(\sigma '(x'),\sigma '(y'))$

Note that a pair of weak quasi-orthologs $x,y\in L(T)$ may have arisen in a speciation and have been transferred to the species $\sigma (x)$ and $\sigma (y)$ in which they are found at later points in time. Thus $\tau _{T}({{\,\textrm{lca}\,}}_T(x,y)) \lessgtr \tau _{S}({{\,\textrm{lca}\,}}_S(\sigma (x),\sigma (y))$ is possible, see Fig. 12 for two examples. Consequently, $\Psi ^{w}(\mathcal {S}) \ne G_{_{=}}(\mathcal {S})$ is possible for generic scenarios.

As an immediate consequence of Lemma 6, equality between $\Psi ^{w}(\mathcal {S})$ and $G_{_{=}}(\mathcal {S})$ also holds for HGT-free scenarios. In particular, by definition, $\Theta ^{s}(\mathcal {S}) = \Psi ^{s}(\mathcal {S})$. Hence, together with Lemma 21, we obtain

### Corollary 11

Every relaxed scenario $\mathcal {S}$ without HGT-edges satisfies $G_{_{=}}(\mathcal {S})=\Psi ^{s}(\mathcal {S})=\Theta ^{s}(\mathcal {S})$. In this case, $\Psi ^{s}(\mathcal {S})$ is a cograph.

### Corollary 12

Let $\mathcal {S}$ be a generic scenario. Then $G_{_{=}}(\mathcal {S})=\Psi ^{w}(\mathcal {S})$ if and only if $\mu ({{\,\textrm{lca}\,}}_T(x,y))={{\,\textrm{lca}\,}}_S(\sigma (x),\sigma (y))$ for all $xy\in E(\Psi ^{w}(\mathcal {S}))$, which holds if and only if $\Psi ^{s}(\mathcal {S})=\Psi ^{w}(\mathcal {S})$. In this case, $\Psi ^{s}(\mathcal {S})$ is a cograph.

**Fig. 13:**
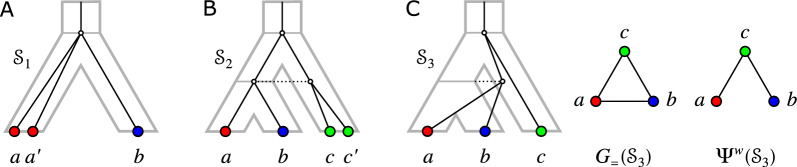
**A** A HGT-free relaxed scenario where $G_{_{=}}(\mathcal {S}_1)\subsetneq \Psi ^{w}(\mathcal {S}_1)$. The vertices *a* and $a'$ are weak quasi-orthologs but $aa'\notin E(G_{_{=}}(\mathcal {S}_1))$. **B** An HGT-free, non-generic relaxed scenario. **C** A non-generic relaxed scenario for which $G_{_{=}}(\mathcal {S}_3)\ne \Psi ^{w}(\mathcal {S}_3)$ even though $\mu ({{\,\textrm{lca}\,}}_T(x,y))={{\,\textrm{lca}\,}}_S(\sigma (x),\sigma (y))$ holds for all $xy\in E(\Psi ^{w}(\mathcal {S}_3))$

The example in Fig. 13C show that the condition (G) cannot be dropped in Cor. 12.

Equ. 2 and Thm. 23 immediately imply

### Corollary 13

Every generic scenario $\mathcal {S}$ satisfies $\Theta ^{s}(\mathcal {S}) \subseteq \Psi ^{s}(\mathcal {S}) = G_{_{=}}(\mathcal {S})\subseteq \Psi ^{w}(\mathcal {S})$.

## Concluding remarks

We have developed a complete characterization of graph 3-partition $\mathcal {G}$ on a species-colored set of vertices that can be explained by an relaxed scenario $\mathcal {S}$ (Thm. 10). We showed, furthermore, that whenever such an explaining relaxed scenario exists, one can also find explanations from a much more restricted class of scenarios that are fully witnessed and satisfy certain natural constraints for “speciation events” (Thm. 13). The existence of such scenarios can be tested in polynomial time, and in the positive case, both relaxed and restricted scenarios explaining the input 3-partition can be constructed, again in polynomial time. If only the information of $G_{_{=}}\in \mathcal {G}$ is available, it can be tested in polynomial-time as whether $G_{_{=}}$ is an EDT graph in the HGT-free case (cf. Thm. 14), while the problem becomes NP-hard for general relaxed scenarios (cf. Thm. 16). In contrast, PDT graphs can be recognized in polynomial-time (cf. Thm. 17). These approaches extend earlier work on LDT graphs, which serve as the basis for indirect methods for the inference of HGT events [4]. If only the information of $G_{_{<}}\in \mathcal {G}$ is available, it can be tested whether $G_{_{<}}$ is an LDT graph and, in the affirmative case, a relaxed scenario that explains $G_{_{<}}$ can be constructed in polynomial-time [4].

Relaxed scenarios also can be used to formalize Walter Fitch’s concept of xenologous gene pairs [1, 8]. Given a relaxed scenario $\mathcal {S}=(T,S,\sigma ,\mu ,\tau _{T},\tau _{S})$, we define the *xenology relation* *R* by setting $(x,y)\in R$ precisely if $x,y\in L(T)$ and the unique path connecting *x* and *y* in *T* contains an HGT edge. The resulting graph ${{\,\mathrm{\digamma }\,}}(\mathcal {S}) {:=}(L(T), R)$ is known as *symmetrized Fitch graph* [45–47]. It is always a properly colored multipartite graph. Thm. 5 in [4] shows that for every properly colored multipartite graph there is a relaxed scenario $\mathcal {S}$ such that $G_{_{<}}(\mathcal {S}) = {{\,\mathrm{\digamma }\,}}(\mathcal {S})$. On the other hand, by [4, Thm. 4], the LDT graph $G_{_{<}}(\mathcal {S})$ is always a subgraph of ${{\,\mathrm{\digamma }\,}}(\mathcal {S})$ for every relaxed scenario $\mathcal {S}$. Thus, for every $\mathcal {S}$ and every $xy\in G_{_{<}}(\mathcal {S})$, the two genes *x* and *y* are separated by at least on HGT-event. There are examples of relaxed scenarios $\mathcal {S}$ for which $G_{_{<}}(\mathcal {S})\ne {{\,\mathrm{\digamma }\,}}(\mathcal {S})$ (cf. [4, Fig. 7]). Whether $G_{_{<}}(\mathcal {S}) \subsetneq {{\,\mathrm{\digamma }\,}}(\mathcal {S})$ or $G_{_{<}}(\mathcal {S}) = {{\,\mathrm{\digamma }\,}}(\mathcal {S})$ heavily depends on the particular scenario $\mathcal {S}$. Given $\mathcal {G}= (G_{_{<}},G_{_{=}},G_{_{>}},\sigma )$, which may be estimated empirically from sequence similarity data, an explaining scenario $\mathcal {S}$ is not uniquely determined in general. This begs the question whether there is a relaxed scenario $\mathcal {S}$ that explains $\mathcal {G}$ and satisfies $G_{_{<}}={{\,\mathrm{\digamma }\,}}(\mathcal {S})$. To see that this is not the case, consider $\mathcal {G}{:=}\mathcal {G}(\mathcal {S}_2)$, where $\mathcal {S}_2$ is the scenario as in Fig. 4. In this case, $G_{_{<}}$ is not a complete multipartite graph and thus, $G_{_{<}}\subsetneq {{\,\mathrm{\digamma }\,}}(\mathcal {S})$ for every relaxed scenario $\mathcal {S}$ that explains $G_{_{<}}$. Consequently, the information on HGT-events is not always provided entirely by the knowledge of $G_{_{<}}$ alone. The graphs $G_{_{=}}$ and $G_{_{>}}$ thus may add additional information for the inference of HGT. It will therefore be an interesting topic for future work to understand how to employ $\mathcal {G}=(G_{_{<}},G_{_{=}},G_{_{>}},\sigma )$ to detect HGT-events and to which extend HGT-events are uniquely determined for a given $\mathcal {G}$.

Relaxed scenarios provide a very general framework in which the concepts of orthology, paralogy, and xenology can be studied in a rigorous manner. In Section “Orthology and Quasi-Orthology”, we compared different concepts of orthology that have been proposed for situations with horizontal transfer. We obtained simple results describing the mutual relationships of the corresponding variants of “orthology graphs” on *L*(*T*), and their relations with $G_{_{=}}$. With the exception of the strict quasi-orthology graph $\Psi ^{w}(\mathcal {S})$, the alternative notions lead to colored cographs similar to the HGT-free case, see [21]. The latter connections are of practical importance since the EDT graph $G_{_{=}}$, or the 3-partition graphs, can be estimated from sequence similarities. It will be interesting, therefore, to explore if techniques similar to those employed by Schaller et al. [48] can be used to identify the edges on $G_{_{=}}$ that do not correspond to orthology-relationships.

We found that, similar to LDT graphs, PDT graphs are also cographs. This is in general not the case for EDT graphs, although EDT graphs are perfect (Prop. 4). If both gene tree and species tree are binary, i.e., fully resolved, then the EDT graph is a cograph. However, not all proper vertex colorings of a cograph result in an EDT graph (Fig. 8). It remains an interesting open problem to characterize the “EDT-colorings” of cographs in analogy to the hc-colorings of cograph that appear in the context of reciprocal best match graphs [49, 50]. Moreover, it is at least of theoretical interest to ask how difficult it is to decide whether a suitable coloring $\sigma$ exists such that $(G_{_{<}},G_{_{=}},G_{_{>}},\sigma )$ is explained by a relaxed scenario. Finding such a coloring corresponds to assigning species to genes, a problem that arises in metagenomics. Indeed, when DNA is extracted from bulk samples taken from the environment, the species that contains each sequence is unknown since they belong to members of a diverse population (for instance, microbial or fungal). Popular techniques to recover a species assignment include sequence similarity analysis [51] and phylogenetic reconstructions [52]. Since our approaches combine these two ideas, it will be interesting to see whether EDT-colorings can be useful in the context of metagenomics.

The reconciliation of *T* and *S* implicitly determines what kind of evolutionary event corresponds to a vertex $v\in V^0(T)$. Given a relaxed or restricted scenario $\mathcal {S}$, the assignment of an event label $t(v)\in Q$ from some pre-defined set *Q* of event types is, of course, a matter of biological interpretation of $\mathcal {S}$. The definitions of “DTL scenarios” as in [16, 53, 54] assign event labels to the inner vertices of *T* that then must satisfy certain consistency conditions with the local behavior of the reconciliation map $\mu$. Event labelings $t:V^0(T)\rightarrow Q$ also play a key role in orthology detection in duplication/loss scenarios [18, 19, 48, 55]. In relaxed scenarios, it is not always possible to assign event types that match with straightforward biological interpretations in an unambiguous manner. For example, from a biological perspective, *speciation events* are usually defined as “passing on the entire ancestral genome to each offspring lineage”. In Fig. 3, however, ${{\,\textrm{lca}\,}}_T(a,a')$ describes a gene duplication that occurs together with the speciation event. As noted in [3, Fig.2], this issue already arises in the setting of DL-scenarios with multifurcating trees even in HGT-free scenarios that satisfy the speciation constraint S6, see also [2]. Some further pertinent results on event-based reconciliation in the presence of HGT were discussed by Nøjgaard et al. [38]. These point out subtle differences for non-binary species trees in the definition of event-based DTL-scenarios [16] and suggest a natural notion of event-annotated relaxed scenarios. Because of these difficulties we have avoided to consider event types as a formal level in this contribution. Instead, these issues will be the focus of a forthcoming contribution.

It is reassuring that a graph 3-partition $\mathcal {G}$ that can be explained by a relaxed scenario can always also be explained by a restricted scenario. This begs the question, however, whether there is a simple, local editing algorithm that converts a “true” scenario in a restricted or at least a fully witnessed one. In the case of HGT-free scenarios, there is a simple rule to exclude “non-observable” vertices in *T*: in this restricted setting, it suffices to recursively remove all deleted genes and all inner vertices with a single child [18]. The situation seems to be much less obvious for relaxed scenarios, since these models are somewhat more general than “event-driven” scenarios. For instance, relaxed scenarios allow multiple descendants from nodes $v\in V(T)$ with $\mu (v)\in V(S)$. As a consequence, is seems difficult to interpret a vertex *v* that is reconciled with a vertex in the species tree as a “speciation event” in the strict sense. The exact meaning of “events”, therefore, deserves a more detailed analysis in the setting of relaxed scenarios.

## Figures and Tables

**Fig. 14:**
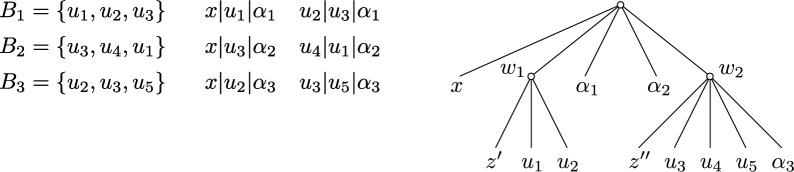
An example instance of Set-Splitting with solution $U_1 = \{u_1, u_2\}, U_2 = \{u_3, u_4, u_5\}$. The elements of $B_1, B_2, B_3$ are in the order chosen by the reduction. The constructed tree $S^*$ is shown, along with the $x\vert b_j^1\vert \alpha _j$ and $b_j^2\vert b_j^3\vert \alpha _j$ fan triples that must be displayed. Note that each of the three cases in which two elements of $B_j$ have the same parent occurs

**Fig. 15:**
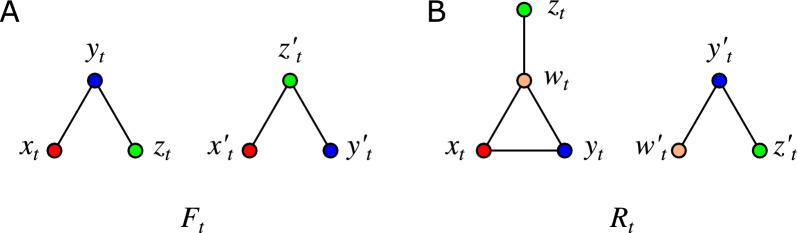
The graphs $F_t$ and $R_t$ as constructed in the proof of Theorem 16

## Data Availability

Not applicable.

## Appendix

### Proof of Lemma 23

In this section, we show in detail that, given a valid input $\mathcal {G}=(G_{_{<}},G_{_{=}},G_{_{>}},\sigma )$ with vertex set *L*, Algorithm 1 indeed returns a relaxed scenario $\mathcal {S}=(T,S,\sigma ,\mu ,\tau _{T},\tau _{S})$ such that $L(T)=L$. The proof parallels the arguments in the proof of Thm. 2 in [4].

#### Proof of Lemma 23

Let $\sigma :L\rightarrow M$ and set $\mathcal {R}=\mathcal {R}_S(\mathcal {G})$ and $\mathcal {F}=\mathcal {F}_S(\mathcal {G})$. By a slight abuse of notation, we will simply write $\mu$ and $\tau _{T}$ also for restrictions to subsets of *V*(*T*). By assumption, $(\mathcal {R},\mathcal {F})$ is consistent, and thus, a tree *S* on *M* that displays $\mathfrak {S}$ exists, and can be constructed in Line 1 e.g. using MTT [34]. By Lemma 1, we can always construct a time map $\tau _{S}$ for *S* satisfying $\tau _{S}(x)=0$ for all $x\in L(S)$ in Line 2. By definition, $\tau _{S}(y)>\tau _{S}(x)$ must hold for every edge $yx\in E(S)$, and thus, we obtain $\epsilon >0$ in Line 3.

Recall that $\sigma (L')\subseteq L(S(u_S))$ holds in every recursion step by Obs. 2 and note that we reach the *else*-block starting in Line 13 only if $u_S$ is not a leaf. Therefore, the auxiliary graphs $H_1$, $H_2$, and $H_3$ are well-defined and there is a vertex $v^*_S\in {{\,\textrm{child}\,}}_S(u_S)$ such that $\sigma (C_j)\cap L(S(v^*_S))\ne \emptyset$ for every connected component $C_j$ of $H_2$ in Line 19, and a vertex $v_S\in {{\,\textrm{child}\,}}_S(u_{S})$ such that $\sigma (C_k)\subseteq L(S(v_S))$ for every connected component $C_k$ of $H_3$ in Line 22. Moreover, ${{\,\textrm{par}\,}}_S(u_{S})$ is always defined since we have $u_S=\rho _S$ and thus ${{\,\textrm{par}\,}}_S(u_S)=0_S$ in the top-level recursion step, and recursively call the function BuildGeneTree on vertices $v_S$ such that $v_S\prec _S u_S$.

In summary, all assignments are well-defined in every recursion step. It is easy to verify that the algorithm terminates since, in each recursion step, we either have that $u_S$ is a leaf, or we recurse on vertices $v_{S}$ that lie strictly below $u_S$. We argue that the resulting tree $T'$ is a (not necessarily phylogenetic) tree on *L* by observing that, in each step, each $x\in L'$ is either attached to the tree as a leaf (if $u_S$ is a leaf) or passed down to a recursion step on some connected component of $H_3$ since each connected component $C_k$ of $H_3$ satisfies $C_k\subseteq C_j$ for some connected component $C_j$ of $H_2$ which in turn satisfies $C_j\subseteq C_i$ for some connected component $C_i$ of $H_1$. Nevertheless, $T'$ is turned into a phylogenetic tree *T* by suppression of degree-two vertices in Line 26. Finally, $\mu (x)$ and $\tau _{T}(x)$ are assigned for all vertices $x\in L(T')=L$ in Line 11, and for all newly created inner vertices in Lines 7, 16, and 20.

Before we continue to show that $\mathcal {S}$ is a relaxed scenario, we first show that the conditions for time maps and time consistency are satisfied for $(T', S, \sigma , \mu , \tau _{T}, \tau _{S})$:

#### Claim 1

For all $x,y \in V(T')$ with $x\prec _{T'} y$, we have $\tau _{T}(x)<\tau _{T}(y)$. Moreover, for all $x\in V(T')$, the following statements are true:

(i) if $\mu (x)\in V(S)$, then $\tau _{T}(x)=\tau _{S}(\mu (x))$, and
(ii) if $\mu (x)=(a,b)\in E(S)$, then $\tau _{S}(b)<\tau _{T}(x)<\tau _{S}(a)$.

#### Proof of Claim

Recall that we always write an edge *uv* of a tree *T* such that $v\prec _T u$. For the first part of the statement, it suffices to show that $\tau _{T}(x)<\tau _{T}(y)$ holds for every edge $yx\in E(T')$, and thus to consider all vertices $x\ne \rho _{T'}$ in $T'$ and their unique parent, which will be denoted by *y* in the following. Likewise, we have to consider all vertices $x\in V(T')$ including the root to show the second statement. The root $\rho _{T'}$ of $T'$ corresponds to the vertex $\rho '$ created in Line 6 in the top-level recursion step on *L* and $\rho _{S}$. Hence, we have $\mu (\rho _{T'}) = {{\,\textrm{par}\,}}_S(\rho _S)\rho _S = 0_S\rho _S \in E(S)$ and $\tau _{T}(\rho _{T'})=\tau _{S}(\rho _S) +\epsilon$ (cf. Line 7). Therefore, we have to show Subcase (ii). Since $\epsilon >0$, it holds that $\tau _{S}(\rho _S)<\tau _{T}(\rho _{T'})$. Moreover, $\tau _{S}(0_S)-\tau _{S}(\rho _{S})\ge 3\epsilon$ holds by construction, and thus $\tau _{S}(0_S)-(\tau _{T}(\rho _{T'})-\epsilon )\ge 3\epsilon$ and $\tau _{S}(0_S)-\tau _{T}(\rho _{T'})\ge 2\epsilon$, which together with $\epsilon >0$ implies $\tau _{T}(\rho _{T'})<\tau _{S}(0_S)$.

We now consider the remaining vertices $x\in V(T'){\setminus }\{\rho _{T'}\}$. Every such vertex *x* is introduced into $T'$ in some recursion step on $L'$ and $u_S$ in exactly one of the following four ways:

- $x\in L(T')$ is a leaf attached to some inner vertex $\rho '$ in Line 10,
- $x=u_i$ is created in Line 15,
- $x=v_j$ is created in Line 18, and
- $x=w_k{:=}\texttt {BuildGeneTree}(C_k,v_S)$ is attached to the tree in Line 23.

Note that if $x=\rho '$ is created in Line 6, then $\rho '$ is either the root of $T'$, or equals a vertex $w_k{:=}\texttt {BuildGeneTree}(C_k,v_S)$ that is attached to the tree in Line 23 in the “parental” recursion step.

In Case (a), we have that $x\in L(T')$ is a leaf and attached to some inner vertex $y=\rho '$. Since $u_S$ must be a leaf in this case, and thus $\tau _{S}(u_S)=0$, we have $\tau _{T}(y)=0+\epsilon =\epsilon$ and $\tau _{T}(x)=0$ (cf. Lines 7 and 11). Since $\epsilon >0$, this implies $\tau _{T}(x)<\tau _{T}(y)$. Moreover, we have $\mu (x)=\sigma (x)\in L(S)\subset V(S)$ (cf. Line 11), and thus have to show Subcase (i). Since $u_S$ is a leaf and $\sigma (L')\subseteq L(S(u_S))$, we conclude $\sigma (x)=u_S$. Thus we obtain $\tau _{T}(x)=0=\tau _{S}(u_S)=\tau _{S}(\mu (x))$.

In Case (b), we have that $x=u_i$ is created in Line 15 and attached as a child to some vertex $y=\rho '$ created in the same recursion step. Thus, we have $\tau _{T}(y)=\tau _{S}(u_S)+\epsilon$, $\tau _{T}(x)=\tau _{S}(u_S)$ and $\mu (x)=u_S\in V(S)$ (cf. Lines 7 and 16). Therefore and because$\epsilon >0$, it holds $\tau _{T}(x)<\tau _{T}(y)$ and Subcase (i) is satisfied.

In Case (c), we have that $x=v_j$ is created in Line 18 and attached as a child to some vertex $y=u_i$ created in the same recursion step. Thus, we have $\tau _{T}(y)=\tau _{S}(u_S)$ and $\tau _{T}(x)=\tau _{S}(u_S)-\epsilon$ (cf. Lines 16 and 20). Therefore and since $\epsilon >0$, it holds $\tau _{T}(x)<\tau _{T}(y)$. Moreover, we have $\mu (x)=u_S v^*_S \in E(S)$ for some $v^*_S\in {{\,\textrm{child}\,}}_S(u_S)$. Hence, we have to show Subcase (ii). By a similar calculation as before, $\epsilon >0$, $\tau _{S}(u_S)-\tau _{S}(v^*_S)\ge 3\epsilon$ and $\tau _{T}(x)=\tau _{S}(u_S)-\epsilon$ imply $\tau _{S}(v^*_S)<\tau _{T}(x)<\tau _{S}(u_S)$.

In Case (d), $x=w_k{:=}\texttt {BuildGeneTree}(C_k,v_S)$ is attached to the tree in Line 23 and equals $\rho '$ as created in Line 6 in some “child” recursion step with $v_S\in {{\,\textrm{child}\,}}_S(u_S)$. Thus, we have $\tau _{T}(x)=\tau _{S}(v_S)+\epsilon$ and $\mu (x)=u_S v_S \in E(S)$ (cf. Line 7). Moreover, *x* is attached as a child of some vertex $y=v_j$ as created in Line 18. Thus, we have $\tau _{T}(y)=\tau _{S}(u_S)-\epsilon$. By construction and since $u_S v_S \in E(S)$, we have $\tau _{S}(u_S)-\tau _{S}(v_S)\ge 3\epsilon$. Therefore, $(\tau _{T}(y)+\epsilon ) - (\tau _{T}(x)-\epsilon ) \ge 3\epsilon$ and thus $\tau _{T}(y)- \tau _{T}(x) \ge \epsilon$. This together with $\epsilon >0$ implies $\tau _{T}(x)<\tau _{T}(y)$. Moreover, since $\mu (x)=u_S v_S \in E(S)$ for some $v_S\in {{\,\textrm{child}\,}}_S(u_S)$, we have to show Subcase (ii). By a similar calculation as before, $\epsilon >0$, $\tau _{S}(u_S)-\tau _{S}(v_S)\ge 3\epsilon$ and $\tau _{T}(x)=\tau _{S}(v_S)+\epsilon$ imply $\tau _{S}(v_S)<\tau _{T}(x)<\tau _{S}(u_S)$. $\square$

The tree *T* is obtained from $T'$ by first adding a planted root $0_T$ (and connecting it to the original root) and then suppressing all inner vertices except $0_T$ that have only a single child in Line 26. In particular, *T* is a planted phylogenetic tree by construction. The root constraint (S0) $\mu (x)=0_S$ if and only if $x=0_T$ also holds by construction (cf. Line 27). Since we clearly have not contracted any outer edges (*y*, *x*), i.e. with $x\in L(T')$, we conclude that $L(T')=L(T)=L$. As argued before, we have $\tau _{T}(x)=0$ and $\mu (x)=\sigma (x)$ whenever $x\in L(T')=L(T)$ (cf. Line 11). Since, in addition, all other vertices are mapped by $\mu$ to some edge of *S*, inner vertex, or $0_S$ (cf. Lines 7, 16, 20, and 27), the leaf constraint (S1) is satisfied.

By construction, we have $V(T)\setminus \{0_T\} \subseteq V(T')$. Moreover, suppression of vertices clearly preserves the $\preceq$-relation between all vertices $x,y\in V(T){\setminus } \{0_T\}$. Together with Claim 1, this implies $\tau _{T}(x)<\tau _{T}(y)$ for all vertices $x,y\in V(T){\setminus } \{0_T\}$ with $x\prec _{T} y$. For the single child $\rho _T$ of $0_T$ in *T*, we have $\tau _{T}(\rho _T)\le \tau _{S}(\rho _S)+\epsilon$ where equality holds if the root of $T'$ was not suppressed and thus is equal to $\rho _T$. Moreover, $\tau _{T}(0_T)=\tau _{S}(0_S)$ and $\tau _{S}(0_S)-\tau _{S}(\rho _S)\ge 3\epsilon$ hold by construction. Taken together the latter two arguments imply that $\tau _{T}(\rho _T)<\tau _{T}(0_T)$. In particular, we obtain $\tau _{T}(x)<\tau _{T}(y)$ for all vertices $x,y\in V(T)$ with $x\prec _{T} y$. Hence, $\tau _{T}$ is a time map for *T*, which, moreover, satisfies $\tau _{T}(x)=0$ for all $x\in L(T)$.

To show that $\mathcal {S}=(T,S,\sigma ,\mu , \tau _{T},\tau _{S})$ is a relaxed scenario, it remains to show the two time consistency constraints (S2) and (S3) in Def. 2. For $0_T$, we have $\tau _{T}(0_T)=\tau _{S}(0_S)=\tau _{S}(\mu (0_T))$. Hence, condition in (S2) is satisfied for $0_T$. The remaining vertices of *T* are all vertices of $T'$ as well. The latter two arguments together with Claim 1 imply that conditions (S2) and (S3) are also satisfied, and thus $\mathcal {S}$ is a relaxed scenario. $\square$

### Hardness of EDT graph recognition

To establish the NP-hardness of $(C_F,C_R)$-Satisfiability and EDT-Recognition, we start from

#### Problem 3

(3-Set Splitting)

**Table:**

*Input:*	A finite set *U* and a collection $B=\{B_1,\dots ,B_m\}$ of subsets of *U*
	s.t. $\vert B_i\vert =3$ for all *i*.
*Question:*	Is there a partition $\{U_1, U_2\}$ of *U* into two sets such that, for each $B_j \in B$,
	we have $B_j\cap U_1\ne \emptyset$ and $B_j\cap U_2\ne \emptyset$.
	In other words, none of the $B_j \in B$ is entirely contained in either $U_1$ or $U_2$.

Lovász [56] showed that the “unrestricted” version of 3-Set Splitting, in which elements in $B_j \in B$ have size $\vert B_i\vert \le 3$ instead of $\vert B_i\vert = 3$, is NP-complete. There does not seem to be a published proof for the NP-completeness of the “restricted” variant of 3-Set Splitting. For completeness, we include a simple argument starting from

#### Problem 4

(monotone NAE-3-SAT)

**Table:**

*Input:*	Given a set of clauses $C = \{C_1, \dots , C_m\}$ over a set *U* of Boolean variables
	s.t. $\vert C_i\vert =3$ for all *i* and $C_i$ contains no negated variables.
*Question:*	Is there a truth assignment to *U* such that in each $C_i$
	not all three literals are set to true?

As shown by Porschen et al. [57, Thm. 3], monotone NAE-3-SAT is NP-complete. Its is straightforward to see that monotone NAE-3-SAT and 3-Set Splitting are equivalent in the following sense: Interpret the $C_i\in C$ as sets and put $B=C$. Then (*C*, *U*) is a yes-instance of monotone NAE-3-SAT if and only if (*B*, *U*) is a yes-instance of 3-Set Splitting because we can obtain a solution $\{U_1, U_2\}$ for (*B*, *U*) from a solution for (*C*, *U*) by setting $U_1{:=}\{x\in U \mid x \text { is true}\}$ and $U_2{:=}U\setminus U_1$. Conversely, a solution for (*C*, *U*) is obtained from a solution $\{U_1, U_2\}$ for (*B*, *U*) by assigning “true” exactly to all $x\in U_1$. Consequently, we have

#### Proposition 6

3-Set Splitting is NP-complete.

We are now in the position to prove NP-completeness of $(C_F,C_R)$-Satisfiability (Thm. 15).

#### Proof of Theorem 15

Given a tree $S^*$, it can be verified in polynomial-time as whether $S^*$ satisfies $(C_F, C_R)$. Hence, $(C_F,C_R)$-Satisfiability$\in \text {NP}$. To show NP-hardness we use a reduction from 3-Set Splitting.

Given an instance (*U*, *B*) of 3-Set Splitting, construct an instance $(U', C_F, C_R)$ of $(C_F, C_R)$-Satisfiability as follows. For $B_j \in B$, we order its three elements arbitrarily and write $B_j = \{b_j^1, b_j^2, b_j^3\}$. Let $U' {:=}U \cup \{x, z', z''\} \cup \{\alpha _j: 1 \le j \le m\}$ and let

$$\begin{aligned} C_F{:=}&\{x\vert z'\vert z''\} \cup \bigcup _{j=1}^m \{\ x \vert b_j^1 \vert \alpha _j,\ b_j^2 \vert b_j^3 \vert \alpha _j\ \}, \\ C_R{:=}&\{\ \{{u_i}{z'}\vert {z''},{u_i}{z''}\vert {z'}\} : u_i \in U\ \} \end{aligned}$$

It is easy to verify that this reduction can be performed in polynomial time. We show that there exists a 3-set splitting of *B* if and only if there exists a tree $S^*$ that satisfies $(C_F, C_R)$.

Assume first that (*U*, *B*) is a yes-instance of 3-Set Splitting, i.e., there is a partition $\{U_1, U_2\}$ of *U* such that $\vert B_j \cap U_1\vert \in \{1, 2\}$ for each $B_j \in B$. We construct a tree $S^*$ that satisfies $(C_F, C_R)$, see Fig. 14 for an illustrative example. Start with $S^*$ as the tree in which the root has three children $x, w_1, w_2$. Then, add each element of $\{z'\} \cup U_1$ as a child of $w_1$, and add each element of $\{z''\} \cup U_2$ as a child of $w_2$. Notice that $S^*$ displays $x\vert z'\vert z''$ as required by $C_F$. Moreover, because each $u_i$ has either $z'$ or $z''$ as a sibling but not both, $S^*$ displays either $u_i z' \vert z''$ or $u_i z'' \vert z'$ for each $u_i \in U$, and thus satisfies the constraints in $C_R$. We next add the remaining $\alpha _j$ leaves as children of existing vertices of $S^*$, which cannot alter the triples and fan triples gathered so far.

For each $$B_j \in B$$, exactly two of $$b_j^1, b_j^2$$ and $$ b_j^3$$ have the same parent $$w \in \{w_1, w_2\}$$ in $$S^*$$, because $$\{U_1, U_2\}$$ is a 3-set splitting. There are three cases, and in each one, we let the reader verify that $$S^*$$ displays $$x\vert b_j^1\vert \alpha _j$$ and $$b_j^2\vert b_j^3\vert \alpha _j$$:

if either $b_j^1$ and $b_j^2$ or $b_j^1$ and $b_j^3$ have the same parent *w*, then add $\alpha _j$ as a child of the root of $S^*$;

if $b_j^2$ and $b_j^3$ have the same parent *w*, then add $\alpha _j$ as a child of *w*.

It is now straightforward to verify that $S^*$ satisfies $(C_F,C_R)$.

Suppose now that $(U', C_F, C_R)$ is a yes-instance of $(C_F,C_R)$-Satisfiability, i.e., there exists a tree $S^*$ that satisfies $(C_F, C_R)$. By the construction of $C_R$, for $u_i \in U$, $S^*$ displays either $u_i z' \vert z''$ or $u_i z'' \vert z'$. We claim that the partition $\{U_1, U_2\}$ where

$$\begin{aligned} U_1 {:=}&\{u_i : S^* \text{ displays } u_i z' \vert z'' \} \text { and}\\ U_2 {:=}&\{u_i : S^* \text{ displays } u_i z'' \vert z' \} \end{aligned}$$

is a 3-set splitting of *B*. In fact, $U_1\cap U_2=\emptyset$, since $S^*$ cannot display both $u_i z' \vert z''$ and $u_i z'' \vert z'$ at the same time. Moreover, by construction of $C_R$ and since $S^*$ satisfies $(C_F, C_R)$, at least one of the triples $u_i z' \vert z''$ and $u_i z'' \vert z'$ must be displayed by $S^*$ for all $u_i\in U$. Consequently, $U_1\cup U_2=U$.

Assume, for contradiction, that $\{U_1, U_2\}$ is not a 3-set splitting of *B*. Hence, there is a $B_j = \{b_j^1, b_j^2, b_j^3\}$ in *B* such that either $B_j \subseteq U_1$ or $B_j \subseteq U_2$. First, suppose that $B_j \subseteq U_1$. By construction of $U_1$, $S^*$ displays $b_j^1 z' \vert z''$, $b_j^2 z' \vert z''$, and $b_j^3 z' \vert z''$. Since $S^*$ displays $x\vert z'\vert z'' \in C_F$, we have $r {:=}{{\,\textrm{lca}\,}}_{S^*}(x, z') = {{\,\textrm{lca}\,}}_{S^*}(x, z'') = {{\,\textrm{lca}\,}}_{S^*}(z', z'')$. Let $y'$ be the unique child of *r* such that $z'\preceq _{S^*} y'$, and note that *x* and $z''$ are not descendants of $y'$. Since $S^*$ displays $b_j^1 z' \vert z''$, $b_j^2 z' \vert z''$, and $b_j^3 z' \vert z''$, it follows that $b_j^1$, $b_j^2$, and $b_j^3$ are all descendants of $y'$. Now, $\alpha _j$ cannot be a descendant of $y'$, as otherwise $S^*$ would display $b_j^1 \alpha _j \vert x$, as opposed to the fan triple $x\vert b_j^1\vert \alpha _j \in C_F$ that $S^*$ must display. On the other hand, if $\alpha _j$ is not a descendant of $y'$, then $b^j_2,b^j_3\prec _{S^*} y'$ implies that $S^*$ displays $b_j^2 b_j^3 \vert \alpha _j$, a contradiction since $b_j^2 \vert b_j^3 \vert \alpha _j \in C_F$. Hence, $B_j \subseteq U_1$ is not possible. By interchanging the roles of $z'$ and $z''$ and using similar arguments, one shows that $B_j \subseteq U_2$ is not possible either. In summary, $\{U_1, U_2\}$ is a 3-set splitting. $\square$

We are now in the position to prove NP-completeness of EDT-Recognition (Thm. 16).

#### Proof of Theorem 16

First note that the problem is in NP, since a scenario that explains a given instance $(G, \sigma )$ can easily be verified in polynomial time. We show that EDT-Recognition is NP-hard by reduction from the $(C_F, C_R)$-Satisfiability problem. Let $(U, C_F, C_R)$ be an instance of $(C_F, C_R)$-Satisfiability. We proceed by constructing a corresponding instance $(G, \sigma )$ of EDT-Recognition as the disjoint union of colored graphs $F_t$ for all $t\in C_F$ and $R_t$ for all $t\in C_R$.

The color set $\Sigma$ comprises a distinct color $\sigma (u)$ for each $u\in U$, and a distinct color $\sigma (t)$ for each $t\in C_R$. Note that for each pair of triples $t = \{{x}{y}\vert {z},{x}{z}\vert {y}\} \in C_R$ a single color $\sigma (t)$ is used. Hence, $\Sigma$ contains $\vert U \vert + \vert C_R\vert$ colors.

For each $t {:=}x\vert y\vert z \in C_F$, we define $F_t$ as the vertex colored graph with

- vertex set $$V(F_t){:=}\{x_t,y_t,z_t,x'_t,y'_t,z'_t\}$$,
- edge set $E(F_t) {:=}\{x_ty_t,y_tz_t,x'_tz'_t,z'_ty'_t\}$, and
- vertex coloring $\sigma (x_t)=\sigma (x_t')=\sigma (x)$, $\sigma (y_t)=\sigma (y_t')=\sigma (y)$, and $\sigma (z_t)=\sigma (z_t')=\sigma (z)$.

By construction $F_t$ consists of two connected components, namely the two $P_3$s $x_t-y_t-z_t$ and $x'_t-z'_t-y'_t$ on three colors. In particular, $F_t$ is properly colored. Moreover, $F_t$ and $F_{t'}$ are vertex disjoint for distinct $t,t'\in C_F$ even though *t* and $t'$ may have leaves in common and thus, the vertices in $V(F_t)$ and $V(F_{t'})$ may share colors.

For each $t {:=}\{{x}{y}\vert {z},{x}{z}\vert {y}\} \in C_R$ we define $R_t$ as the vertex colored graph with

- vertex set $$V(R_t) {:=}\{x_t, y_t, z_t, w_t, y_t', z_t', w_t'\}$$,
- edge set $E(R_t) {:=}\{x_t w_t, x_t y_t, w_t y_t, w_t z_t, w_t' y_t', y_t' z_t'\}$, and
- vertex coloring $\sigma (x_t) = \sigma (x)$, $\sigma (y_t) = \sigma (y_t') = \sigma (y)$, $\sigma (z_t) = \sigma (z_t') = \sigma (z)$, and $\sigma (w_t) = \sigma (w_t') = \sigma (t)$.

By construction, $R_t$ consists of two connected components, a so-called *paw graph* on the four vertices $x_t$, $y_t$, $z_t$, and $w_t$ and the $P_3$ $w_t' - y_t' - z_t'$. In particular, $R_t$ is properly colored. Again, $R_t$ and $R_{t'}$ for distinct $t,t'\in C_R$ are vertex disjoint but may share certain colors. Since $C_F\cap C_R= \emptyset$, we have $t\ne t'$ for any $F_t$ and $R_{t'}$, i.e., each *t* unambiguously refer to either a subgraph $F_t$ or a subgraph $R_t$ of $(G,\sigma )$. The graphs $F_t$ and $R_t$ are illustrated in Fig. 15(A) and (B), respectively.

Since $F_t$ and $R_t$ can be constructed in constant time for each $t\in C_F\cup C_R$, the graph $(G,\sigma )$ can be constructed in polynomial time. Every connected component of *G* is either a paw component” or a “$P_3$ component”. By construction, any two vertices that are in the same connected component of $(G,\sigma )$ have different colors. Thus $(G,\sigma )$ is properly colored.

We proceed by showing that there exists a tree $S^*$ that satisfies $(C_F, C_R)$ if and only if there exists a relaxed scenario $\mathcal {S}$ that explains $(G, \sigma )$. As we shall see, $F_t$ ensures that the species tree $S^*$ displays the fan triple $\sigma (x)\vert \sigma (y)\vert \sigma (z)$, while $R_t$ enforces the species tree to display either $\sigma (x)\sigma (y)\vert \sigma (z)$ or $\sigma (x)\sigma (z)\vert \sigma (y)$.

In the following we simplify the notation and denote the color of a vertex *u* in *G* by ${\tilde{u}}$ instead of $\sigma (u)$.

Suppose first that $(G, \sigma )$ is a yes-instance of EDT-Recognition and thus, there exists a relaxed scenario $\mathcal {S}= (T,S,\sigma ,\mu ,\tau _{T},\tau _{S})$ that explains $(G, \sigma )$. We show that there exists a tree $S^*$ that satisfies $(C_F,C_R)$. Consider $\mathcal {G}= (G_{_{<}}(\mathcal {S}), G_{_{=}}(\mathcal {S}), G_{_{>}}(\mathcal {S}), \sigma )$, where by assumption $G_{_{=}}(\mathcal {S}) = G$. By Prop. 2, the species tree *S* of $\mathcal {S}$ agrees with $(\mathcal {R}_S(\mathcal {G}), \mathcal {F}_S(\mathcal {G}))$.

We claim that $S_{\vert \tilde{x}\tilde{y}\tilde{z}}$ coincides with the fan triple $\tilde{x}\vert \tilde{y}\vert \tilde{z}$ for every $t = x \vert y \vert z \in C_F$. To see this, consider the subgraph $F_t$ in *G*. It contains $x_t-y_t-z_t$ and $x'_t - z'_t - y'_t$ as induced $P_3$s. By Definition 6, therefore, $\tilde{x}\tilde{y}\vert \tilde{z}$, $\tilde{x}\tilde{z}\vert \tilde{y}$, and $\tilde{y}\tilde{z}\vert \tilde{x}$ are forbidden triples of $\mathcal {F}_S(\mathcal {G})$, and thus $S_{\vert \tilde{x}\tilde{y}\tilde{z}}$ must display $\tilde{x}\vert \tilde{y}\vert \tilde{z}$ as claimed. We next claim that for each $t = \{{x}{y}\vert {z},{x}{z}\vert {y}\} \in C_R$, $S_{\vert \tilde{x}\tilde{y}\tilde{z}}$ is either $\tilde{x}\tilde{y}\vert \tilde{z}$ or $\tilde{x}\tilde{z}\vert \tilde{y}$. Consider the subgraph $R_t$ in *G*. It contains $w_t' - y_t' - z_t'$ as an induced $P_3$. By Definition 6, therefore, $\tilde{w}\tilde{y}\vert \tilde{z}$ and $\tilde{y}\tilde{z}\vert \tilde{w}$ are forbidden triples of $\mathcal {F}_S(\mathcal {G})$. We argue next that $y_t z_t \in E(G_{_{<}}(\mathcal {S}))$. To this end, suppose for contradiction that $y_t z_t \in E(G_{_{>}}(\mathcal {S}))$. This together with Definition 6 and $w_t y_t, w_tz_t \in E(G)=E(G_{_{=}}(\mathcal {S}))$ implies that $\tilde{y}\tilde{z}\vert \tilde{w}$ is an informative triple of $\mathcal {R}_S(\mathcal {G})$; a contradiction to $\tilde{y}\tilde{z}\vert \tilde{w}$ being a forbidden triple. Together with $y_t z_t \notin E(G)$, this leaves $y_t z_t \in E(G_{_{<}}(\mathcal {S}))$ as the only possibility. Now consider $x_t z_t$, which is not an edge in $G=G_{_{=}}(\mathcal {S})$. We have the two possibilities $x_t z_t \in E(G_{_{<}}(\mathcal {S}))$ and $x_t z_t \in E(G_{_{>}}(\mathcal {S}))$. Again using Definition 6, $x_t z_t, y_t z_t \in E(G_{_{<}}(\mathcal {S}))$ and $x_t y_t \notin E(G_{_{<}}(\mathcal {S}))$ yield the informative triple $\tilde{x}\tilde{y}\vert \tilde{z}$ in the former case; and $x_t z_t \in E(G_{_{>}}(\mathcal {S}))$ and $x_t y_t, y_t z_t \notin E(G_{_{>}}(\mathcal {S}))$ yield the informative triple $\tilde{x}\tilde{z}\vert \tilde{y}$. Hence, in either case, $S_{\vert \tilde{x}\tilde{y}\tilde{z}}$ is either $\tilde{x}\tilde{y}\vert \tilde{z}$ or $\tilde{x}\tilde{z}\vert \tilde{y}$, as claimed.

We now construct a tree $S^*$ that satisfies $(C_F, C_R)$ from *S* as follows. We first set $S' {:=}S_{\vert \{\tilde{u}: u \in U \}}$. In other words, $S'$ is the minimal phylogenetic subtree of *S* that connects all leaves that are distinct from $\tilde{w}_t$ for $t \in C_R$. Moreover, since $w_t$ is not part of any of the aforementioned triples and fan triples, the tree $S'$ still displays, for every $t = x \vert y \vert z \in C_F$, the fan triple $\tilde{x}\vert \tilde{y}\vert \tilde{z}$ and, for every $t = \{{x}{y}\vert {z},{x}{z}\vert {y}\} \in C_R$, either the triple $\tilde{x}\tilde{y}\vert \tilde{z}$ or the triple $\tilde{x}\tilde{z}\vert \tilde{y}$. The tree $S^*$ obtained from $S'$ by relabeling, for each $u\in U$, the leaf $\tilde{u}$ by *u* therefore satisfies $(C_F, C_R)$.

Suppose that $(U, C_F, C_R)$ is a yes-instance of $(C_F,C_R)$-Satisfiability and thus, there exists a tree $S^*$ on leaf set *U* that satisfies $(C_F, C_R)$. We first construct a graph 3-partition $\mathcal {G}= (G_{_{<}}, G_{_{=}}, G_{_{>}}, \sigma )$ and then use Theorem 10 to argue that $\mathcal {G}$ can be explained by some relaxed scenario.

We start by setting $G_{_{=}}{:=}G$ and proceed as follows:

1. for any two distinct connected components $H_1$ and $H_2$ of *G* and any $x \in H_1, y \in H_2$, add *xy* to $E(G_{_{>}})$;
2. for each $t = x\vert y\vert z \in C_F$, add $x_t z_t$ and $x_t' y_t'$ to $E(G_{_{<}})$;
3. for each $t = \{{x}{y}\vert {z},{x}{z}\vert {y}\} \in C_R$, add $y_t z_t$ and $w_t' z_t'$ to $E(G_{_{<}})$ and, for $x_t z_t$, there are two cases:
  - if $S^*$ displays $xy\vert z$, then add $x_t z_t$ to $E(G_{_{<}})$;
  - if $S^*$ displays $xz\vert y$, then add $x_t z_t$ to $E(G_{_{>}})$.
  Note that no other case is possible since $S^*$ satisfies $(C_F,C_R)$.

This completes the construction of $\mathcal {G}$. Since rules (A2) and (A3) assign an edge in either $G_{_{>}}$ or $G_{_{<}}$ to every non-adjacent pair of vertices within the same connected component, i.e., induced $P_3$ or paw graph of $G_{_{=}}$, and rule (A1) covers all edges between these connected components, $\mathcal {G}$ is a graph 3-partition.

#### Claim 2

For each $ab \in E(G_{_{<}})$, *a* and *b* are in the same connected component of *G*. Moreover, the connected components of $G_{_{<}}$ are isolated edges or induced $P_3$s.

#### Proof of Claim 2

Only Steps (A2) and (A3) add edges to $G_{_{<}}$, and they only add edges between vertices of the same $P_3$ or paw component of *G*. Moreover, in each such component, these steps never add more than two edges to $G_{_{<}}$, and so the connected components of $G_{_{<}}$ are isolated edges or induced $P_3$s, as claimed. $\diamond$

#### Claim 3

The graphs $G_{_{<}}$ and $G_{_{=}}$ are properly colored.

#### Proof of Claim 3

Because $G_{_{=}}= G$, the graph $G_{_{=}}$ is properly colored by construction. As for $G_{_{<}}$, the endpoints of $G_{_{<}}$ edges always belong to the same $P_3$ on three colors or $P_4$ on four colors in *G* by Claim 2, and they have a different color by construction. $\diamond$

#### Claim 4

The graphs $G_{_{<}}$ and $G_{_{>}}$ are cographs.

#### Proof of Claim 4

For $G_{_{<}}$, this holds because its connected components have at most 3 vertices by Claim 2 and, thus, it cannot contain an induced $P_4$. Now consider the graph $G_{_{=}}\cup G_{_{<}}$. Since only Steps (A2) and (A3) add edges to $G_{_{<}}$, and they only add edges between vertices of the same $P_3$ or paw component of *G*, the connected components of $G_{_{=}}\cup G_{_{<}}$ all have 3 or 4 vertices. In particular, upon inspection of Fig. 15 and Steps (A2) and (A3), one easily verifies that none of these components contains an induced $P_4$. Therefore, $G_{_{=}}\cup G_{_{<}}$ must be a cograph. Finally, since $\mathcal {G}$ is a graph 3-partition, $G_{_{>}}$ is the complement graph of $G_{_{=}}\cup G_{_{<}}$ and thus also a cograph. $\diamond$

By Theorem 10, it remains to show that $(\mathcal {R}_S(\mathcal {G}), \mathcal {F}_S(\mathcal {G}))$ is consistent. To this end, we construct a species tree *S* that agrees with $(\mathcal {R}_S(\mathcal {G}), \mathcal {F}_S(\mathcal {G}))$. First, we set $S{:=}S^*$ and, for each $u \in U$, relabel the leaf *u* in *S* to $\tilde{u}$. Second, we insert the remaining leaves $\{\tilde{w}_t :t \in C_R\}$ to *S*. To this end, for each $t {:=}\{{x}{y}\vert {z},{x}{z}\vert {y}\} \in C_R$, we add $\tilde{w}_t$ as a child of ${{\,\textrm{lca}\,}}_S(\tilde{y}, \tilde{z})$. We note that if *S* contains a fan triple $\tilde{a}\vert \tilde{b}\vert \tilde{c}$ (resp. rooted triple $\tilde{a}\tilde{b}\vert \tilde{c}$) for $\tilde{a},\tilde{b}, \tilde{c}\in \Sigma$, then after inserting a leaf as a child of an existing vertex of *S*, the tree *S* still displays $\tilde{a}\vert \tilde{b}\vert \tilde{c}$ or $\tilde{a}\tilde{b}\vert \tilde{c}$, respectively. Therefore, each insertion of a leaf $\tilde{w}_t$ preserves the triples and fan triples that are already displayed by *S*.

We continue by showing that *S* agrees with $(\mathcal {R}_S(\mathcal {G}), \mathcal {F}_S(\mathcal {G}))$.

#### Claim 5

The species tree *S* displays every triple in $\mathcal {R}_S(\mathcal {G})$.

#### Proof of Claim 5

Suppose that there are $a,b,c \in V(G)$ that imply an informative triple $\sigma (a) \sigma (b) \vert \sigma (c) \in \mathcal {R}_S(\mathcal {G})$ (we refrain from using *x*, *y*, *z* as in Definition 6 to avoid confusion with the $x_t, y_t, z_t$ vertices). Together with Definition 6, this implies that one of the following two cases holds: (1) $ac, bc \in E(G_{_{<}})$ and $ab\notin E(G_{_{<}})$ or (2) $ab \in E(G_{_{>}})$ and $ac, bc \notin E(G_{_{>}})$.

*Case (1):* $ac, bc \in E(G_{_{<}})$ and $ab \notin E(G_{_{<}})$. By rule (A1), vertices of distinct connected components of *G* are connected by edges in $G_{_{>}}$. Since $ac, bc \in E(G_{_{<}})$, the vertices *a*, *b* and *c* must be contained in the same connected component of *G*. Clearly, each $P_3$ component contains at most one edge in $G_{_{<}}$ (since two of the three possible edges are edges in $G=G_{_{=}}$). Therefore, *a*, *b*, *c* must be part of a paw component belonging to an $R_t$ subgraph, with $t = \{{x}{y}\vert {z},{x}{z}\vert {y}\} \in C_R$. In particular, we must have $a=x_t$, $b=y_t$, and $c=z_t$ (noting that the roles of *a* and *b* are interchangeable). Since $x_t z_t = ac \in E(G_{_{<}})$, $S^*$ must display $xy\vert z$ according to rule (A3) and, thus, *S* displays $\tilde{x}\tilde{y}\vert \tilde{z}= \sigma (a) \sigma (b) \vert \sigma (c)$.

*Case (2):* $ab \in E(G_{_{>}})$ and $ac, bc \notin E(G_{_{>}})$. By rule (A1), vertices of distinct connected components of *G* are connected by edges in $G_{_{>}}$. Since $ac, bc \notin E(G_{_{>}})$, the vertices *a*, *b* and *c* must be contained in the same connected component of *G*. Since we never add $G_{_{>}}$ edges between vertices in a $P_3$ component, *a*, *b*, *c* must be part of a paw component belonging to an $R_t$ subgraph, with $t = \{{x}{y}\vert {z},{x}{z}\vert {y}\} \in C_R$. In particular, we must have $a=x_t$ and $b=z_t$ (again, the roles of *a* and *b* are interchangeable). Since $x_t z_t = ab \in E(G_{_{>}})$, $S^*$ must display $xz\vert y$ according to rule (A3) and, thus, *S* displays $\tilde{x}\tilde{z}\vert \tilde{y}$. By construction of *S*, $\tilde{w}_t$ is a child of ${{\,\textrm{lca}\,}}_S(\tilde{y},\tilde{z})$. Together with *S* displaying $\tilde{x}\tilde{z}\vert \tilde{y}$, this implies that *S* also displays $\tilde{x}\tilde{z}\vert \tilde{w}_t$. For *c*, the two possibilities $c=y_t$ and $c=w_t$ remain, for which we obtain $\sigma (a) \sigma (b) \vert \sigma (c) = \tilde{x}\tilde{z}\vert \tilde{y}$ and $\sigma (a) \sigma (b) \vert \sigma (c) = \tilde{x}\tilde{z}\vert \tilde{w}_t$, respectively. Hence, $\sigma (a) \sigma (b) \vert \sigma (c)$ is displayed by *S* in both cases.

In summary, *S* displays every informative triple of $\mathcal {R}_S(\mathcal {G})$. $\diamond$

#### Claim 6

The species tree *S* does not display any triple in $\mathcal {F}_S(\mathcal {G})$.

#### Proof of Claim 6

Suppose that there are vertices $a,b,c \in V(G)$ that imply a forbidden triple $\sigma (a) \sigma (b) \vert \sigma (c) \in \mathcal {F}_S(\mathcal {G})$. By Definition 6, we have (1) $ab, bc \in E(G_{_{=}})$ and $ac \notin E(G_{_{=}})$ or (2) $ab, ac \in E(G_{_{=}})$ and $bc \notin E(G_{_{=}})$. In the following, we consider only Case (1), since analogous arguments apply in Case (2). Because $ab,bc \notin E(G_{_{>}})$, we know that *a*, *b* and *c* are contained in the same connected component of *G*.

Suppose that *a*, *b*, and *c* are in the same $P_3$ component of some $F_t$ subgraph where $t = x\vert y\vert z \in C_F$. Thus $\{\sigma (a),\sigma (b),\sigma (c)\} = \{\tilde{x},\tilde{y},\tilde{z}\}$. In this case, since $S^*$ contains $x\vert y\vert z$, *S* contains $\tilde{x}\vert \tilde{y}\vert \tilde{z}= \sigma (a) \vert \sigma (b) \vert \sigma (c)$ and thus does not contain the forbidden triple implied by *a*, *b*, *c*.

Suppose that *a*, *b*, and *c* are in the same $P_3$ component of some $R_t$ component where $t = \{{x}{y}\vert {z},{x}{z}\vert {y}\} \in C_R$. Thus $\{\sigma (a),\sigma (b),\sigma (c)\} = \{\tilde{w}_t,\tilde{y},\tilde{z}\}$. Since we have added $\tilde{w}_t$ as a child of ${{\,\textrm{lca}\,}}_S(\tilde{y},\tilde{z})$, *S* contains $\tilde{w}_t\vert \tilde{y}\vert \tilde{z}= \sigma (a) \vert \sigma (b) \vert \sigma (c)$ and thus does not contain the forbidden triple implied by *a*, *b*, *c*.

Finally, suppose that *a*, *b*, and *c* are in the same paw component of some $R_t$ component where $t = \{{x}{y}\vert {z},{x}{z}\vert {y}\} \in C_R$. Then either (i) $a = y_t$, $b = w_t$, $c = z_t$; (ii) $a = z_t$, $b = w_t$, $c = y_t$; (iii) $a = x_t$, $b = w_t$, $c = z_t$; or (iv) $a = z_t$, $b = w_t$, $c = x_t$. In Cases (i) and (ii), we again have $\{\sigma (a),\sigma (b),\sigma (c)\} = \{\tilde{w}_t,\tilde{y},\tilde{z}\}$ and, as argued before, *S* does not contain the forbidden triples implied by *a*, *b*, *c*. Now consider Cases (iii) and (iv), and thus $\sigma (a)\sigma (b) \vert \sigma (c) = \tilde{x}\tilde{w}_t \vert \tilde{z}$ and $\sigma (a)\sigma (b) \vert \sigma (c) = \tilde{z}\tilde{w}_t \vert \tilde{x}$, respectively. Since $S^*$ displays either $xy\vert z$ or $xz\vert y$, *S* displays $\tilde{x}\tilde{y}\vert \tilde{z}$ or $\tilde{x}\tilde{z}\vert \tilde{y}$. Since we have moreover added $\tilde{w}_t$ as a child of ${{\,\textrm{lca}\,}}_S(\tilde{y},\tilde{z})$, *S* displays $\tilde{x}\vert \tilde{z}\vert \tilde{w}_t$ or $\tilde{x}\tilde{z}\vert \tilde{w}_t$, respectively. Hence, *S* displays none of the two forbidden triples obtained in Cases (iii) and (iv).

Taken together, *S* does not display a triple in $\mathcal {F}_S(\mathcal {G})$. $\diamond$

We have constructed the graph 3-partition $\mathcal {G}= (G_{_{<}},G_{_{=}},G_{_{>}},\sigma )$ such that $G_{_{<}}$ and $G_{_{=}}$ are properly colored by Claim 3, $G_{_{<}}$ and $G_{_{>}}$ are cographs by Claim 4, and $(\mathcal {R}_S(\mathcal {G}),\mathcal {F}_S(\mathcal {G}))$ is consistent by Claim 5 and Claim 6. By Theorem 10, this implies that $\mathcal {G}$ can be explained by a relaxed scenario $\mathcal {S}$. Since $G_{_{=}}(\mathcal {S}) = G_{_{=}}= G$, we can conclude that *G* is an EDT graph.

In summary, we have established that EDT-Recognition is NP-complete. Moreover, the graph *G* constructed in the reduction from the $(C_F,C_R)$-Satisfiability problem is a cograph because it does not contain a $P_4$ as an induced subgraph. Therefore EDT-Recognition remains NP-hard if the input graph is a cograph. $\square$
